# Tracking Affective Language Comprehension: Simulating and Evaluating Character Affect in Morally Loaded Narratives

**DOI:** 10.3389/fpsyg.2019.00318

**Published:** 2019-02-22

**Authors:** Björn ‘t Hart, Marijn E. Struiksma, Anton van Boxtel, Jos J. A. van Berkum

**Affiliations:** ^1^Department of Languages, Literature, and Communication, Utrecht Institute of Linguistics OTS, Utrecht University, Utrecht, Netherlands; ^2^Department of Cognitive Neuropsychology, Social and Behavioral Sciences, Tilburg University, Tilburg, Netherlands

**Keywords:** corrugator EMG, grounded cognition, narrative, emotion, embodiment, affective language, language processing, moral evaluation

## Abstract

Facial electromyography research shows that corrugator supercilii (“frowning muscle”) activity tracks the emotional valence of linguistic stimuli. Grounded or embodied accounts of language processing take such activity to reflect the simulation or “re-enactment” of emotion, as part of the retrieval of word meaning (e.g., of “furious”) and/or of building a situation model (e.g., for “Mark is furious”). However, the same muscle also expresses our primary emotional evaluation of things we encounter. Language-driven affective simulation can easily be at odds with the reader’s affective evaluation of what language describes (e.g., when we like Mark being furious). In a previous experiment (‘t Hart et al., 2018) we demonstrated that neither language-driven simulation nor affective evaluation alone seem sufficient to explain the corrugator patterns that emerge during online language comprehension in these complex cases. Those results showed support for a multiple-drivers account of corrugator activity, where both simulation and evaluation processes contribute to the activation patterns observed in the corrugator. The study at hand replicates and extends these findings. With more refined control over when precisely affective information became available in a narrative, we again find results that speak against an interpretation of corrugator activity in terms of simulation or evaluation alone, and as such support the multiple-drivers account. Additional evidence suggests that the simulation driver involved reflects simulation at the level of situation model construction, rather than at the level of retrieving concepts from long-term memory. In all, by giving insights into how language-driven simulation meshes with the reader’s evaluative responses during an unfolding narrative, this study contributes to the understanding of affective language comprehension.

## Introduction

One of the most enjoyable things about reading is that it allows us to walk a mile in the shoes of characters from the most amazing stories. For example, millions of readers have vicariously lived the life of the Machiavellian Queen Cersei from the Game of Thrones book series^1^. This vicarious experience of “walking a mile in another’s shoes" is more than just a figure of speech. To illustrate, although the reader will likely be sitting down as they read about Queen Cersei walking through the palace gardens of the Red Keep, parts of their cortical (pre)motor areas usually involved in walking will nonetheless be slightly activated (e.g., Hauk et al., 2004; Aziz-Zadeh et al., 2006). Theories of grounded cognition hold that this is because in order to understand what we read, we simulate the meaning of words. Simulation in these cases is taken to involve the neural reactivation of experiential, multimodal traces stored from previous experience with the referents described in the language (e.g., Barsalou, 2008). This simulation could, theoretically, either be part of the retrieval of the meaning of individual words (e.g., “walking”) or be part of the construction of a situation model of a longer text (so as to, e.g., represent the situation referred to by “Cercei is walking”). Whether simulation, either at the lexical or the situation model level, is automatic and strictly necessary for comprehension continues to be debated (e.g., Mahon and Caramazza, 2008; Willems and Casasanto, 2011; Barsalou, 2016; Leshinskaya and Caramazza, 2016), but converging evidence supports the notion that sensorimotor simulation is, at least under some circumstances, involved in language comprehension (Kiefer and Pulvermüller, 2012).

While the example above deals with language referring to motor action, simulation is also said to be involved in affective language processing, i.e., language about, or otherwise relevant to emotion (e.g., Vigliocco et al., 2009; Moseley et al., 2012; Samur et al., 2015). In particular, a large number of facial electromyography (EMG) studies show that affective language processing is accompanied by congruent muscle activation (e.g., Foroni and Semin, 2009; Glenberg et al., 2009; Havas and Matheson, 2013; Künecke et al., 2015; Fino et al., 2016). These studies cover a variety of word classes, including adjectives, nouns, and verb types, and primarily rely on measuring activity of the corrugator supercilii (frowning) muscle, which reliably reflects affective valence of stimuli in a wide variety of input domains, including language stimuli (Larsen et al., 2003). For instance, when we read that Cersei is “frowning” or that she is “angry,” our corrugator will increase in activation. Conversely, when we read that she “smiles” or is “happy,” corrugator activity will decrease. These changes in muscle activation do not necessarily result in observable facial expression, but even in those cases we can still pick up the action potentials in the muscles using surface facial EMG (Tassinary and Cacioppo, 1992). Within the framework of grounded language comprehension this activity is commonly interpreted to be a consequence of simulation in emotional (and the associated motor) systems in the brain.^2^

**FIGURE 1:**
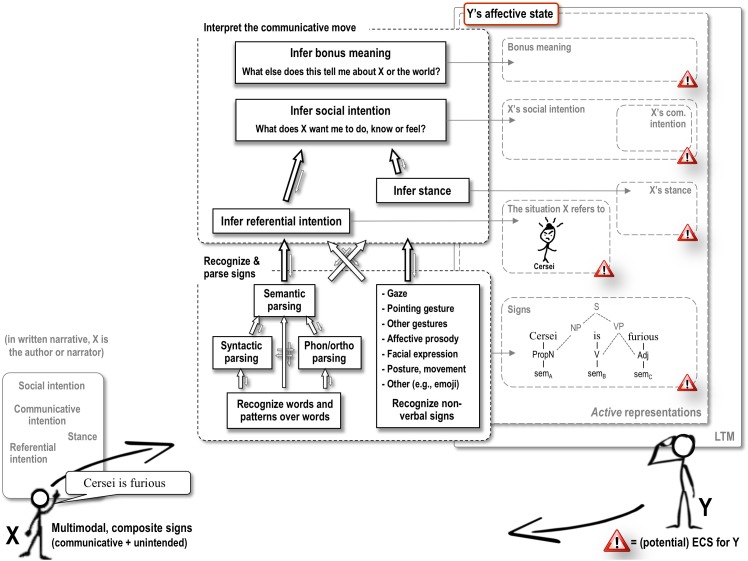
Example processing of “Cersei is furious” in the ALC model. Mental processes and the associated retrieved or computed representations are expanded for addressee Y only. Y’s computational processes draw upon (and add to) long-term memory traces, and involve currently active dynamic representations that reflect what is currently retrieved from LTM, composed from elements thereof and/or inferred from context, in response to the current communicative move. Y’s active representations can be conscious or unconscious. For narratives presented on screen in a laboratory experiment without a foregrounded author or narrator, stance and social intention are presumed to be irrelevant. ECS, emotionally competent stimulus; X’s com. intention, X’s communicative intention. See van Berkum (2018, 2019) for detailed explanation.

However, in everyday language use, affective meaning is routinely more complicated than the facial EMG studies cited above, and many others, allow. van Berkum (2018, 2019) recently outlined the possible interfaces between language comprehension and emotion in a comprehensive model, the *Affective Language Comprehension* (ALC) model. This model helps us to discuss the different ways in which affect can come into play during the processing of a sentence like “Cersei is furious when her favorite dress rips.” As shown in Figure 1, language comprehension is assumed to involve a process of decoding, where comprehenders retrieve and grammatically combine word meanings (as well as recognize other signs, in writing these could include bold font, capitalization, exclamation marks, etc.) and a process of interpretation where comprehenders infer the speaker’s intentions in the context at hand. In line with Tomasello (2008) the latter would include working out the situation to which the speaker is referring and what they are hoping to achieve by doing so. Crucially, in the ALC model, all of these various representations, retrieved or constructed, can all serve as emotionally competent stimuli (ECSs), that is, elicit a conscious or unconscious affective response in the reader.

In terms of this model, affective *simulation* could feature as part of two very different, theoretically distinct subprocesses. One is the recognition and parsing of the composite sign “Cersei is furious,” involving the retrieval of word meanings from long-term memory (sem_A_, sem_B_, sem_C_ in Figure 1) and combining them within grammatical constraints. To the extent that the meaning of “furious” includes traces of actually being furious (e.g., Foroni and Semin, 2009; cf. Pülvermüller, 2013), retrieving that lexical-conceptual meaning can be said to involve affective simulation. The second subprocess that might involve language-driven, affective simulation would be interpreting the referential meaning of what’s being said by the narrator. In this case that would involve identifying the character Cersei in the developing situation model for the narrative, and updating it such that it complies with the semantics of “is furious.” To the extent that updating the situation model entails actually simulating this character being furious, this construction process can also include affective simulation (Zwaan and Radvansky, 1998; Zwaan and Kaschak, 2008; Zwaan, 2014).

As discussed already, affective *evaluation* is something quite different, and involves the reader’s (conscious or unconscious) emotional *reaction to* the various representations that become available as language is recognized, parsed, and interpreted. In the ALC model, this reaction can be an evaluative response to any of the following representations: the narrator’s referential intention (i.e., what is currently foregrounded in the situation model), his or her stance (e.g., distancing, agreeing) and social intention (e.g., informing, persuading, gossiping, entertaining), particular signs he or she is using, and additional inferences triggered by these various representations. In our example, affective evaluation can for example reflect the reader’s feelings toward a character such as Cersei being furious, and, in a richer context, the inferred reasons for how and why somebody is informing him or her about Cersei’s emotional state. Such emotional (and often moral) evaluations of what takes place in the social world around us (or which we discuss in gossip or stories) could be argued to be one of the most important triggers of human emotion (Dunbar, 2004; Greene, 2014).

The fact that language-driven emotional evaluation is independent from language-driven emotion simulation becomes apparent if we take the carefully controlled single words and short sentences typically used in, for instance, facial EMG studies on language processing and embed these inside a story. Imagine reading “Cersei is furious when her favorite dress rips” just a few lines after you have read all about how Cersei ordered an innocent woman to be sent to prison, feeling no remorse and even a certain measure of glee. While the word “furious” still denotes a negative emotion concept, and “Cersei is furious” still refers to a character’s negative state, the latter need not result in a particularly negative evaluation, given what we know about Cersei’s personality. As a matter of fact, because people are inclined to experience Schadenfreude when disliked or envied others experience something negative (Singer et al., 2006; Leach and Spears, 2009; Cikara and Fiske, 2012), we will probably evaluate Cersei’s negative emotion as positive. More generally, we usually do not process language dispassionately. Rather, just as we evaluate what we see, smell, or touch, we evaluate what we read or hear, and we *care* – sometimes quite deeply – about the events described. This raises an interesting question: what will be reflected in corrugator EMG when, as in the example of the furious evil queen ripping her dress, the valence of language-driven simulation conflicts with the valence of our own evaluation of what is described?

### Our Prior Study

In a prior facial EMG study (‘t Hart et al., 2018), we explored this type of conflict by orthogonally manipulating language-driven simulation and moral evaluation of characters within short narratives. In line with the example of Cersei above, we manipulated the moral status of characters in narratives by first describing them as behaving either morally or immorally in a story context. As expected, corrugator measurements revealed that participants quite literally frowned upon immoral actions and relaxed when reading about moral actions. Critically, somewhat later in the story, a so-called *affective event* occurred that caused the same (moral or immoral) character to subsequently experience a positive or a negative emotion.

**FIGURE 2:**
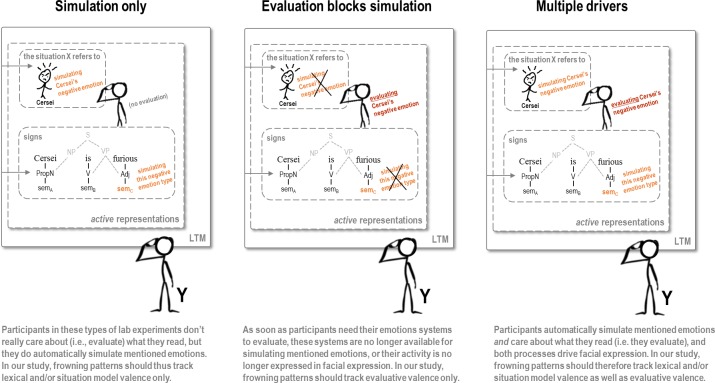
Three possible arrangements of how language-driven affective simulation and evaluation drive the corrugator muscle.

Based on the ALC model, we formulated three possible accounts (Figure 2) of how language-driven simulation and evaluation might contribute to corrugator EMG activity elicited by these subsequent affective events. According to the *simulation-only* account, language-driven simulation will be the sole driver of the corrugator EMG response during the affective event. This account reflects the current interpretation of facial EMG results in embodied language processing research and simply predicts increased corrugator activity (negative affect) when language describes a negative event for the character, and decreased corrugator activity (positive affect) when language describes a positive event for the character, regardless of the character’s moral status.

A second possible account holds that, in stories that are sufficiently interesting to allow for evaluation, emotion-relevant neural systems controlling the corrugator are no longer available for language-driven simulation, and are fully recruited to reflect and express those evaluations. We assume that the salient and dominant evaluation of our stimuli will be in terms of fairness. The *evaluation-blocks-simulation* account thus predicts increased corrugator activity in response to “unfair” events (negative events befalling moral characters and positive events befalling immoral characters), and decreased activity to “fair” events (positive events befalling moral characters and negative events befalling immoral characters).

The third account under consideration allows for a combined influence of language-driven simulation and evaluation on corrugator EMG. This *multiple-drivers* account predicts that both language-driven simulation and evaluation could leave traces in corrugator activity, as indexed by EMG. Because the ways in which these drivers could interact are difficult to predict, we did not formulate a specific expected pattern of corrugator activity, but rather formulated the simple prediction that the corrugator EMG patterns cannot be explained in terms of one of the simpler accounts: *situation-only* or *evaluation-blocks-simulation.*

Our results (‘t Hart et al., 2018) supported the multiple-drivers account. For moral characters we found valence–congruent responses to affective events: increased corrugator activity in response to negative events and decreased activity in response to positive events. For immoral characters, we found neither an increase nor a decrease in corrugator activity as participants read about positive and negative events. In fact, for immoral characters, there was no difference between the corrugator responses to negative and positive events befalling them *at all*. The simulation-only account and the evaluation-blocks-simulation account both predict differential responses to negative and positive events befalling immoral characters (albeit not in the same direction). In the absence of any differential response, we concluded that the multiple-drivers model best accounted for this pattern.

Although the results of our first study supported a multiple-drivers model, replication and extension are in order. For one, the previous study was limited in how the affective event segment was presented. In all the stimulus narratives we used, the affective event segment consisted of a single sentence, presented all at once for 5 s (e.g., Mark is frustrated when after a few minutes he runs out of petrol and becomes stranded by the roadside). Although all sentences were of a similar length and structure, we had no fine-grained control over when participants were reading specific affect-related words (e.g., frustrated). This may have masked potential phasic effects of one or both of the proposed drivers in response to specific affective information inside the sentence.

### The Current Study

In the current study we used the same narratives as ‘t Hart et al. (2018), but presented the critical affective event sentence in a piecemeal fashion; this improves time-locking of the affective information to the corrugator signal and optimizes the chances of finding phasic (short-lived) and possibly temporally distinct effects of language-driven simulation and evaluation. As can be seen in Table 1, the affective event contains two critical segments that are presented separately: an “affective state adjective” describing the emotional state of the character, and an “affect reason segment” detailing the event that led to the character’s emotional state. Because an affective state adjective such as “frustrated” simultaneously presents a highly focused trigger for simulation as well as – by revealing the valence of the event for the character – for evaluation, without being confounded by additional details on the event at hand, it represents the cleanest point at which to assess the interaction of character affect and character morality in corrugator EMG. Furthermore, by also time-locking the EMG signal to the subsequent reason for the character’s emotion, we have a second opportunity to examine the interplay between simulation and evaluation (albeit in a way that is, due to the varying nature of those reasons and their multi-word description, somewhat less precisely controlled).

**Table 1:** Example narrative illustrating trial structure and time on screen for each of 10 different segments.

Baseline		*Neutral distractor image of a forest scene (always the same)*		2 s

Introduction	Mark is driving through the pouring rain, on his way to his mother. He’s still in the inner city and big puddles have formed. It’s been raining non-stop since yesterday. Some streets are practically flooded. There are few cars on the road and fewer bicycles and pedestrians still. Mark is headed for a giant puddle and spots a pedestrian on the sidewalk.			18 s

Character morality (moral/immoral)	Mark slows down to avoid the puddle, making sure he doesn’t splash the pedestrian.	OR	Mark accelerates through the puddle on purpose to create a big splash and soak the pedestrian.	5 s

Continuation	Once outside the city he is driving along on the freeway. There still isn’t a lot of traffic and Mark is enjoying the landscape and the drive. He’s got the radio on full blast and sings along loudly. When he glances at the dashboard to adjust the channel he spots a warning light. He forgot to put petrol in the car and has been running on empty for a while.			15 s

Transition		…		1 s

Name		Mark		0.75 s

Verb		is		0.75 s

Affective state adjective	happy	OR	frustrated	1 s

Neutral		when after a few minutes		2.5 s

Affect reason	he spots a petrol station in time and avoids being stranded.	OR	he runs out of petrol and becomes stranded by the roadside.	2.5 s

		Press “space” to continue to the next story		


*All segments were separated by a blank screen of 250 ms.*

For the character morality segment, we predicted considerably more frowning to immoral actions than to moral actions, reflecting a strong corrugator activity increase to the former, and a small corrugator activity decrease to the latter. As in our previous study, this differential effect would above all indicate that the manipulation of the character’s moral status was successful. For the subsequent – and theoretically critical – affective state adjective, the multiple-drivers model led us to predict an interaction of character morality and event valence. For moral characters, the combined influence of simulation and evaluation should generate an increase in corrugator activity for negative state adjectives (negative state and unfair) and a decrease in corrugator activity for positive adjectives (positive state and fair), resulting in a clear differential valence effect. For immoral characters, the corrugator activity difference between negative and positive state adjectives should be *smaller* and perhaps even absent, because simulation and evaluation should now counteract each other, both in the case of negative state adjectives (negative state but fair) and in the case of positive state adjectives (positive state but unfair).

Note that a multiple-drivers model leaves open the possibility that simulation and evaluation do not affect the corrugator at the exact same moment in time. If simulation is crucial for comprehension, then purely language-driven simulation effects on the corrugator might actually emerge before the first evaluation effects show up. We did not see any evidence for a brief “simulation-only phase” in our earlier study, but any such effects may have been masked by the relatively coarse sentence-level time-locking in that study. Because the current study allows us to time-lock the corrugator EMG response to the critical affective state adjective, we are in a much better position to examine this possibility.

The affect reason segment provides an explanation for the character’s emotional state described in the affective state adjective segment. The affective valence here derives mostly from the meaning of the sub-clause as a whole rather than from particular words, making the time-locking of the corrugator signal to specific affective information less precise. We expect a conceptual replication of our prior study findings (a larger differential valence effect for moral than for immoral characters) here as well, but the main focus, as the cleanest point of measurement, is on the EMG responses to the preceding affective state adjective.

## Materials and Methods

### Participants

Sixty students (12 male) age range 18–27 years (*M* = 21.02, *SD* = 2.62) recruited from the participant pool of the UiL OTS participated in exchange for financial compensation (€12). All were native Dutch speakers, without a diagnosis of dyslexia, without Botox injections to the face, and with normal or corrected-to-normal vision. At the time this research was conducted, the research institute where it took place did not yet have an Ethics Committee (Institutional Review Board), and institute guidelines did not require any other formal ethics approval. Because there is no medical aim involved, the research at hand also did not fall under the scope of national legislation requiring medical ethics review (The Dutch WMO, Medical Research Involving Human Subjects Act). Research procedures complied with The Netherlands Code of Conduct for Academic Practice, as well as with the Declaration of Helsinki. In line with the latter, all of our participants gave written informed consent, based on an elaborate informed consent form detailing the nature of the materials and the procedure, and emphasizing their right to withdraw consent at any time during the experiment without being required to provide a reason, and without losing their right to financial compensation. The informed consent form (in Dutch) is available upon request from the corresponding author.

### Design

The experiment had a fully crossed, 2 × 2, within subjects design: Character morality (moral vs. immoral) and critical event (positive vs. negative). The main dependent variable was *corrugator supercilii* activity as indexed by EMG, measured using surface electrodes (see further description below). *Zygomaticus Major* activity was also measured, but not used as a dependent variable indexing affective valence for reasons further detailed below. Additional measures of individual difference were included, but these were secondary to the main research question. They are therefore reported in Supplementary Data Sheet S1. The stimulus design is illustrated in Table 1 and discussed further below.

### Materials

We presented 64 narratives as outlined in Table 1. Each narrative had four variants based on our 2 × 2 design (morality × critical event). The character morality manipulations were pre-tested and found to be successful in the previous study (‘t Hart et al., 2018). We generated four pseudo-randomized lists of 64 narratives such that (a) each narrative occurred once in one of the four variants in each list, (b) participants would see 16 narratives in each of the four conditions, 8 with a male and 8 with a female main character, (c) average item properties in each list were similar in terms of pro-sociality and expectedness, (d) two lists had the reverse order of the other two lists, and (e) each narrative occurred with both (moral and immoral) male and female protagonists across the four different lists, with the exception of nine narratives that had fixed gender due to stereotypical behavioral expectations. We preceded each narrative with the same neutral distractor image. In doing so we hoped to reduce both intra- and interparticipant variation in baseline corrugator activity compared to the more standard use of a fixation cross. A neutral distractor image gives the participant something to focus on, whereas a fixation cross could lead to mind-wandering.

Unavoidably, the repetitive nature of the experiment will generate some broad expectations for the participants as to how each story unfolds. However, they cannot predict whether a character will behave morally, or immorally, and subsequently experience a positive or negative event and thus this should not influence the corrugator response we are interested in. All Dutch stimulus materials can be found in Supplementary Data Sheet S5.

### Procedure and Data Acquisition

Following informed consent, participants were seated in a comfortable chair in a sound-proof booth and received verbal instruction. Stimuli were presented as specified in Table 1 in Times New Roman (font 26) at a distance of approximately 60 cm. Presentation rate between narratives was self-timed and two longer pauses were inserted to create three, roughly equal blocks. The 64 experimental trials were preceded by two practice trials to acquaint the participant with the procedure.

To maintain compatibility with similar studies addressing emotional valence, we included both corrugator and zygomaticus. However, the zygomaticus does not track emotional valence in the same way (Larsen et al., 2003). Particularly in more complex situations such as our narrative stimuli, smiling activity may be difficult to interpret in terms of pure valence. For instance, smiles can be wry, sarcastic, and smirking as well as expressions of true positive feeling. We therefore focused, in line with ‘t Hart et al. (2018), on corrugator activity and report the zygomaticus data in Supplementary Data Sheet S3 for reference. Facial EMG activity was measured continuously with reusable Ag/AgCl electrodes with a 2 mm contact area over corrugator and zygomaticus muscles on the right side of the face (van Boxtel, 2010). Raw EMG signals were recorded with a NeXus-10 MKII biosignal system (Mind Media) at a sampling rate of 2048 Hz.

After finishing this part of the experiment, electrodes were removed and participants moved to a laptop to fill out some questionnaires. Two questionnaires were included to investigate, in exploratory fashion, potential differences between individuals in the way simulation and evaluation contribute to corrugator EMG activity during online language processing: the Adolescent Measure of Empathy and Sympathy (AMES, Vossen et al., 2015) and the Moral Foundations Questionnaire (MFQ, Graham et al., 2011). Because of the exploratory, secondary nature of this investigation, we report on the associated method and results in Supplementary Data Sheets S1, S5.

After completing the individual differences questionnaires, participants filled out an exit-questionnaire, were debriefed, and given the chance to ask questions. Although due to time-constraints, no comprehension questions were included, answers given in the exit-questionnaire indicated participants had in fact paid close attention to the stories. Finally, participants received the financial compensation and were thanked for their participation.

### Data Preparation and Analysis

The raw data were band-pass filtered between 20 and 500 Hz (48 dB/octave roll-off) and were additionally filtered with a notch filter at 50 Hz (see van Boxtel, 2010), followed by signal rectification and segmentation per narrative using BrainVision Analyzer 2. For each narrative the 2000 ms of baseline activity preceding the narrative, consisting of the same neutral distractor image of a forest scene, were inspected visually for remaining artifacts. We selected maximally long epochs of artifact-free baseline signal, with a minimum length of 500 ms for both muscles simultaneously. If such a 500 ms baseline epoch could not be found, the trial was excluded from analysis (resulting in 1.0% lost trials).

Following baseline selection, the data were exported to MatLab for further segmentation into three parts, time-locked to the onset of the character morality segment (5000 ms), the affective state adjective (1000 ms), and the affect reason (2500 ms). Each of the resulting EMG segments was then divided into consecutive 100-ms bins for a balance between good temporal resolution and sufficient random error reduction (van Boxtel, 2010). The average facial EMG activity during each bin was expressed as a percentage of the average pre-narrative baseline activity level (expressing EMG activity as a percentage of baseline reduces random variance both within and between individuals; van Boxtel, 2010). Supplementary Data Sheet S4 shows continuous average activation for the four conditions in 100 ms bins for an entire trial.

The analysis followed the same procedure as in the previous study (‘t Hart et al., 2018). The three critical segments (character morality, affective state adjective, and affect reason) were analyzed using the Mixed Models Regression procedure (SPSS version 24). Instead of solely looking at average activation over an entire critical EMG segment, we built a growth curve model to also capture linear, quadratic, and cubic trends in the signal. This gives us two flex points and allows us to describe the unfolding corrugator response in some detail, while retaining interpretability and without overfitting (as each additional flex point in principle makes it easier to fit the data (see Peck and Devore, 2008; Mirman, 2014). While models were fitted with a resolution of 100 ms, the parameter estimates (e.g., b for a linear slope) are reported per second for ease of comprehension.

Rather than as a single variable, we included trend components of time for each condition and added these iteratively. For each condition (moral and immoral for the character manipulation, and moral-positive, moral-negative, immoral-positive, and immoral-negative for affective-state-adjective and affect-reason) we generated separate variables for the linear, quadratic, and cubic trends. This afforded us flexibility in building the model and helped us avoid forcing the model to fit, for example, a quadratic trend for all conditions when only some contained a significant quadratic component. In the results we focus mainly on the comparisons of the linear component, as the divergence on this factor was usually enough to discern a differential pattern.

The models included subject and items over lists as random intercepts, and random slopes for the time trend components on the subject factor. Predictors were added iteratively using the -2LL chi-square test (*p* < 0.05), see Supplementary Data Sheet S6 for a complete report. In order to be able to evaluate the corrugator response over time, we first built a growth curve model; linear, quadratic, and cubic trends were added as covariates in the fixed part of the model (the trend components were centered to avoid correlation between trends). To assess the effect of our manipulations on the average corrugator activation over the entire time window, we also added character morality as a fixed factor for the character morality segment. For the affective state adjective we also added valence as a fixed factor and for affect reason segment we added character morality and valence as well as their interaction as fixed factors. With these factors we assessed the differences in *average* activation (centered intercept) between conditions over each critical EMG segment as a whole. However, note that while condition-induced differences in average activation in a particular time windows are of interest, the interpretation of such average differences (or their absence) might be qualified by differences in (linear, quadratic, or cubic) *time trends*, as the latter allow us to investigate the temporal unfolding of corrugator activity in greater detail.

## Results

### Character Morality Segment

**FIGURE 3:**
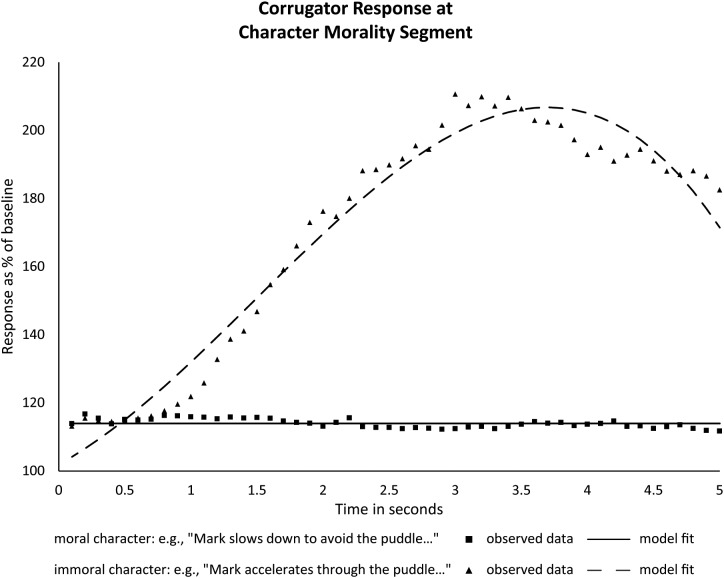
Observed averages of corrugator responses to character morality, with growth curve model regression overlaid.

Figure 3 shows that, as predicted, reading about moral and immoral behaviors evoked a clearly differential corrugator response: Immoral actions elicited a rapid and substantial increase in frowning, whereas moral actions had little effect on frowning^3^. The statistics corroborated this picture. A main effect of character morality indicated a significantly higher average activation for immoral actions than for moral actions during this 5-s time window^4^ [difference *b* = 74.02, *t*(261.87) = 16.40, *p* < 0.001, 95% CI (65.14, 82.91)]. Immoral actions also elicited a significant linear increase in corrugator activity in this time window (*b* = 29.55, *t*(60.00) = 4.17, *p* < 0.001, 95% CI [15.36, 43.73]), as well as significant quadratic and cubic components (see Supplementary Data Sheet S6). Moral actions, on the other hand, did not significantly affect corrugator activity, as reflected by the model fitting a flat line.

In our prior study (‘t Hart et al., 2018), we had actually found a small decrease in corrugator activity to segments describing moral behavior. This finding did not replicate, even though the materials were similar. We suspect that the average impact of our moral behavior passages is rather weak, such that it may surface in one study, but not the next. Many studies have found an asymmetry in responding to positive and negative valence, with weaker and less reliable responses to positive stimuli (for a recent discussion, see Alves et al., 2017). In the particular case of our stories, the actions we designate as moral are probably considered normal behavior and therefore limited in how much positive affect they generate. The immoral actions described in our stories, in contrast, are much more saliently negative, leading to large and reliable corrugator activity increases across studies. Importantly, the presence of considerably more frowning to immoral actions than to moral actions shows that our character morality manipulation was successful, and replicates what was the crucial finding of our previous experiment at this part in the stories.

### Affective State Adjective

**FIGURE 4:**
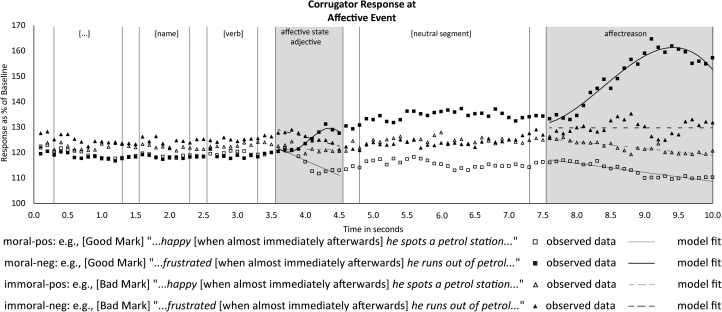
Observed averages of corrugator responses to the affective event, with the two critical segments (affective state adjective and affect reason) highlighted and vertical lines indicating the onset and offset of other segments (including intersegmental 250 ms intervals).

The affective state adjective describes the emotional state of the character (e.g., happy vs. frustrated), and is the first word that reveals the valence of the affective event for the character. In Figure 4, which displays the corrugator activation signal across a 10-s latency range, the first shaded segment indicates the predesignated 1-s time window used for statistical analysis of adjective-elicited results. As for average corrugator activation over this 1 s window, the statistical model revealed a main effect of adjective valence [difference negative–positive *b* = 6.70, *t*(252.70) = 2.93, *p* < 0.01, 95% CI (2.19, 11.20)]. Including character morality as a fixed factor did not improve the model and neither did the interaction between character morality and valence (see Supplementary Data Sheet S6), indicating that character morality did not in any way affect the *average* corrugator activity across the entire EMG segment. However, the model revealed various clear time trends that did significantly differ between conditions. In view of the short (1 s) duration of the predesignated time window at hand, and the fact that readers will need a few hundred milliseconds to visually process and recognize the word at hand, these time trends are the more relevant effects to examine. Our predictions primarily concerned the corrugator response to positive and negative events in relation to character morality. We will therefore discuss the time trends for the moral and immoral conditions separately below.

#### Moral Characters

As can be seen in the first shaded segment of Figure 4, positive and negative states ascribed to moral characters (square markers) elicited a clear differential corrugator activation pattern, with negative state adjectives evoking an increase in frowning activity and positive state adjectives evoking a decrease. The statistical analysis supported this observation (solid lines in Figure 4 show the fitted growth curve model). The moral-negative condition, i.e., descriptions of good people feeling bad, elicited a significant linear increase in corrugator activity [*b* = 23.25, *t*(244.52) = 3.02, *p* < 0.01, 95% CI (8.11, 38.39)], whereas the moral-positive condition, i.e., descriptions of good people feeling good, elicited a significant linear decrease corrugator activity [*b* = -11.75, *t*(37578.69) = -5.15, *p* < 0.001, 95% CI (-16.21, -7.28)]. These two linear trends also differed at the *p* < 0.001 level. In addition to a linear trend component, the EMG signal elicited by the moral-negative condition also contained a significant negative cubic component (see Supplementary Data Sheet S6).

These results are in line with a congruent affective response to positive and negative affective states ascribed to moral characters. This confirms our predictions and replicates the findings from our previous experiment. It also shows that it is possible to pick up a clear corrugator response to a single valenced word in an unfolding narrative.

#### Immoral Characters

Figure 4 shows a slight decrease in activity for the immoral-negative condition and neither an increase nor a decrease in activity during the immoral-positive condition (triangular markers). This pattern also emerged in the statistical analysis (dashed lines in Figure 4 show the fitted growth curve model). The corrugator response in the immoral-negative condition, that is, bad people feeling bad, revealed a significant linear decrease [*b* = -7.18, *t*(60.64) = -2.25, *p* =0.03, 95% CI (-13.54, -0.81)], while for corrugator activity in the immoral-positive condition, that is, bad people feeling good, the model fitted a flat line (see Supplementary Data Sheet S6).

### *Post hoc* Analysis of Neutral Segment

The results at the affective state adjective resemble those of our earlier study (‘t Hart et al., 2018, Figure 1), in that differently valenced state adjectives elicit clearly diverging corrugator signals when they are about moral characters, but not when they are about immoral characters. However, there are two unexpected additional findings: a larger *average* corrugator activation for negative state adjectives relative to positive state adjectives regardless of character morality, and a linearly decreasing corrugator activation at negative state adjectives with immoral characters only. Although the first might be taken to suggest an early simulation-only phase in adjective processing, and the second might be taken to reflect a very rapid onset of Schadenfreude (causing a phasic relaxation of the corrugator that outweighs other factors), we are reluctant to propose these two accounts. This is because, as can be seen in Figure 4, the corrugator signal in the immoral-negative condition is actually already briefly elevated *at the very start* of the presentation of the critical adjective, i.e., at 0 ms after adjective onset, with traces of such elevation even before; this renders the above accounts unlikely.

To further help interpret the effects in the 1-s affective state adjective segment, we performed a *post hoc* analysis of the neutral segment between the affective state adjective analysis window and the affect reason analysis window (see Figure 4, 4.75–7.25 s). As can be seen in Figure 4, the predominant pattern during this neutral segment is a surprisingly stable continuation of corrugator activity levels that were reached at around 1 s after presentation of the affective state adjective. The analysis of corrugator EMG responses in this segment revealed no significant difference between the average activation for immoral-negative and immoral-positive [difference *b* = -0.48, *t*(382.27) = -0.10, *p* = 0.92, 95% CI (-10.42, 9.45)] while the difference between moral-negative and moral-positive remains [difference *b* = 19.17, *t*(382.34) = 3.79, *p* < 0.001, 95% CI (9.24, 29.11)]. In the face of this highly stable pattern of results, we are cautious to over-interpret the modest and short-lived decrease in corrugator activity that we observed to negative state adjectives pertaining to immoral characters.

### Affect Reason

At the affect reason segment the reader learned of the circumstances that led to the character’s affective state, that is, what happened to make the character feel that way. The second shaded segment in Figure 4 above shows the fitted regression lines for the affect reason segment. The model included both character morality and valence as fixed factors, as well as a significant interaction of the two *F*(3,178.90) = 362.78, *p* < 0.001. Pairwise comparisons indicated that all conditions differed significantly (after Bonferroni correction) in average activation, except for immoral-positive versus immoral-negative, and moral-positive versus immoral-positive (see Supplementary Data Sheet S5). However, as can be seen, the average activation does not tell the whole story. A striking aspect of the results was the renewed phasic response during the affect reason segment after the relative stability of corrugator activity patterns after the affective state adjective segment. Our predictions concerned the corrugator response in relation to the valence of the event, depending on character morality. We will therefore discuss the moral and immoral conditions separately, starting once again with the conditions containing moral characters, and focusing on the temporal (trend) aspects of the corrugator response.

#### Moral Characters

For positive and negative events befalling moral characters (squares in Figure 4), a renewed and sizeable differential pattern emerges: a substantial increase in frowning for negative affect reasons and a relatively modest decrease for positive affect reasons. The analysis confirmed this pattern. The model revealed a significant difference between the average activation for moral-negative and moral-positive conditions [difference negative-positive *b* = 41.76, *t*(265.69) = 8.76, *p* < 0.001, 95% CI (29.09, 54.42)]. Additionally, the corrugator response to moral-negative conditions contained a sizeable linear increase in frowning activity [*b* = 18.91, *t*(60.00) = 2.86, *p* < 0.01, 95% CI (5.68, 32.13)], while moral-positive conditions showed a modest, but significant linear decrease of corrugator activity [*b* = -3.51, *t*(94,530.94) = -3.48, *p* < 0.001, 95% CI (-5.49, -1.53)]. The difference between the linear estimates for the two conditions was significant at the *p* < 0.001 level. The model also contained a quadratic and cubic component for moral-negative, but although these trends initially improved the model, they did not ultimately prove to be significant (see Supplementary Data Sheet S6). These results conceptually replicate the findings of our previous study. Moreover, although the magnitude of the differential corrugator response is much larger now, the differential phasic response in the moral conditions is comparable to the one observed at the affective state adjective earlier in the sentence. We return to this magnitude difference in the section “Discussion.”

#### Immoral Characters

As for immoral characters (triangles in Figure 4), the immoral-positive condition elicited a gradual decrease in activity of the corrugator while the immoral-negative condition did not evoke a clear decrease or increase. The difference between the two immoral conditions on average activation overall was not significant [difference negative-positive *b* = 7.79, *t*(250.33) = 1.66, *p* = 0.59, 95% CI (-4.70, 20.27)]. However, the immoral-positive condition was confirmed to evoke a modest but significant linear decrease in corrugator activity [*b* = -2.67, *t*(64,530.94) = -2.65, *p* < 0.01, 95% CI (-4.65, -0.70)]. The model confirmed that the best fit for the immoral-negative condition was indeed a flat line, as none of the time trends significantly improved the model (see Supplementary Data Sheet S6). Taken together, at the affect reason segment, the results indicated a subtle differential development of corrugator activity in immoral-positive and immoral-negative conditions.

## Discussion

This study had two related aims. Firstly, using largely the same materials, we explored the replicability of findings from a previous experiment (‘t Hart et al., 2018); findings that suggested a multiple-drivers account of corrugator activity during online ALC. Secondly, we refined the stimulus design to obtain a temporally more fine-grained assessment of the possible involvement of the two drivers we proposed in our account: language-driven simulation and moral evaluation.

### Reading About Moral and Immoral Behavior

With regards to the character morality manipulation, we predicted a clear effect of morality on the corrugator response. This prediction was confirmed by our results: participants frowned much more at immoral actions than at moral actions. The presence of a differential effect for corrugator EMG in response to moral and immoral behavior replicates our earlier findings. It also lends further support to the link between corrugator activity and moral valence, and as such extends our knowledge of how facial EMG relates to affectively salient language (e.g., Havas et al., 2010; Foroni and Semin, 2013; Fino et al., 2016). The successful character morality manipulation also meant that the stage was set for a fairness-based evaluation of the subsequent critical segments.

### Reading About Character Affect

The critical event sentence contained two segments that conveyed affectively salient information. During the first, the state adjective, readers discovered how the protagonist felt about the event (“happy” vs. “frustrated”). Under the multiple-drivers account (‘t Hart et al., 2018), we predicted that, for moral characters, simulation and evaluation would align in terms of valence and thus lead to a clear differential corrugator response to negative and positive emotions ascribed to them. This was precisely what we found: corrugator EMG displayed a clear differential response to good people experiencing negative emotions (negative for the character and evaluated negatively, as unfair) and positive emotions (positive for the character and evaluated positively, as fair).

For immoral characters, the multiple-drivers account predicted that simulation and evaluation would counteract each other and this would attenuate the resulting net differential response to positive and negative state adjectives. Figure 4 reveals that this is indeed the case, with the corrugator signals to these two adjective types staying much closer to each other when the character had behaved immorally earlier, than when he or she had behaved morally. This pattern fits with an explanation in terms of counteracting forces of simulation and evaluation. For immoral characters experiencing a negative emotion, we did also find a small phasic decrease in frowning activity at the negative state adjective *relative to pre-adjective corrugator activity*. Although this transient response could be taken to reflect a very rapid onset of Schadenfreude, we already noted that the corrugator signal in the immoral-negative condition was actually already briefly elevated at 0 ms after adjective onset, with traces of such elevation even before. We therefore hesitate to overinterpret this particular phasic response, at least until replicated in future studies.

Regardless of whether the phasic decline in corrugator activity in the immoral-negative condition should be taken seriously or not, the overall pattern of results that emerged in response to the affective state adjective and continued for seconds thereafter – a strong differential corrugator EMG response to positive and negative events befalling moral characters, but a much attenuated differential corrugator EMG response to positive and negative events befalling immoral characters – replicates and extends our earlier 2018 finding. This supports our hypothesis of a combined influence of both evaluation and simulation on corrugator activity while processing affective language, rather than any one single driver controlling the corrugator.

We noted in the “Introduction” that the multiple-drivers account leaves open the possibility that simulation and evaluation do not affect the corrugator at the same time, but might do so successively. We reasoned that the best chances of finding such a brief “simulation-only phase” would be at the start of the affective state adjective segment. If simulation were initially the sole driver for corrugator activity, we would have expected a briefly increased corrugator activity for negative states and decreased corrugator activity for positive states, regardless of character morality. There was a main effect of adjective valence on the average corrugator signal in the 1-s time window, but, like the downward trend discussed in the previous section, this actually reflects an earlier elevation of the EMG signal in the immoral-negative condition, already present at 0 ms after adjective onset. Given that basic visual and lexical processing will take a few hundred milliseconds, this should thus *not* be taken as an indication of a brief simulation-only phase in adjective-elicited processing. Instead, the overall data pattern is most compatible with versions of the multiple-drivers account where simulation and evaluation both influence corrugator activity and counteract each other in the case of the immoral character conditions.

### Reading About the Reasons for Character Affect

The second affectively salient segment of the critical event manipulation gave the reason for the affective state in the earlier critical segment by describing the event that evoked it. The affective information was spread out over a sub-clause and thus the time-locking was less precise compared to the affective state adjective segment. We predicted to find evidence in support of the multiple-drivers account: i.e., that the differential corrugator response to positive and negative events befalling immoral characters would be attenuated compared to moral characters, as in ‘t Hart et al. (2018). This was in fact what we found. Bad things happening to bad people resulted in neither an increase nor a decrease in corrugator EMG, while description of bad characters experiencing a positive event elicited a decrease in activity (indicative of positive affect). Taken together this resulted in a much reduced differential corrugator pattern (compared to good characters) in response to bad characters experiencing positive and negative events, replicating previous findings and providing further support for the multiple-drivers model.

It is interesting to note that, on top of the differential pattern elicited by the affective state adjective, the affect reason segment elicited another sizeable phasic corrugator EMG activity. This shows that the affect reason segment was in fact a salient source of additional affective information. Moreover, the amplitude of the response during the reason segment is much larger than during the affective state adjective, suggesting that perhaps the full affective potential can be modulated by the reason for the affective state of the character. This makes sense, as fairness in terms of “everybody should get what he or she deserves” possibly evokes the largest emotional responses when readers learn what the protagonist at hand *actually* “gets.” The possibility that evaluation of the affective state adjective alone is incomplete and requires knowledge of the reason could also be further investigated by manipulating the severity of moral transgressions and the intensity of the affective events that follow.

### Between Character Affect and Its Reasons

The 2500 ms neutral segment following the affective state adjective is intriguing because corrugator activity is stable for all four conditions. This offers a tantalizing hint as to what manner of language-driven simulation is involved: lexical-conceptual meaning (e.g., “frustrated” or “happy”) versus building a situation model (imagining Mark being furious; cf. Zwaan and Radvansky, 1998; Zwaan and Kaschak, 2008). What could be the source of such enduring stability? It seems unlikely that the source would be lexical-conceptual simulation, as this segment does not contain any particularly affectively salient lexical items. Moreover, such simulation in service of the retrieval of a single lexical item would arguably be more short-lived in nature. Rather, this points to simulation *at the situation model level*, where readers are maintaining a stable, active representation of the situation constructed thus far, as they wait to find out the final piece of affective information. Note that under a multiple-drivers interpretation of our central pattern of findings (large frowning differences between negative and positive state adjectives pertaining to moral characters, but not between negative and positive state adjectives pertaining to immoral characters), the stability over time also suggests that the *evaluation* of this situation model is momentarily stable, possibly in expectation of the reason for the previously described affective state of the main character. Consistent with this idea, we do see a shift in the corrugator pattern when this reason becomes apparent, with a small differential pattern emerging even for immoral characters. This could be taken to indicate what might be expected anyway: that the net outcome of the counteracting forces of simulation and evaluation dynamically changes in relation to what is being asserted at any point in an unfolding sentence.

### Open Questions

Although our results support the multiple-drivers account, in which language-driven simulation and (in our case fairness-based) evaluation have independent, and in some cases counteracting, effects on corrugator activity, we can imagine an alternative account being put forward where readers selectively simulate affective meaning depending on whether they have identified with the character or not. Identification as a concept is defined by Oatley (1999) as “taking on the character’s goals and plans [as a result of which the audience] experiences emotions when these plans go well or badly.” Several studies have demonstrated a relationship between character likeability (similar to our character morality manipulation) and the degree of identification with these characters (Tal-Or and Cohen, 2010; Chory, 2013). Other work showed that the level of identification with a character influenced the degree to which readers reported experiencing emotions (both positive and negative) in response to, or evaluation of, the vicissitudes befalling that character (Hoeken and Sinkeldam, 2014).

While we by no means exclude the possibility that identification is a potentially relevant and interesting factor, we do not as yet see how identification-mediated selective simulation, or selective evaluation, in the case of moral characters *only*, *alone* could account for our results. For one, explaining the absence of differential responses for immoral characters at positive and negative state adjectives and affect reasons (in terms of equal corrugator activity levels for both event polarities, but also in terms of equal pre- and post-event corrugator activity level *within* a single item type) as reflecting the absence of simulation at the lexical and situation model level would mean that readers in those cases also did not at all *evaluate* what they read. With morally loaded stories such as ours, this seems unlikely; if evaluation would shut down for events befalling bad characters, gossip, for example, would be a lot less effective, Schadenfreude would not exist, and TV series would become a lot less engaging. Also, research in moral psychology has shown that fictional scenarios very easily elicit emotional responses in the lab (Greene, 2014). These same observations also argue against an identification-mediated selective *evaluation*-only account.

Of course, this line of reasoning, while in line with the data, is *post hoc* and builds on the absence of a difference, a null result. However, there is also some positive empirical evidence that contradicts these selective, identification-mediated simulation-only or evaluation-only accounts. As discussed above, the analysis of the reason segment revealed a differential pattern for immoral-positive and immoral-negative. In addition, we found that during the affect reason segment the linear decrease for immoral-positive did not differ significantly from moral-positive [difference moral-pos - immoral-pos *b* = -0.83, *t*(94,530.94) = -0.58, *p* = 0.56, 95% CI (-3.63, 1.96)] and neither did the difference in overall average activation [difference moral-pos - immoral-pos *b* = 9.18, *t*(250.20) = 1.96, *p* = 0.31, 95% CI (-21.66, 3.30)]. This suggests that positive affect reasons elicited a similar corrugator response, regardless of the moral identity of the character. This identical corrugator response indicating positive affect clearly speaks against an account where emotions of immoral characters are simply not simulated or evaluated due to a lack of identification.

Another matter where the way individuals respond might provide further insight into the way readers respond to the stimulus materials is the participant’s expectation of events according to just-world beliefs and good things (should) happen to good people and bad things to bad people (e.g., Hafer and Bègue, 2005). This might be another dimension where individual differences play a role and the degree to which participants hold just-world beliefs would be an interesting matter to explore, especially in light of the connection that has been made between just-world belief and fictional narratives (e.g., Appel, 2008).

Ultimately the support for the multiple-drivers account rests, in part, on the fact that our data are not in line with the two alternative models. Future studies should test the multiple-drivers account more directly by attempting to selectively manipulate the relative contributions of language-driven simulation and evaluation to corrugator activity. This can perhaps be done by systematically manipulating the severity of immoral behavior, and, hence, the force of evaluation.

Another important open question involves the role of facial mimicry. Research has shown that in face-to-face interaction, people mimic each others’ facial expression in fairly automatic ways (e.g., Hess and Fischer, 2014), and that such mimicry can depend on the relationship between interactants (e.g., McHugo et al., 1991; Hess and Bourgeois, 2010; Stel et al., 2016). Whether such – originally visual – mimicry can arise in response to a language-elicited imagined situation is not clear, but we should at this point not exclude this possibility. Linguistically driven facial mimicry poses an interesting challenge to the theoretical framework we are operating in, as it could be conceived of as an instance of emotional evaluation (e.g., as a rudimentary form of empathy, see, e.g., Decety and Cowell, 2014), but perhaps also as a form of simulation (because facial mimicry might help us “imagine” what other people feel). Future work may be able to specifically target the role of facial mimicry in language comprehension, and, if needed, help us understand how to accommodate it in frameworks such as the ALC model.

## Conclusion

The results from all three critical segments broadly replicate the findings from our previous study (‘t Hart et al., 2018). We saw once more that readers literally frown upon morally objectionable actions described in a story. When participants subsequently read about the emotions of characters and the reasons for these emotions, we once more saw evidence that complex language comprehension involves the emotion system in both the construction of meaning and in our own emotionally response to what is described. Despite the improved time-locking, we found no evidence, in any of the affective event segments, for a phase where the emotion system is engaged only in language-driven simulation. These results lend support to the multiple-drivers account previously proposed in ‘t Hart et al. (2018), where language-driven simulation and evaluation independently, and in some cases in opposition to each other, influence corrugator activity. However, they also raise new questions, about the potential role of identification and facial mimicry, and, related, about the exact nature of language-driven simulation.

The interesting stability in corrugator activity levels during the neutral segment (e.g., “when after a few minutes”) following the adjectival description of the character’s emotional state and the reason for it also extends out earlier findings in new directions. In particular, this suggests that the language-driven simulation component that our studies tap in to involves simulation at the situation model level, rather than simulation in service of lexical-conceptual retrieval. Also, the emergence of a different pattern of corrugator results when readers finally encounter the reason for the character’s emotions supports suggests that the balance between simulation and evaluation, or perhaps the nature of the evaluation, develops as the narrative unfolds, and is indexed in real-time by the corrugator response. In all, our results testify to the complexity of the interface between language and emotion, as well as to the utility of facial EMG to explore this.

## Author Contributions

B‘tH, MS, and JvB designed the study and wrote the paper. B‘tH conducted the study. B‘tH and MS analyzed the results. AvB provided specific EMG expertise for study design and data analysis.

## Conflict of Interest Statement

The authors declare that the research was conducted in the absence of any commercial or financial relationships that could be construed as a potential conflict of interest.

## Footnotes

1 The examples given in this paper do not contain spoilers about events from either the books or the TV show.

2 There is a related line of evidence from studies employing methods to inhibit or block corrugator activity showing that participants are slower to process or comprehend affective language related to negative emotion (Havas et al., 2010) or negative facial expressions (Baumeister et al., 2016). However, whereas this research examines the potential *causal role* of embodiment in comprehension, we use facial EMG to simply *track* the occurrence of embodied aspects of language processing, as an index of emotion simulation.

3 The corrugator responses in Figure 3 start slightly above the 100% baseline level. We suspect this reflects the additional effort demanded by reading the preceding introduction segment of the narratives, in comparison with passive viewing of a preceding neutral image during baseline

4 Because our growth curve analysis used centered time components, the intercept in our analyses represents the average activation over the entire time window, rather than the point where the curves leave the *Y*-axis. Furthermore, whereas the reported intercepts are based on the fixed effects only, the fitted line in Figure 3 includes both the fixed effects and the random effects.

## Supplementary Material

DATA SHEET S1 In our previous study (‘t Hart et al., 2018) we found subtle differences in the way the corrugator responded to the manipulation of character morality and affective event valence based on scores from the Adolescent Measure of Empathy and Sympathy (Vossen et al., 2015).

DATA SHEET S2 Experimental stimuli List 1.

DATA SHEET S3 Observed averages in 100 ms bins *zygomaticus major* per condition for all three critical segments.

DATA SHEET S4 Whole trial observed average corrugator supercilii activity in 100ms bins over all trials, split into the four conditions (note: the positive/negative distinction is irrelevant at the character manipulation stage).

DATA SHEET S5 Individual Differences.

DATA SHEET S6 Iterative model report for Character Morality Manipulation - Corrugator. Each line reports the assessment of model fit after adding a single predictor.
