# Supplementary material for: Tracking Affective Language Comprehension: Simulating and Evaluating Character Affect in Morally Loaded Narratives
Source: Front Psychol. 2019 Feb 22;10:318. doi: 10.3389/fpsyg.2019.00318 (PMC6398452; doi:10.3389/fpsyg.2019.00318)
Supplement: DATA SHEET S1 — In our previous study (‘t Hart et al., 2018) we found subtle differences in the way the corrugator responded to the manipulation of character morality and affective event valence based on scores from the Adolescent Measure of Empathy and Sympathy (Vossen et al., 2015). [file Data_Sheet_1.PDF]

Supplementary Materials 1

In our previous study ('t Hart B. , Struiksma, van Boxtel, & Van Berkum, 2018) we found subtle differences in the way the corrugator responded to the manipulation of character morality and affective event valence based on scores from the Adolescent Measure of Empathy and Sympathy (Vossen, Piotrowski, & Valkenburg, 2015). This questionnaire measures three components of pro-social emotion: cognitive empathy (*understanding* what the other feels), affective empathy (*feeling* what the other feels), and sympathy (feeling *for* the other). In our previous experiment we found that the higher participants scored on Affective Empathy the more negative affect the corrugator reflected at immoral actions and at unfair affective events (moral-negative and immoral-positive).

We have also included the Moral Foundations Questionnaire (Graham, et al., 2011) to investigate whether participants' attachment to the morality dimension of fairness influenced the corrugator response. We suspected fairness to be an especially salient moral dimension because the evaluation of the affective event hinges on whether it is considered *fair* or *unfair* in light of the protagonist's moral status.

We calculated an average score for each subscale of the AMES for each subject, ranging from 1 to 5. To investigate the effect of each of these individual measures on the fEMG response, we used the mixed models procedure. We analysed individual differences in average corrugator activation to each of the three segments by including each personality trait as a continuous covariate in the fixed part of a simplified model without time components. The personality traits included affective empathy ($M$ = 3.04, range = 1.25-4.25), cognitive empathy ($M$ = 3.56, range = 2.25-5.00), sympathy ($M$ = 3.98, range = 2.33-5.00) and fairness ($M$ = 3.66, range = 2.50-4.80). A full report of all the analyses can be found in Supplementary Materials 6 , we only mention the significant results below.

At the character morality segment we found an effect for affective empathy only. Our analysis revealed that if participants scored higher on the affective empathy scale, they tended to frown less in response to immoral actions ($b$ = −26.98, $t$ (60.22) = −2.52, $p$ = .014, 95% CI [−48.39, −5.59]). This result not only did not replicate our finding from the previous experiment, it actually displayed the exact reverse effect; higher affective empathy scores led to more rather than less frowning during immoral actions.

At the affective state adjective segment corrugator activity only interacted with cognitive empathy, and there only for the moral-negative. The effect was such that the higher participants scored on cognitive empathy, the less they frowned at bad things happening to good people ($B$ = −11.73, $t$ (65.17) = −2.81, $p$ < .01, 95% CI [−20.07, −3.40]). Higher cognitive empathy scores indicate a better developed skill at understanding how people feel. Of course, a better understanding of how someone feels does not necessarily mean that you will feel bad for them (sympathetic response) and feel what they feel (affective empathetic response). This effect of cognitive empathy was new, we found no such effects in our previous study, although there we were unable to test the effect of these traits on the affective state adjective separately.

At the affect reason segment we found an effect of three traits for moral-negative, i.e., bad things happening to good people. First, we once again found an effect of cognitive empathy for moral-negative. This effect was in the same direction as for the affective state adjective segment: higher cognitive empathy scores meant less corrugator activity ($B$ = −26.03, $t$ (63.38) = −4.36, $p$ < .001, 95% CI [−37.98, −14.09]).

Secondly, increasing affective empathy scores corresponded to a decrease of negative affect on the corrugator. This result is similar to that during the character morality segment ($B$ = −10.96, $t$

(63.92) = −2.05, $p$ = .045, 95% CI [−21.65, −0.27]). Affective empathy measures the tendency of people to feel what another feels, we would therefore expect more negative affect for good characters meeting misfortune for participants who scored higher on this trait. In fact, we found precisely that previously ('t Hart B. , Struiksma, van Boxtel, & Van Berkum, 2018). The result here represents is the opposite.

The third individual trait that revealed an effect on corrugator activity in response to moral-negative conditions was fairness. This measure reflected how strongly a person adhered to the moral dimension of fairness. Here the effect seemed more expected; the more people adhered to fairness, the more they frowned at bad things happening to good people, which is unfair ($B$ = 28.42, $t$ (65.04) = 4.65, $p$ < .001, 95% CI [16.22, 40.62]). However, higher scores for fairness also led to more frowning in response to immoral-negative conditions, which might be considered fair as it concerns bad characters experiencing something bad ($B$ = 13.92, $t$ (65.11) = 2.28, $p$ = .026, 95% CI [1.71, 26.14]).

All in all, the results of the individual differences present a mixed bag. We find some effects that go against not only our expectations, but also the results obtained in a previous experiment with near enough the same materials. For this reason, as well as the correlational nature of these results, we are wary of drawing strong conclusions based on these analyses. While we maintain that these processes of simulation and evaluation could be subject to some individual variation, our conflicting results as well as the correlational nature of the evidence do not give us a solid foundation to make any claims regarding individual differences at this time.
