# Supplementary material for: Tracking Affective Language Comprehension: Simulating and Evaluating Character Affect in Morally Loaded Narratives
Source: Front Psychol. 2019 Feb 22;10:318. doi: 10.3389/fpsyg.2019.00318 (PMC6398452; doi:10.3389/fpsyg.2019.00318)
Supplement: DATA SHEET S2 — Experimental stimuli List 1. [file Data_Sheet_2.PDF]

Supplementary Information 2

Experimental stimuli List 1

Number	Introduction	Character Manipulation	Continuation	Transition	Name	Verb	Affective State Adjective	Neutral Segment	Affect Reason
1	Sandra heeft al een tijdje een conflict met haar buren. Ze zet haar auto vaak op de parkeerplek voor de deur van haar buren. Haar buurman en buurvrouw beweren echter dat die plek speciaal voor hun auto is. Op een dag hoort Sandra van de andere buren dat haar buurman verongelukt is. Sandra aarzelt niet en loopt direct naar het huis van haar buren. Ze belt aan en haar buurvrouw doet de deur open.	Sandra bijt haar toe dat haar man toch een vervelende vent was en het waarschijnlijk verdiende om dood te gaan.	Sandra loopt weer naar huis en gaat verder met koken. Die avond pakt Sandra haar tablet en leest het financiële nieuws. Sandra heeft kort geleden veel van haar spaargeld in aandelen gestoken. Ze houdt sindsdien de markt goed in de gaten en nu maakt ze zich zorgen. Ze heeft slechte berichten gehoord en checkt de koersen van de aandelen in haar portfolio.	…	Sandra	is	dolblij	als ze de gegevens doorleest	en haar aandelen in waarde verdubbbeld zijn
2	De afdeling waar Wesley werkt heeft het heel erg druk. De opdrachten blijven maar binnenstromen. Daarom is er kort geleden een nieuwe medewerker aangenomen om het team te versterken. De nieuwe medewerker is een jong en knap meisje dat pas afgestudeerd is. Op een dag komt Wesley haar alleen tegen in het kopieerhok. Ze staat bij het kopieerapparaat en heeft duidelijk ruzie met het apparaat.	Wesley gaat achter haar staan, vraagt wat er is, en streelt zogenaamd per ongeluk de billen van het meisje.	Grijnzend werkt Wesley die middag verder en gaat om vijf uur naar huis. Thuis aangekomen parkeert hij zijn auto en loopt naar de voordeur van zijn huis. Wesley hoopt dat bij de post de dvd zit die hij besteld had. Hij had de hele avond vrijgehouden om het nieuwe seizoen van de tv serie te kijken. Wesley doet de deur open en ziet een stapel post op de deurmat.	…	Wesley	is	blij	wanneer hij de post doorbladert	en de dvd erbij zit en hij dus door kan kijken
3	Jeffrey komt na een dag hard werken thuis. Net op het moment dat hij de voordeur achter zich dicht trekt begint het te onweren. Het bliksemt en begint hard te regenen. Dan ziet Jeffrey dat er die dag post is bezorgd bij hem. Tussen de folders en rekeningen die zijn bezorgd ziet Jeffrey een rouwkaart liggen. De kaart blijkt verkeerd bezorgd te zijn en eigenlijk bestemd voor zijn overburen.	Jeffrey heeft geen zin om zich nat te laten regenen en verscheurt de rouwkaart en gooit hem in de prullenbak.	Het noodweer blijft die dag aanhouden. Jeffrey hoort dat het KNMI code rood afgeeft en aanraadt binnen te blijven. Het begint hard te hagelen en Jeffrey ziet buiten enorme hagelstenen uit de lucht vallen. Een hagelsteen zo groot als een golfbal landt in Jeffreys tuin en Jeffrey vreest voor zijn nieuwe auto. Zodra het droog is gaat Jeffrey de schade opnemen.	…	Jeffrey	voelt	opluchting	wanneer hij bij de auto aan komt lopen	en deze geen beschadigingen blijkt te hebben
4	Emma loopt over de Dam richting de Kalverstraat. Ze moet een verjaardagscadeautje halen voor een vriend. Het is gelukkig niet heel druk, maar het loopt al tegen sluitingstijd. Op de hoek van de Kalverstraat zit een blinde straatmuzikant. Hij speelt de sterren van de hemel en zingt met een doorleefde stem. Op de gitaarhoes voor hem ligt al heel wat kleingeld en zelfs een paar briefjes.	Emma heeft alleen een tientje, maar de muzikant is zo goed dat ze het hem gunt, hij kan het vast gebruiken.	Later die week is de verjaardag waarvoor ze op pad was. Er zijn al gauw 25 mensen en het feestje is in volle gang. Emma kent echter niet veel van de mensen die er zijn, en bekenden zijn er nog niet. Ze probeert zich in het gesprek van een groepje naast zich te mengen. Emma gaat erbij staan en wanneer een stilte valt doet ze ook een duit in het zakje.	…	Emma	voelt	blijdschap	opkomen wanneer het groepje	haar gelijk welkom doet voelen
5	Ilse staat te wachten bij de pinautomaat. Er staat één man voor haar en niemand achter haar. Terwijl ze wacht kijkt Ilse wat op haar telefoon en bedenkt hoeveel geld ze op zal nemen. Vanuit haar ooghoek ziet Ilse dat de man voor haar bijna klaar is. Ilse stopt haar telefoon weg en pakt haar portemonnee. Als de man wegloopt, ziet Ilse een briefje van 50 vallen maar de man heeft niks door.	Ilse roept de man gauw na en pakt het briefje van 50 op om terug te geven, de man bedankt Ilse uitvoerig.	Later die avond is Ilse onderweg naar huis. Ze bedenkt zich ineens dat ze niks meer voor het ontbijt in huis heeft. Ze kijkt hoe laat het is en gaat meteen wat sneller lopen. Er is gelukkig een Albert Heijn vlakbij, maar die gaat al bijna dicht. Als Ilse de hoek om loopt ziet ze hoe een medewerker een bord binnenhaalt en ze gaat nog sneller lopen.	…	Ilse	is	dankbaar	wanneer de medewerker haar ziet	en toch nog even snel binnenlaat
6	Martijn fietst door de stad naar de sportschool. Hij heeft een lange dag achter de rug en heeft zin om intensief te sporten. Martijn is halverwege wanneer hij door een erg smal steegje fietst. Een bejaarde man loopt langzaam in dezelfde richting door het steegje. In het voorbijgaan schampt Martijn met zijn fiets de oude man. De man komt ten val en Martijn hoort hem kermen van de pijn.	Martijn beseft dat hij fout zat, maar doet alsof er niks gebeurd is en fietst door, laat een ander die ouwe helpen.	De volgende dag staat Martijn vroeg op. Hij heeft een sollicitatiegesprek bij een bedrijf voor een leidinggevende functie. Het bedrijf is gevestigd in een nabijgelegen stad, 20 kilometer verderop. Omdat Martijn geen rijbewijs heeft reist hij met openbaar vervoer. Hij wil voor de zekerheid een trein eerder nemen en zorgt dat hij op tijd op het station is.	…	Martijn	is	euforisch	wanneer hij op het perron komt en	op tijd blijkt te zijn en de juiste trein haalt
7	Maria loopt met een ingepakt cadeau over straat. Ze is onderweg naar een kraamvisite bij een collega. Maria heeft niks met baby's en gelukkig heeft iemand anders het cadeau uitgezocht. Maria belt aan en het duurt even voordat de deur opengaat. De kamer zit vol met familie en vrienden die ze niet kent. De baby wordt uiteraard meteen getoond en Maria vindt het maar een lelijke baby.	Maria zegt desgevraagd dat het een mormel is en dat ze sowieso niet snapt wat er mooi is aan baby's.	Wanneer Maria bij de kraamvisite vandaan komt loopt ze het centrum in. Ze heeft honger en gaat op zoek naar iets warms voor de lunch. Ze schiet een döner tent binnen en bestelt een broodje döner. Ze gaat zitten en valt meteen aan wanneer het broodje klaar is. Bij de eerste hap valt er gelijk een enorme klodder saus uit de zijkant van het broodje.	…	Maria	is	nijdig	als de klodder gemorste saus	op haar nieuwe blouse valt en dus verpest is
8	Stefan zit achter de computer op het werk. Hij is bezig concertkaartjes te bestellen voor hem en drie collega's in zijn team. Ze gaan wel vaker met z'n allen iets doen buiten het werk om. Het is ondertussen meer een vriendengroep dan collega's. Er is een nieuw teamlid dat er nog niet echt bij hoort. Dat nieuwe teamlid komt even later binnen en zegt ook fan te zijn van die band.	Stefan nodigt hem uit om mee te gaan, zodat hij zich meer thuis gaat voelen als nieuweling binnen een hecht team.	Tegen half vijf besluit Stefan dat het leuk is geweest voor die week. Hij sluit zijn computer af en pakt zijn spullen bij elkaar. Voor vijven is hij op het station en wacht hij op de trein. Het nadeel van op tijd weggaan op vrijdag is dat hij niet de enige is. Het is hartstikke druk op het perron en hij ziet dat de binnenkomende trein ook al goed vol zit.	…	Stefan	is	boos	wanneer in het gedrang voor de inagng	iemand hem zonder pardon omver duwt
9	Sanne zit naar een voorstelling onder regie van een vriend te kijken. Het stuk is ongeveer halverwege en Sanne is al volledig uitgecheckt. De acteurs zijn niet bijster goed en het decor is amateuristisch. Ook het verhaal zelf kan niet echt boeien. Sanne heeft sterk de neiging om halverwege naar huis te gaan. Maar ze blijft toch tot de receptie en komt daar haar vriend, de regisseur, tegen.	Sanne schudt hem de hand en steekt vervolgens van wal en vertelt keihard hoe slecht ze het stuk vond.	Na de receptie loopt Sanne richting het dichtstbijzijnde grote plein. De trams rijden al niet meer en het begint steeds harder te regenen. Eenmaal bij het plein aangekomen wil ze een taxi aanhouden. Er zijn echter weinig taxi's en veel mensen die een taxi willen vanwege de regen. Eindelijk weet ze een taxi aan te houden en deze stopt vlakbij haar.	…	Sanne	is	verontwaardigd	wanneer de taxi vlak voor haar stopt	en iemand haar opzij duwt en gauw instapt
10	Lieke zit met een vriendin op het terras. Het is donderdagmiddag en ze hebben allebei lekker vakantie. Haar vriendin moet naar het toilet, waar het waarschijnlijk stervensdruk zal zijn. Terwijl Lieke alleen zit komt een vriend van haar langsfietsen. Hij vertelt Lieke over een feestje vanavond en moet gelijk verder. Lieke wil wel heen, maar ze heeft eigenlijk al plannen gemaakt met die andere vriendin.	Lieke vertelt eerlijk over het feestje en vraagt of de vriendin het leuk zou vinden om daarheen te gaan.	Later die middag springt Lieke gauw onder de douche thuis. Onder het afdrogen bedenkt ze zich dat ze cash geld nodig zal hebben. Dat herinnert haar eraan dat haar salaris één dezer dagen binnen moet komen. Nadat ze zich heeft aangekleed rommelt ze in de la om de random reader te vinden. Eenmaal gevonden logt ze gauw in om haar saldo te checken.	…	Lieke	constateert	nors	dat de salarisadminstratie	weer eens heeft gefaald, nog geen geld
11	Sarah heeft de nachtdienst op de verpleegafdeling. Het is tijd om een ronde te lopen en haar collega en zij doen elk de helft. Sarah controleert alle infusen en kijkt of iedereen goed slaapt. Ze gaat stilletjes van kamer tot kamer. Op één van de laatste kamers ziet ze een portemonnee liggen. Hij is van de oudere man die vanmiddag zijn kleinkinderen met hun rapporten op bezoek had.	Sarah kijkt snel om zich heen, haalt er 50 euro uit en vervolgt haar ronde, de man heeft toch niet lang meer.	Aan het einde van haar dienst gaat Sarah gauw richting huis. Eenmaal thuis zet ze een kop thee en zet haar laptop aan. Met de laptop in één hand en de thee in de andere loopt ze richting de woonkamer. Ze ziet door het voorraam dat de zon ondertussen op is gekomen. Dan struikelt ze ineens over de drempel en laat de laptop vallen en morst thee.	…	Sarah	constateert	pissig	dat de laptop als ze hem weer aanzet	helemaal niks meer doet, compleet kapot
12	Danny is aan het werk in de werkplaats van zijn fietsenzaak. Hij is bezig een achterwiel te vervangen. Het is halverwege de ochtend en nog lekker rustig. Wanneer de bel gaat maakt Danny snel af wat hij aan het doen was. Hij veegt zijn handen schoon en loopt dan snel naar voren. Er staat een vrouw met een hoofddoek te wachten en het blijkt al gauw dat haar Nederlands niet heel goed is.	Danny schudt meewarig het hoofd, negeert de vrouw straal en gaat weer verder in de werkplaats, domme allochtonen.	Later die dag, na de lunch, worden er onderdelen geleverd. Danny tekent voor ontvangst en begint de pakketjes te sorteren. Er is één bepaald onderdeel waar hij al een hele tijd op zit te wachten. Het is een heel speciaal onderdeel voor een luxe mountainbike. Het onderdeel is al twee keer verkeerd geleverd en de klant begint ongeduldig te worden.	…	Danny	voelt	frustratie	dat het binnengekomen onderdeel	weer het verkeerde blijkt, dit kost hem geld
13	Kirsten hangt wat rond met haar vrienden in het park. Ze drinken biertjes en zijn wat aangeschoten en baldadig. Een van Kirstens vrienden die meedrinkt begint opmerkingen te maken over mensen die langslopen. Eerst zachtjes, maar later steeds harder. Plotseling loopt een donkere jongen langs het groepje vrienden. Kirstens vriend roept een racistische opmerking naar de jongen en daagt hem uit.	Kirsten lacht lekker mee en doet er een schepje bovenop door de jongen ook uit te schelden en hem te bespugen.	De volgende dag gaat Kirsten naar de elektronicazaak. Ze heeft al een tijd een Ultra HD-tv op het oog en vandaag wil ze hem kopen. De tv is erg duur en Kirsten heeft er lang voor gespaard. Ze heeft de achterbank van haar auto vast naar beneden geklapt om ruimte te maken. Aangekomen bij de winkel vraagt Kirsten direct een medewerker naar de tv die ze wil.	…	Kirsten	voelt	blijdschap	wanneer in de winkel blijkt dat	de tv zelfs in de aanbieding is en ze geld overhoudt
14	Judith zit op haar fiets voor de supermarkt. Ze is met een groepje van drie vrienden en ze hangen maar wat. Terwijl ze wat dom ouwehoeren ziet Judith een hardloper aankomen. De loper is hevig bezweet en het is hem aan te zien dat hij tot het uiterste gaat. Judith en haar vrienden staan pal in het pad van de hardloper. Judith haar vrienden doen een paar stappen opzij om plaats te maken.	Judith stapt snel van haar fiets af en tilt haar fiets aan de kant zodat de loper ongehinderd door kan lopen.	Later die dag loopt Judith van een terrasje door de stad naar huis. Het is nog redelijk vroeg, maar het begint al te schemeren. De zomer loopt ten einde, maar de temperatuur is nog lekker. Judith steekt de brug over en merkt ineens dat haar portemonnee niet in haar zak zit. Ze zou zweren dat ze hem net nog had en draait zich om om te kijken of hij ergens ligt.	…	Judith	voelt	woede	als ze zich omdraait en	ziet hoe een man wegrent met haar portemonnee
15	Adriana loopt door het bos op een zondagochtend. Het is vroeg in de herfst en de bladeren beginnen net te verkleuren. Ze heeft onderweg koffie gehaald bij de enige koffiezaak die op een zondagochtend open is. Terwijl ze wandelt, neemt ze af en toe een slokje. Ze loopt graag in dit bos, het is redelijk groot en ze komt doordeweeks niet veel buiten. Halverwege haar gewone route heeft ze haar koffie op.	Adriana gooit het bekertje in de bosjes en loopt rustig verder, iemand anders raapt het vast wel weer op.	Een uur later loopt Adriana nog steeds in het bos. Ze is ondertussen een heel eind bij de auto vandaan, maar ze is nog lang niet moe. Dan komt haar voet ineens in een gat terecht en verzwikt ze haar enkel. Ze voelt een scherpe pijn en valt om omdat ze niet meer op dat been kan staan. De pijn bonst stug door in haar enkel en ze kan niet anders dan langs het pad blijven zitten.	…	Adriana	voelt	blijdschap	als er even later iemand langskomt	die haar vriendelijk naar de auto helpt
16	Kelly loopt door het park op weg naar een feestje. Het schemert al, maar het is nog redelijk licht. Het is een warme dag geweest en er liggen nog mensen op handdoeken op het gras. Kelly pakt haar telefoon om op te zoeken waar ze precies moet zijn. Dan ziet ze vanuit haar ooghoek een portemonnee liggen. Er is niemand te bekennen en Kelly pakt de portemonnee op die vol geld blijkt te zitten.	Kelly haalt alle cash eruit, 100 euro, en gooit de portemonnee weer terug op de grond, dat is mooi meegenomen.	Kelly vervolgt haar weg naar het feestje en gaat daarna nog even de kroeg in. De volgende ochtend komt ze redelijk op tijd uit bed. Gelukkig heeft ze geen kater want ze moet nog voor haar studie aan de slag vandaag. Ze zet de laptop aan en gaat dan eerst koffie maken. Als ze terug komt opent ze haar mail en ziet een mailtje van de studieadviseur.	…	Kelly	is	furieus	als ze in de email leest dat ze	niet mag herkansen en niet kan afstuderen
17	Kim stapt 's middags in de tram naar huis. Ze checkt in met haar pas en loopt door naar achter om een plaatsje te zoeken. Achter Kim stapt ook nog een vrouw in, ze loopt achter haar aan. Ze is hoogzwanger en volgt Kim verder de tram in. Kim ziet achterin nog één vrije zitplaats en stevent er op af. Voordat Kim de zitplaats bereikt begint de tram schokkend weer te rijden.	Kim aarzelt niet en stapt opzij zodat de zwangere vrouw rustig kan gaan zitten in de slingerende tram.	Eenmaal thuis neemt Kim de stapel post door. Er zit een zoveelste brief bij van de belastingdienst. Ze is al maanden bezig om haar aanslag gecorrigeerd te krijgen. De belastingdienst heeft een fout gemaakt en weigert die te herstellen. Ze hoopt dat het eindelijk is opgelost, ze heeft er genoeg van.	…	Kim	is	opgewekt	wanneer blijkt dat haar aanslag	nu eindelijk wel klopt
18	David staat op de steiger op het punt om te beginnen te schuren. Het is halverwege de ochtend en het is al flink heet. Het is de derde week van deze klus en ze zijn met het laatste stuk bezig. Hij is alleen aan het werk aan deze kant, zijn collega's zijn achter bezig. Hij ziet door het raam een vrouw achter een bureau zitten. Haar telefoon gaat en ze neemt aan en begint een heel gesprek.	David wil haar niet storen en wacht even tot ze klaar is en waarschuwt dan dat hij lawaai gaat maken.	Na de lunch klimt David weer de steiger op. Hij gaat verder met het schuurwerk, ondertussen is hij bij het laatste raam. Het werk is goed opgeschoten ondanks de hitte. David werkt gestaag door en al gauw is ook het laatste kozijn geschuurd. David zet de machine uit en terwijl hij zijn bezwete voorhoofd afveegt glipt de schuurmachine uit zijn vingers.	…	David	is	chagrijnig	als hij ziet hoe het apparaat	van de steiger valt en op de tegels kapot gaat
19	Michael gaat met zijn vrienden naar een voetbalwedstrijd. Hun club speelt uit tegen de aartsrivaal. De sfeer is gespannen, want de winnaar heeft grote kans kampioen te worden. Ze komen in een vak met medesupporters te zitten. Voor aanvang wordt er om een minuut stilte gevraagd voor een overleden speler van de tegenpartij. Het is rumoerig in het vak en ook Michaels vrienden praten en lachen.	Michael heeft weinig respect voor de tegenstander en zet luidkeels het clublied in, en de rest zingt mee.	Een week later gaat Michael op vakantie. Hij vliegt 's ochtends vroeg naar Cyprus, waar hij een week in de zon wil liggen. Op internet heeft hij een goedkoop appartementje geboekt. De foto's van het appartement waren wat onduidelijk, maar Michael kon de aanbieding niet laten gaan. Eenmaal geland op Cyprus huurt Michael een auto en rijdt naar het appartement.	…	Michael	is	verheugd	wanneer bij aankomst blijkt dat het	nog mooier is dan gedacht en het weer schitterend is
20	Wendy komt wat laat aan bij een feestje van haar broer. Het is een soort housewarming. Haar broer heeft zowel vrienden en familie als collega's uitgenodigd. Het is een flink groot feest, maar Wendy haar broer had geen zin om drie keer iets te organiseren. Wendy wordt gespot door haar broer en her en der voorgesteld. Dan stelt haar broer haar voor aan een collega die een mismaakte hand heeft.	Wendy schudt de man de hand alsof ze het niet doorheeft en groet hem vriendelijk, ze wil de man niet beledigen.	Later op de avond staat Wendy met wat familieleden te kletsen. Een aantal neven en een tante zijn ook aanwezig. Wendy heeft ze al een poos niet gezien en ze praat ze bij over haar leven. Wanneer Wendy even naar de wc gaat en weer terugkomt, treft ze iedereen lachend aan. Ze stoppen abrupt als ze er weer bij komt staan en Wendy kijkt ze vragend aan.	…	Wendy	is	kwaad	wanneer ze even later	merkt dat ze haar belachelijk maakten
21	Laura komt pas om een uur of acht uit een vergadering. Ze is moe, maar tevreden over de uitkomst. Ze besluit naar huis te lopen omdat het een mooie zomeravond is. Eenmaal uit de drukte van het centrum is het heerlijk rustig op straat. Een paar straten van haar huis ziet Laura het portier van een auto op een kier staan. Ze kijkt rond of er iemand in de buurt is, maar er is niemand.	Laura denkt na en besluit dat ze het beste gewoon het portier kan sluiten zodat niemand de auto leeg kan halen.	De volgende ochtend staat Laura vroeg op en gaat naar haar werk. Ze is nieuwsgierig naar de uitkomst van een subsidieaanvraag die ze heeft ingediend. Bij de vergadering van gisteren heeft ze geprobeerd de laatste twijfelaars over te halen. Het leek erop dat de aanvraag ingewilligd ging worden. Laura probeert gewoon te werken terwijl ze op nieuws wacht.	…	Laura	is	trots	wanneer blijkt dat haar aanvraag	is goedgekeurd en haar project kan starten
22	Leonie gaat naar de schouwburg voor een toneelvoorstelling. Het is druk, want het is een mooi stuk en er spelen veel bekende acteurs mee. Voor de kassa staat een enorme rij, waar Leonie achter aansluit. Plots hoort Leonie iemand verderop haar naam roepen. Verderop in de rij staat een vriend van Leonie naar haar te zwaaien. Hij wenkt Leonie en gebaart dat ze bij hem in de rij moet komen staan.	Leonie loopt naar voren en sluit bij haar vriend aan, een vrouw die er wat van zegt scheldt ze uit voor hoer.	Na de voorstelling loopt Leonie naar de garderobe. Ze had een jas en een paraplu meegenomen, omdat er stevige buien waren voorspeld. Bij de garderobe staat personeel dat de jassen en dergelijk ophaalt. Leonie haalt het garderobenummer tevoorschijn dat ze had gekregen. Ze geeft het nummer aan het garderobepersoneel, dat op zoek gaat naar haar spullen.	…	Leonie	voelt	blijdschap	wanneer het garderobepersoneel	haar spullen snel vindt en ze droog thuiskomt
23	Thomas zit in de kroeg met vrienden. Het is een mannenavondje en ze drinken er lustig op los. Thomas heeft vakantie en zijn vriendin is een week bij haar ouders. Hij mist haar wel, maar aan de andere kant is wat vrijheid ook wel fijn. Als iedereen besluit naar huis te gaan blijft Thomas nog even hangen. Hij raakt aan de praat met een mooie vrouw aan de bar die hem wel ziet zitten.	Thomas maakt een praatje met haar en merkt terloops op dat hij een vriendin heeft, en gaat later alleen naar huis.	De volgende dag sleept Thomas zich rond twee uur naar de supermarkt. Hij heeft niks meer in huis en ondanks een lichte kater toch honger. Wanneer hij uit de supermarkt komt zet hij de tassen even neer. Hij heeft gelijk maar grote boodschappen gedaan en het weegt heel wat. Wanneer hij bij zijn flat aankomt, ziet hij iemand vlak voor zich de deur openen.	…	Thomas	is	furieus	als de man omkijkt, even wacht en	dan de deur in zijn gezicht dicht laat vallen
24	Nina staat 's ochtends vroeg op en komt gelijk in actie. Ze gaat vandaag weer naar huis en ze wil nog winkelen voor ze vliegt. Ze heeft in het huis van vrienden van vrienden gezeten. Ze kent ze verder niet, maar ze hebben haar gratis laten logeren. Nina komt de keuken binnen en trekt de koelkast open. Het lampje springt niet aan en Nina komt er achter dat er een stop is gesprongen.	Nina besluit dat winkelen minder belangrijk is en laat een electricien komen die ze vervolgens zelf betaalt.	Later die dag rent Nina beladen met al haar bagage naar de trein. Ze springt net op tijd in de trein richting het vliegveld. De trein vertrekt op tijd maar tien minuten later staan ze ineens stil tussen de weilanden. Nina begint zich zorgen te maken dat ze te laat gaat komen. Een uur later dan gepland komt de trein eindelijk aan op Schiphol.	…	Nina	is	opgelucht	als ze aankomt op het vliegveld en	blijkt dat haar vlucht ook vertraagd is

25	Koen rijdt met de auto door de binnenstad. Het is druk op de weg en er komt een onoverzichtelijke kruising aan. Fietsers en auto's moeten hier de trambaan over vanwege werkzaamheden. Een oudere man op de fiets wankelt onzeker de trambaan op. Het is duidelijk dat de man in de war is en niet goed weet wat hij moet doen. De man komt bijna volledig tot stilstand recht voor Koen zijn auto.	Koen stapt uit om de man veilig naar de overkant te loodsen, terwijl hij de toeterende auto's tot stilte maant.	Drie straten verder slaat Koen rechstaf richting zijn appartement. Hij parkeert een straat verderop waar bijna altijd wel plaats is. Koen zet de motor af en graait zijn tas van de achterbank. Nadat Koen de auto op slot heeft gedaan zwaait hij zijn tas over zijn schouder. Bij de entree van zijn gebouw ziet hij vanuit zijn ooghoek iemand aan komen lopen.	…	Koen	is	uitzinnig	wanneer de jongen op hem afloopt	en zijn sleutels gevonden blijkt te hebben
26	Cynthia is op het werk de penningmeester van de personeelsvereniging. Het is een initiatief van en voor werknemers en ze organiseren af en toe gezellige uitjes. Daarnaast verwelkomen ze nieuwe collega's met een cadeautje. Eén keer per jaar innen ze daarvoor vrijwillig contributie van iedereen. Cynthia heeft vorige week een mailtje uitgestuurd met het verzoek te betalen. Ondertussen heeft bijna iedereen het geld overgemaakt.	Cynthia koopt zich die middag een nieuwe winterjas van het geld, er is toch niemand die haar controleert.	De week daarop moet Cynthia naar de dokter. Ze is al een poosje erg vermoeid en heeft weinig eetlust. Ze heeft daarom vorige week bij de dokter bloed laten prikken. De doktersassistente belde gisteren dat ze de volgende ochtend langs moest komen. Het is standaard procedure dit soort nieuws niet aan de telefoon te vertellen, maar ze is toch nerveus.	…	Cynthia	is	kwaad	als uiteindelijk blijkt dat	de resultaten zoek zijn en ze opnieuw moet
27	Frank loopt richting de ingang van het strandpaviljoen. Hij heeft met vrienden afgesproken, maar is aan de vroege kant. Frank loopt blootsvoets over het zand door de branding. Ter hoogte van het paviljoen begint hij aan de klim het strand op. De eerste trede van de trap naar boven is vrij hoog boven het zand. De stap is zo groot dat een jochie van een jaar of vier de trede niet op komt.	Frank hurkt bij het kind neer en helpt hem de trede op en neemt rustig de tijd om hem verder omhoog te helpen.	Frank zoekt binnen een tafel waar iedereen kan zitten en bestelt alvast wat te drinken. Een half uurtje later is iedereen aanwezig en bestellen ze wat te eten. Frank bestelt een biefstuk, medium rare met friet. Wanneer de biefstuk arriveert, blijkt hij bijna well-done te zijn. Frank roept de ober terug om het probleem uit te leggen en laat de biefstuk zien.	…	Frank	is	ziedend	als de ober reageert op een	boze manier en net doet of hij zeurt
28	Remco zit in de auto, op weg naar zijn moeder. Hij is de stad nog niet uit en het spoelt van de regen. Het regent onophoudelijk sinds gistermiddag en er hebben zich flinke plassen gevormd. Op sommige plekken staat de straat volledig blank. Er zijn weinig auto's en al helemaal weinig fietsers en voetgangers op straat. Remco stevent op een gigantische plas af en ziet iemand op de stoep lopen.	Remco geeft extra gas om een zo groot mogelijke golf water te creëren, de wandelaar wordt zeiknat gespetterd.	Eenmaal buiten de stad schiet het lekker op op de snelweg. Het blijft rustig op de weg en Remco geniet van het landschap en de rit. Hij heeft de radio hard aan staan en zingt luidkeels mee. Wanneer hij naar het dashboard kijkt om de zender bij te stellen ziet hij een lampje. Hij is vergeten te tanken en de benzine was al heel lang echt bijna op.	…	Remco	is	gefrustreerd	als hij na een paar minuten rijden	geen bezine meer heeft en stil komt te staan
29	Maarten is als enige van zijn team op werkbezoek in het buitenland. De bedoeling is om een kijkje te nemen bij een vergelijkbaar team op het hoofdkantoor. Maarten weet dat dit team op hun projecten uit is. Ze willen maar wat graag dat Maartens afdeling moet sluiten. De teamleider van het rivaliserende team wil een één op één gesprek met Maarten. Hij nodigt Maarten uit om bij hen te komen werken als hij zijn team saboteert.	Maarten denkt alleen maar aan zichzelf en stemt meteen toe, hij hoort toch liever bij het winnende team.	Later die week vliegt Maarten terug naar huis. Het is een lange vlucht en hij heeft onderweg veel om over na te denken. Als het vliegtuig eindelijk landt wacht hij ongeduldig op zijn bagage. Het is hartstikke vroeg en hij wil zijn vrouw verrassen met een ontbijt op bed. Onderweg naar huis koopt hij allerlei lekkere dingen en gaat dan snel door naar huis.	…	Maarten	voelt	razernij	als hij eenmaal thuiskomt	en zijn vrouw met ander in bed aantreft
30	Jordy loopt over het strand terug naar zijn handdoek. Onderweg komt hij langs een kraampje dat friet en dergelijke verkoopt. Het is er niet druk, het is überhaupt niet druk vandaag. Ondanks dat het een mooie dag is, is er maar een tiental mensen. Jordy loopt voorbij een meisje met een mitella dat probeert een flesje cola te openen. Het wil niet echt lukken met één hand en ze kijkt gefrustreerd.	Jordy blijft staan, lacht haar vierkant uit om haar gestuntel en loopt dan nog steeds geamuseerd door.	Jordy gaat nog een stukje zwemmen in de aanzienlijke golfslag. Daarna gaat hij liggen drogen in de zon en leest zijn boek. Hij gaat helemaal op in het verhaal en de tijd verstrijkt. Op een gegeven moment bedenkt hij zich dat de laatste bus vrij vroeg gaat op zondag. Hij checkt hoe laat het is en bedenkt zich dat hij zal moeten opschieten om de bus te halen.	…	Jordy	is	pissig	als de buschauffeur hem ziet	en gewoon doorrijdt en hem daar laat staan
31	Ruben zit op de bank met zijn laptop op schoot wanneer de bel gaat. Hij zet de laptop op de bank naast zich en staat op. Het is een pakketje voor iemand anders in het gebouw. Ruben neemt het aan en ziet op de pakbon staan dat het een geluidsinstallatie is. Ruben gaat weer verder met waar hij mee bezig was. Later die dag gaat de deurbel en het is de buurvrouw die haar pakketje komt halen.	Ruben doet net of hij geen pakje heeft en houdt stug vol tot ze weg gaat, de stereo zet hij in zijn slaapkamer.	De volgende dag is Ruben onderweg naar huis van de supermarkt. Hij heeft twee volle en zware tassen boodschappen. Bij de stoplichten zet hij de tassen naast zich neer en wacht op groen. Wanneer het groen wordt pakt hij de tassen op en haast zich naar de overkant. Bijna aan de overkant struikelt Ruben over de stoeprand en valt tegen de man voor hem aan.	…	Ruben	wordt	witheet	wanneer de man voor hem	hem een duw geeft en hij dus keihard valt
32	Dennis stapt de lift in in het ziekenhuis. De vrouw van zijn broer is gisteren bevallen en hij gaat op kraamvisite. Hij heeft een grote knuffel onder zijn arm en een bos bloemen. Hij stapt op de begane grond in en moet op de vijfde verdieping zijn. Op de derde verdieping stopt de lift en stapt een vrouw in. De vrouw is kaal en mager, ze heeft wallen onder haar ogen en een grauwe huid.	Dennis groet haar vriendelijk en maakt een praatje met haar voordat hij op de vijfde verdieping uitstapt.	Wanneer hij eenmaal zijn jongste neefje heeft bekeken gaat Dennis weer. Hij is met de bus, die stopt vlak bij hem voor de deur. Hij is binnen een half uurtje thuis en maakt wat te eten. In de keuken ziet hij op het bord zijn to-do lijstje en herinnert zich dat de tuin een wildernis is. Hij werpt een snelle blik naar buiten om te zien hoe erg het is.	…	Dennis	is	kwaad	als hij de tuin inkijkt en ziet dat	de buren hun tuinafval in zijn tuin hebben gegooid
33	Saskia is vrijwilliger bij een grote manege en vandaag hebben ze open dag. Vanuit de hele omgeving komen ouders en vooral kinderen naar de manege. Er worden rijlessen en workshops over paardenverzorging gegeven. Saskia heeft de hele ochtend workshops gegeven.'s Middags laat ze jonge kinderen ritjes maken in de bak. Wanneer Saskia zich omdraait naar het volgende kind ziet ze meteen dat het downsyndroom heeft.	Saskia doet extra haar best om het meisje een mooie tijd te geven en ze straalt van geluk op het paard.	De volgende ochtend is Saskia vroeg op haar normale werk. Ze werkt bij een groot accountantsbedrijf. Ze moet om 9:00 bij de baas komen en ze weet niet precies waarvoor. Ze heeft er slecht van geslapen vannacht terwijl ze probeerde te verzinnen waar het over zal gaan. Wanneer het eenmaal negen uur is en Saskia bij de baas zit, steekt hij gelijk van wal.	…	Saskia	is	laaiend	wanneer de baas in het gesprek	haar onterecht dingen aanrekent en ontslaat
34	Tim loopt 's middags door de supermarkt. Op zijn lijstje staat van alles om zelf een verjaardagstaart te maken. Wanneer hij alles heeft gevonden gaat hij naar de kassa. Voor hem in het gangpad loopt een oudere vrouw. Ze is duidelijk ook onderweg naar de kassa, maar ze loopt minder snel dan Tim. Het gangpad is niet zo heel breed en er komt iemand van de andere kant aanlopen.	Tim houdt netjes zijn pas in en laat de oude dame voor gaan in de rij, hij heeft tenslotte geen haast.	Later die dag is hij bezig de taart te bakken, hij staat in de oven. Hij viert vandaag zijn verjaardag en heeft iedereen eigengemaakte taart beloofd. Tim staat niet bepaald bekend om zijn culinaire hoogstandjes. Een aantal van zijn vrienden en familie reageerde dan ook nogal sceptisch. Het is nu een kwestie van eer geworden dat deze taart goed lukt.	…	Tim	is	blij	wanneer de cake uiteindelijk	perfect uit de oven komt
35	Joris loopt 's ochtend over de markt richting station. Hij heeft zijn oordopjes in en luistert naar muziek. De marktkooplui zijn in de weer met het opbouwen van hun kramen. De klinkers zijn wit uitgeslagen van de vorst van vannacht. Een man passeert voor Joris met een stapel kisten vol mandarijnen in zijn armen. De bovenste wankelt wat en even later ligt de straat bezaaid met mandarijnen.	Joris stopt en helpt de man gauw met het oprapen van de mandarijnen en maakt ondertussen een praatje.	Later die ochtend komt Joris terug van de lunch. Hij gaat weer achter zijn bureau zitten en kijkt naar zijn lijstje met taken voor vandaag. Hij is lekker opgeschoten vanochtend, maar kan nu eigenlijk niet verder. Hij wacht op bericht van een afdeling in het buitenland. Hij heeft ze gemaild of ze op willen schieten omdat hij niet verder kan zonder de info.	…	Joris	is	woest	als hij een mailtje krijgt met daarin	informatie die hij 3 maanden geleden nodig had
36	Mark rent snel terug richting het eigen doel na de gefaalde aanval. De tegenstander heeft net de bal terugveroverd. Zijn team heeft moeite met terugschakelen naar verdedigen. Over de flank krijgt de spits van de tegenstander een geweldige lange pass. Mark sprint om hem af te snijden op weg naar het doel. Mark zet een tackle in en beseft zich meteen dat hij veel te hard in heeft gezet.	Mark trekt zijn been gauw in omdat hij niemand wil verwonden, ook al kan daardoor de aanval wel doorgaan.	De bal komt bij de keeper terecht en die speelt Mark aan. Mark neemt de bal aan en begint een soloactie langs de lijn. Hij passeert twee man en een derde komt veel te laat aanrennen. Mark trekt naar binnen richting de zestien meter van de tegenstander. Zijn teamgenoten zijn nog niet mee naar voren en dus legt Mark zelf aan om uit te halen.	…	Mark	is	trots	als hij aanlegt om te schieten	en de keeper passeert en scoort
37	Myrthe krijgt haar schoonouders vanavond op bezoek en ze blijven eten. Ze wil indruk op hen maken met haar kookkunsten. Ze heeft alleen nog niks in huis en rijdt daarom naar de supermarkt. Myrthe parkeert haar auto op de parkeerplaats en loopt naar de ingang van de supermarkt. Bij de ingang staat een man die overduidelijk dakloos is. De man loopt op Myrthe af en vraagt om wat kleingeld.	Myrthe spuugt naar de man, geeft hem een duw, en sist hem toe 'kruip terug in een hoekje vieze mongool'.	Wanneer Myrthe de boodschappen heeft gekocht, rijdt ze weer naar huis. Thuis aangekomen loopt ze met haar boodschappen naar de voordeur. Myrthe kan haar voordeursleutel niet vinden. Op dat moment bedenkt ze zich dat ze zonder sleutel de deur achter zich dicht heeft gedaan. Snel loopt ze naar het huis van haar buren, die een reservesleutel hebben.	…	Myrthe	is	opgelucht	als ze bij de buren aanbelt en	die thuis blijken te zijn in ze dus haar huis in kan
38	Bram is op stap met een groepje vrienden. Ze kennen elkaar nog van hun studietijd en spreken af en toe af. Bram heeft al een aantal biertjes op en de rest heeft hem ook aardig zitten. Op een gegeven moment besluiten ze naar de volgende kroeg te gaan. Voor de ingang van de kroeg staat een groepje kerels te roken. Één van Bram zijn vrienden loopt op ze af en begint ruzie te zoeken.	Bram blijft kalm en springt er tussen, hij dwingt zijn vriend excuus te maken voordat de boel kan escaleren.	Daarna is de avond snel afgelopen en gaat Bram naar zijn fiets. Zijn fiets staat helemaal aan de andere kant van het centrum. Het is ondertussen drie uur geweest en hij wil niets liever dan slapen. Het schiet Bram ineens te binnen dat hij zijn fiets helemaal niet op slot heeft gezet. Hij graaft in zijn zakken en kan de sleutel nergens vinden.	…	Bram	is	gelukkig	als hij aankomt waar hij zijn fiets had staan	en ziet dat deze er gewoon nog staat
39	Kevin loopt 's avonds naar het huis van zijn ouders. Hij is bij een vriend geweest die hij niet had gezien sinds hij is verhuisd. Het is schemerig en het is een eind lopen, maar dat vindt Kevin niet erg. Het is fijn om weer door bekende straten te slenteren. Verderop ziet Kevin een gedaante op vier poten uit de bosjes komen. Het is een gewonde hond die zacht jankt en naar hem toe loopt.	Kevin belt gauw de dierenambulance en aait het beestje en stelt hem gerust terwijl hij op de ambulance wacht.	Eenmaal thuis na het incident met de hond gaat Kevin direct naar bed. De volgende dag staat hij vroeg op om nog wat mensen op te zoeken. Hij leent de auto van zijn ouders omdat sommigen buiten de stad wonen. Halverwege, midden tussen de weilanden, houdt de motor er mee op. Na 20 minuten wachten langs de weg komt er eindelijk een andere auto aan.	…	Kevin	is	uitgelaten	wanneer de andere automobilist	stopt en zijn auto kan repareren
40	Anouk zit alvast in de vergaderruimte waar ze straks overleg heeft. Ze neemt de documenten die ze heeft voorbereid nog eens door. In de excel sheets van de begroting spot ze ineens een grove fout. Deze fout betekent dat het project veel meer gaat kosten dan ze had gezegd. Anouk heeft weinig zin om dit op te moeten biechten. Voordat ze echt na heeft kunnen denken komen haar bazen binnen.	Anouk besluit de schuld te geven aan een medewerker onder haar die waarschijnlijk toch ontslagen gaat worden.	De vergadering verloopt verder prima en Anouk gaat door met haar dag. Tegen een uur of acht is ze thuis en haalt de post op. Eenmaal binnen ziet ze een brief van het elektriciteitsbedrijf. Het is de jaarafrekening en dat was vorig jaar een dure grap. Anouk heeft toen het voorschot bij laten stellen en hopelijk hoeft ze nu niet bij te betalen.	…	Anouk	is	driftig	als uit de brief blijkt dat ze	wederom een groot bedrag moet bijbetalen
41	Jesse komt 's middags rond half vier aanrijden bij de school. Hij parkeert in de rij wachtende ouders. Er zijn nog geen kinderen op het plein en hij wacht rustig. Hij is zo verzonken in gedachten dat hij niet merkt dat de kinderen naar buiten komen. Hij is dan ook verrast wanneer zijn dochtertje plots achterin klimt. Ze heeft een tekening gemaakt die ze vol trots laat zien en aan hem wil geven.	Jesse wijst haar erop dat ze nog steeds slordig en buiten de lijntjes kleurt en dat ze beter haar best moet doen.	Later die avond als zijn dochtertje al op bed ligt kijkt Jesse voetbal. Hij heeft geld gezet op de uitkomst van deze wedstrijd. Het was een flink bedrag en hij wil graag winnen, want hij kon het geld eigenlijk niet missen. Vlak voor tijd staat het 1-1 en hij heeft geld gezet op 2-1 winst. Hij kijkt gespannen toe hoe zijn team een laatste aanval inzet.	…	Jesse	is	woest	wanneer vlak voor het einde	de tegenpartij scoort en hij zijn geld kwijt is
42	Inge zit 's middags in de trein op weg naar Groningen. Het is ontzettend rustig in de trein sinds Zwolle. Er zitten naast Inge maar vier mensen in de hele coupé. Er zit een man in de vierzits aan de overzijde van het gangpad een boek te lezen. Wanneer de trein weer stopt staat de man op om uit te stappen. Ineens ziet Inge dat de man een tas onder de bank heeft laten staan.	Inge springt gauw overeind en weet de man nog net voordat de deuren sluiten zijn tas te overhandigen.	Eenmaal in Groningen aangekomen pakt Inge de bus richting Oranjewijk. Ze logeert bij een vriendin en ze gaan naar een concert in de Oosterpoort vandaag. De bus doet er iets langer over dan normaal, maar Inge heeft geen haast. Ze stapt uit en loopt de laatste vijf minuten naar haar huis. Vlak voor ze aanbelt, gaat haar telefoon en ze neemt op.	…	Inge	voelt	vreugde	wanneer ze te horen krijgt	dat haar bod op een huis is geaccepteerd
43	Julia zit op haar plek achter de receptie, de telefoon staat roodgloeiend. Ze handelt het ene na het andere telefoontje af. Voordat Julia het weet is de ochtend alweer voorbij. Na de lunch is het iets minder druk. Dan gaat de telefoon weer, het is iemand voor de man van accounting. De accountant heeft de laatste tijd steken laten vallen en ook nu belt er iemand met een probleem.	Julia geeft de boodschap lekker niet door en geniet wanneer de man daardoor in problemen komt en ontslagen wordt.	Later die week is Julia aan het winkelen. Ze zoekt een specifiek computerspelletje voor de verjaardag van haar broertje. Ze heeft hem beloofd dat hij deze morgen voor zijn verjaardag krijgt. Julia heeft echter weinig tijd gehad om te winkelen de laatste tijd. Eindelijk vind ze één kopie van het spelletje, maar een andere klant ziet hem tegelijkertijd.	…	Julia	is	geïrriteerd	als de andere klant het spelletje	snel afrekent en haar compleet negeert
44	Larissa is lerares op een basisschool. Ze geeft les aan de bovenbouw, groep vijf en zes. Op een woensdagochtend heeft ze pleinwacht tijdens de pauze. Ze loopt grote rondes over het plein en houdt alles goed in de gaten. Af en toe spreekt ze een leerling aan, maar verder is het rustig. Dan ziet ze een stel leerlingen uit groep 8 aan de rand van het plein rommel staan gooien naar een vrouw die langsloopt.	Larissa wil ze tot de orde roepen, maar ziet dan dat het een moslimvrouw is en laat ze lekker hun gang gaan.	Later die week gaat Larissa naar de bank voor een afspraak. Ze wil haar hypotheek oversluiten en snapt niet veel van financiën, dus wil ze persoonlijk advies. Ze loopt de bank binnen en gaat naar de balie om zich te melden. De receptioniste zegt haar plaats te nemen, ze komen haar zo halen. Larissa gaat zitten en leest een tijdschrift terwijl ze rustig wacht.	…	Larissa	is	gerustgesteld	als ze twee uur later naar buiten loopt	met een nieuwe en zeer gunstige hypotheek
45	Hendrik zit 's ochtends al vroeg op het kantoor. Hij is druk bezig met het tentamen schrijven dat zijn studenten volgende week moeten maken. Terwijl hij daar mee bezig is wordt er op de deur geklopt. Het is één van de studentes uit zijn werkgroep, een knappe verschijning. Het is al gauw duidelijk dat ze probeert Hendrik te verleiden om haar het tentamen in te laten zien. Ze zinspeelt op hoe dankbaar ze zou zijn.	Hendrik maakt misbruik van de situatie en belooft haar het tentamen als ze vanavond bij hem thuis komt.	Later die dag moet Hendrik college geven in een gebouw buiten de binnenstad. Hij pakt de fiets daarheen en trapt stevig door. Op een gegeven moment fietst hij op een drukke kruising af en stopt hij voor het licht. Even later springt het op groen en Hendrik steekt over. Halverwege de kruising vliegt de ketting er ineens af en staat hij midden op de kruising stil.	…	Hendrik	is	dankbaar	als de auto's die nu groen hebben	rustig wachten en hem vriendelijk de tijd geven
46	Eva loopt snel over het plein richting het station. Ze gaat vandaag naar Haarlem voor een sollicitatiegesprek. Ze heeft zich goed voorbereid en is ruim op tijd van huis gegaan. Vlakbij de ingang naar de stationshal staat een zwerver te bedelen. Wanneer Eva langsloopt, houdt hij een papieren bekertje omhoog met wat kleingeld erin. De man kijkt hoopvol hoe Eva in haar broekzak graaft.	Eva vist wat muntgeld op en gooit dit in het bekertje van de zwerver, ze geeft altijd wat ze kan missen.	Ze loopt snel door en kijkt op het bord welk spoor ze moet hebben. Ze ziet dat er over 2 minuten een trein gaat en zet het op een rennen. Als ze deze trein kan halen, dan komt ze sowieso op tijd. Het is druk in de tunnel naar de sporen en Eva moet steeds uitwijken. Eindelijk bereikt ze de trap en sprint met drie treden per keer naar boven.	…	Eva	is	dankbaar	als de conducteur haar ziet rennen	en vriendelijk even op haar wacht
47	Wouter werkt op de marketingafdeling van een groot bedrijf. Op dezelfde afdeling werkt zijn beste vriend. Ze werken al jaren heel veel samen en ze zijn een hecht team. Op een ochtend wordt Wouters vriend naar het kantoor van de baas geroepen. Wouter weet niet waar het over gaat, maar hij is wel nieuwsgierig. Later die dag hoort Wouter dat zijn vriend een promotie heeft gekregen en hij niet.	Wouter besluit de vriendschap te beëindigen en verspreidt een roddel dat hij geld zou hebben gestolen.	Later die week heeft Wouter een zeldzame vrije dag. Hij besluit de meubelzaken bij langs te gaan voor nieuwe meubels. Bij IKEA ziet hij een leuke garderobekast, die perfect past in zijn slaapkamer. Wouter aarzelt geen moment en koopt de kast meteen en gaat weer richting huis. Wouter begint thuis meteen enthousiast het bouwpakket in elkaar te zetten.	…	Wouter	is	tevreden	als bij het monteren blijkt	dat de kast perfect past waar hij hem wilde
48	Elise zit in de bus op weg naar de film. Ze heeft haar oordopjes in en zoekt de filmtijden nog eens op. De bus is halverwege en ze sms't haar vrienden dat ze eraan komt. Elise verandert de muziek naar iets stevigers en zet het harder. Er zitten weinig mensen in de bus behalve twee oudere dames voor haar. Eén van hen draait zich om om te zeggen dat haar muziek wel heel hard staat.	Elise verontschuldigt zich en zet haar muziek meteen zachter, vervolgens wenst ze de dames nog een fijne dag.	Twee haltes verder stapt Elise uit en loopt richting bioscoop. Bij de ingang ontmoet ze haar vrienden en ze gaan gauw naar de zaal. Na de film gaan ze nog even gezellig wat drinken. Elise bestelt een biertje, maar bedenkt zich later en loopt naar de bar om iets anders te bestellen. Ze zegt tegen de serveerster dat ze toch liever een witte wijn heeft.	…	Elise	wordt	chagrijnig	als de serveerster reageert	op een norse manier en haar boos aankijkt
49	Tom loopt naar huis van de supermarkt aan de overkant van de straat. Hij heeft één tas met boodschappen, maar die weegt niet zoveel. Met zijn vrije hand opent hij de zware portiekdeur van de flat. Achter hem loopt een vrouw met twee tassen, ze heeft moeite met het gewicht. Tom herkent haar en weet dat ze ook de flat in moet. Hij stopt even in de deuropening en denkt na wat te doen.	Hij wacht rustig tot ze er is en houdt de deur vriendelijk voor haar open, wat ze zichtbaar op prijs stelt.	Later die dag wil Tom een afspraak maken bij de dokter. Hij zoekt het nummer op en pakt de telefoon. Hij wil al een hele tijd een afspraak maken vanwege een zere knie. Tom is echter zo druk dat hij vaak geen zin heeft om te bellen, het wachten duurt altijd zo lang. Hij moet eigenlijk al weer de deur uit, maar wil toch even snel bellen.	…	Tom	is	verrast	wanneer de telefoon over gaat	en hij gelijk de assistente krijgt

50	Niels is 's ochtends vroeg als eerste op kantoor. Het is verder muisstil op de afdeling. Na een half uurtje heeft Niels al flink wat werk verzet, dit is zijn favoriete deel van de dag. Hij legt de laatste hand aan een document en streept hem van zijn lijstje. Vervolgens gaat hij koffie halen en komt de eerste collega tegen. Niels kent haar vaag en weet toevallig dat ze sinds kort op dieet is.	Niels zegt goedemorgen en merkt op dat het dieet niet lijkt te helpen, misschien minder suiker in de koffie?	Niels werkt rustig door tot de lunch en daarna heeft hij een vergadering. Zijn team presenteert vandaag de resultaten van een project. Het project is een enorm succes geworden voor het bedrijf. Niels presenteert vandaag zelf niet, maar hij weet dat hij een integraal onderdeel van dit succes is. De belangrijkste ideeën voor het nieuwe programma kwamen van hem.	…	Niels	voelt	razernij	wanneer zijn manager in de meeting	liegt en alle eer voorzichzelf opstrijkt
51	Mike is op weg naar de sportschool op een doordeweekse avond. Het begint net wat te schemeren en het regent gestaag. Hij steekt over bij de stoplichten en gaat de hoek om. Aan het eind van de straat ziet hij mensen in en uit lopen bij de sportschool. Aan de overkant van de straat fietst een oudere vrouw de andere kant op. Mike ziet hoe ze plots begint te slingeren en met fiets en al omvalt.	Mike bedenkt zich niet en rent er naar toe en helpt haar overeind, ze mankeert niks maar is blij met de hulp.	Eenmaal in de sportschool kleedt Mike zich om. Dan loopt hij snel naar de loopbanden, waar het altijd druk is. De loopbanden staan boven en halverwege de trap ziet hij dat zijn veter los zit. Hij bukt zich om deze te strikken en haast zich dan verder naar boven. Hij ziet dat er nog eentje vrij is, maar een andere man loopt ook op de band af.	…	Mike	is	dankbaar	wanneer de man die ook wil rennen	hem vriendelijk voor laat gaan
52	Rianne loopt door het bos met de hond. Het is zaterdagmiddag en er schijnt een waterig zonnetje. Terwijl de hond druk bezig is met snuffelen belt Rianne met een vriendin. Ze hebben elkaar al een poosje niet gesproken en praten bij. Rianne doet een verhaal uit de doeken over een dronken avond op zakenreis. Rianne merkt ineens dat ze langs het kerkhof loopt en dat een groep rouwers bij een nieuw graf staat.	Rianne gaat gelijk zachter praten en hangt snel op, ze knikt de rouwers toe bij wijze van groet en excuus.	Later die dag bedenkt Rianne zich dat ze wel zin heeft om wat te doen die avond. Ze is al een hele poos niet meer op stap geweest. Ze stuurt een berichtje op de groepsapp van een groep vrienden van het volleyballen. Ondertussen gaat Rianne alvast iets te eten klaar maken. Tijdens het eten checkt ze haar telefoon en leest de reacties van iedereen.	…	Rianne	wordt	woest	als ze leest dat iedereen	zegt niet te kunnen terwijl ze weet dat ze wel uitgaan
53	Bart stapt uit de auto en loopt richting het uitvaartcentrum. Hij is hier met een paar andere mensen van het werk voor de uitvaart van een collega. Hij kende de man niet goed, maar sommigen van zijn collega's waren wel close. Hij zoekt samen met zijn collega's een plekje achterin. De dienst begint en na het eerste nummer spreekt zijn vrouw. Een collega van Bart begint zachtjes te huilen.	Bart slaat een arm om haar heen om haar te troosten en haar steun te bieden, wat ze duidelijk op prijs stelt.	Een week later heeft Bart een belangrijke afspraak met zijn manager.Het is een vervroegd kwartaalgesprek om zijn functioneren te evalueren. Bart heeft geen redenen om te denken dat zijn manager iets te klagen zou hebben. Maar zo'n gesprek is toch altijd spannend en stressvol. Als ze eenmaal zitten en de beleefdheden hebben gehad, steekt zijn manager van wal.	…	Bart	is	opgelucht	wanneer zijn manager hem vertelt	dat hij een promotie en opslag krijgt
54	Sander werkt bij een groot softwarebedrijf. De directeur van het bedrijf is kort geleden met pensioen gegaan. Sander zit in de sollicitatiecommissie om een geschikte nieuwe directeur te vinden. De eerste brieven en cv's van kandidaten zijn binnengekomen. Sander print de documenten uit en maakt een overzicht van alle kandidaten. Het valt hem op dat er veel vrouwelijke kandidaten zijn.	Sander denkt dat het goed zou zijn om een vrouw aan het roer te hebben, en hij zet de vrouwelijke kandidaten gewoon op de lijst.	Wanneer Sander klaar is met werken rijdt hij het half uurtje naar huis. Eenmaal thuis besluit hij een eindje te gaan hardlopen. Hij trekt zijn hardloopkleding aan en loopt zijn huis uit. Na wat warming-up en rekken en strekken gaat hij op pad. Na een half uurtje stevig lopen komt Sander bij een smalle straat waar een groepje jongeren de weg blokkeert.	…	Sander	wordt	boos	als het groepje jongeren	expres de weg blijft blokkeren en lacht
55	Tamara is eigenaar van een koffiezaak. Ze heeft vandaag een aantal sollicitatiegesprekken. Het gaat voornamelijk om tijdelijke krachten die in de vakantieperiode bijspringen. Toch moet ze ook die zorgvuldig selecteren. De eerste drie kandidaten zijn niet super geschikt op het eerste gezicht. Tamara is verbaasd als de volgende een enigszins ruig uitziende Turkse jongen blijkt te zijn.	Tamara interviewt de jongen en uiteindelijk blijkt hij de meest geschikte kandidaat en krijgt de baan.	Aan het einde van de dag is Tamara met het schoonmaakwerk bezig. De balie is schoon en de vloer is geveegd, maar nog niet gedweild. Er zit nog één klant in de hoek en ze zijn officieel nog open. Tamara wacht daarom met dweilen en vult de suikerpotten bij. Tamara loopt met een zwaar dienblad vol suikerpotten als de laatste klant vol tegen haar op botst.	…	Tamara	wordt	furieus	als de klant die haar aanstootte	geen sorry zegt en zelfs geamuseerd omkijkt
56	Thijs stapt snel op de fiets om nog iets te halen in de stad. Hij fietst de straat uit en richting centrum. Hij zet zijn fiets op slot tegen een paal bij het gemeentehuis. Hij hoeft maar één ding te halen en neemt verder niet de tijd om rond te kijken. Hij slalomt door de mensenmassa heen en schiet lekker op. Ineens staat hij vlak voor een gehandicapte jonge vrouw met een rollator.	Thijs duwt haar zowat omver wanneer hij zich langs haar wurmt, 'aan de kant, lelijke mongool' bijt hij haar toe.	Thijs vervolgt zijn weg naar de winkel en koopt snel wat hij nodig heeft. Daarna baant hij zich zo snel mogelijk een weg terug door de binnenstad. Halverwege voelt hij ineens een zachte aanraking. Hij voelt in zijn zak, merkt dat zijn portemonnee weg is en ziet ineens iemand snel weglopen. Thijs schreeuwt dat hij bestolen is en de dief zet het op een hollen.	…	Thijs	is	ziedend	als hij ziet hoe de omstanders	domweg opzij gaan en de dief ontsnapt
57	Jeroen is twee weken op vakantie in Italië. Hij heeft een groot deel van de eerste week aan het strand doorgebracht. De tweede week wil hij iets meer cultuur opsnuiven. Vandaag heeft hij de trein genomen naar een oud stadje vlakbij. Er schijnt een heel mooie en oude kerk te staan. Wanneer hij in zijn hemd de kerk wil binnenlopen, wijst iemand hem op een bord met kledingvoorschriften.	Jeroen verontschuldigt zich uitvoerig en haalt een shirt met lange mouwen uit zijn tas en trekt dit snel aan.	Jeroen gaat op het terras zitten bij het enige restaurant dat hij heeft gezien. Hij spreekt geen Italiaans en hoopt dat ze Engels spreken. Hij probeert uit te puzzelen wat er op de kaart staat, hij is vegetariër. Het is een warme dag en Jeroen is blij met de schaduw terwijl hij op de ober wacht. Als de ober langskomt, vraagt Jeroen of hij Engels spreekt.	…	Jeroen	is	witheet	als de ober vervolgens	zonder wat te zeggen omdraait en hem negeert
58	Patrick heeft sinds een jaar een eigen bedrijf. Het bedrijf is zo gegroeid dat Patrick een secretaresse nodig heeft. Hij heeft heel veel reacties gehad. Eén sollicitante steekt boven alle anderen uit door haar ervaring en opleiding. Patrick nodigt haar uit en is tijdens het gesprek erg enthousiast. Zijn zakenpartner wijst hem er later op dat ze wel wat ouder is en andere kandidaten er leuker uitzagen.	Patrick denkt nog eens na en besluit inderdaad dat hij liever een lekker ding heeft en neemt een ander aan.	Later die dag werkt Patrick aan een verslag voor een klant van zijn bedrijf. Het is een eindrapportage van alle werkzaamheden. Het is een groot document geworden dat Patrick veel kostbare tijd heeft gekost. Wanneer Patrick de laatste punt neerzet, valt ineens de stroom uit. Patrick bedenkt zich dat hij tussendoor niet heeft gesaved en start de computer weer op.	…	Patrick	is	opgelucht	wanneer na het opnieuw opstarten blijkt	dat er een automatische back-up beschikbaar is
59	Anna staat aan de kassa bij de supermarkt. Terwijl de kassière de laatste boodschappen scant, pakt ze zoveel mogelijk in. Nadat ze het laatste artikel heeft gescand noemt ze het bedrag. Anna betaalt met 50 euro en de kassière geeft haar wisselgeld terug. Anna ziet meteen dat ze haar veel te veel wisselgeld geeft. Ze weet dat kassières flink problemen kunnen krijgen bij een kasverschil.	Anna wijst haar er dan ook vriendelijk op dat ze een fout heeft gemaakt, ze wil niet dat ze ontslagen wordt.	Na de supermarkt gaat ze ook nog even naar de boekwinkel. Ze moet echter opschieten want haar parkeerkaartje verloopt zo. Bij de boekwinkel kan ze niet gelijk vinden wat ze zoekt. Ze vraagt het de eigenaar en die helpt haar het boek te vinden. Anna rekent af en loopt, zo snel als ze kan met alle tassen, naar de parkeerplaats, ze is nu echt te laat.	…	Anna	wordt	vrolijk	als de parkeerwachter haar ziet	en besluit de boete te verscheuren
60	Nienke loopt door het park op een zondagochtend. Ze is op weg naar de winkel om brood te halen. In de verte ziet ze een oud mannetje met een rollator lopen. Nienke loopt rustig door, maar heeft de man uiteraard al gauw bijna ingehaald. De man stopt even en graaft een zakdoek uit zijn broekzak. Nienke ziet hoe tegelijk met de zakdoek ook een pasje op de grond valt, maar de man merkt niks.	Nienke roept de man snel na dat hij iets verliest en raapt het voor hem op zodat de man niet hoeft te bukken.	Later die week loopt Nienke met een laptop onder de arm. Ze is onderweg naar de winkel waar ze hem de dag daarvoor heeft gekocht. Toen ze hem gisteravond probeerde te installeren bleek hij het niet te doen. Ze is helaas het bonnetje kwijt, maar hoopt dat ze haar nog herkennen. De man in de winkel herkent haar inderdaad en vraagt om het bonnetje.	…	Nienke	is	gepikeerd	als de man vervolgens zegt dat	ze zonder bonnetje naar haar geld kan fluiten
61	Marloes is alweer een tijdje vrijgezel. Sinds kort is er bij Marloes op het werk een jongen die verliefd is op Marloes. Marloes weet dit, maar heeft absoluut geen gevoelens voor hem. Toch vindt Marloes de aandacht wel leuk en ze flirt er lustig op los. Na een avond uit met collega's zijn Marloes en de jongen alleen overgebleven. De jongen vraagt Marloes ineens blozend om met hem mee naar huis te gaan.	Marloes denkt even na en gaat lekker met hem mee naar huis en besluit hem de volgende ochtend te dumpen.	Later die week gaat Marloes naar de boekhandel. Het nieuwste deel van Marloes haar favoriete boekenserie is namelijk uit. Bovendien heeft de boekhandel vandaag een actie: de eerste 100 klanten krijgen het boek gratis. De winkel staat op het punt open te gaan en er staat al een grote menigte voor de deur. Marloes sluit achter aan en schat haar kansen in.	…	Marloes	is	dolblij	wanneer ze na een uur wachten	de 100ste klant is en het boek gratis krijgt
62	Tessa loopt na de film naar de tramhalte twee straten verderop. Onderweg komt ze langs de Febo en ze beseft ineens dat ze honger heeft. Ze trekt een kroket uit de muur en loopt verder. Aan het einde van de straat slaat ze rechtsaf naar het plein. Ze steekt het plein schuin over naar de halte. Op het plein passeert ze twee mannen die hand in hand lopen en heel gelukkig lijken.	Tessa gooit achteloos het laatste stukje kroket naar het stel, raakt de ene vol en roept 'vieze homo's'.	Een korte tramrit later is Tessa bijna thuis. Ze neemt de lift naar haar appartement op de vijfde etage. In de lift ziet ze dat één van haar veters los is en ze bukt om die te strikken. Als ze de lift uitkomt en de galerij op loopt ziet ze haar voordeur open staan. Ze herinnert zich in een flits dat ze de deur zelf niet goed dicht heeft gedaan.	…	Tessa	is	laaiend	als ze naar binnen rent en ziet dat	alles overhoop ligt en dure dingen zijn gejat
63	Jasper fietst 's avonds na een etentje bij vrienden naar huis. Op een gegeven moment nadert Jasper een kruispunt. Van links komt een meisje ook op het kruispunt af, ze is zichtbaar gehaast. Jasper heeft voorrang en steekt dus het kruispunt over. Het meisje ziet Jasper niet aankomen en rijdt ook door. Ze schrikt wanneer ze pas op het laatste moment Jasper ziet en valt met haar fiets op straat.	Jasper fietst door en roept in het voorbijgaan dat ze dan maar de verkeersregels had moeten leren, domme trut.	Jasper fietst verder en ziet in de verte een kruispunt met verkeerslichten. Hij stopt wanneer hij er aankomt, omdat het licht nog steeds op rood staat. Het licht blijft maar op rood staan, en Jasper wordt ongeduldig. Er komt niks aan en het kruispunt lijkt verlaten. Hij rijdt door het rode licht, maar ziet plots aan de overkant een politieagent staan.	…	Jasper	is	opgelucht	wanneer hij op de agent affietst	en deze knipoogt en zegt dat hij door mag rijden
64	Claudia heeft boodschappen gedaan en loopt naar de auto. Ze laadt haar boodschappen in en stapt in. Claudia start de auto, zet de radio aan en geeft gas. Claudia heeft echter niet door dat de auto in zijn achteruit staat. De auto schiet naar achteren en knalt tegen de auto die erachter geparkeerd staat. Claudia stapt uit en ziet dat de auto achter haar erg beschadigd is, maar haar eigen auto niet	Claudia kijkt om zich heen, ziet dat gelukkig niemand het incident gezien heeft, stapt in en rijdt snel weg.	Die avond staat Claudia bij bioscoop Tuschinski in Amsterdam. Er is een grote internationale filmpremière met veel beroemde acteurs. Claudia hoopt bij de rode loper een glimp van de sterren op te vangen. Tot de aanwezige sterren behoort ook Johnny Depp, een acteur die Claudia erg bewondert. Op een gegeven moment ziet Claudia hem op de rode loper verschijnen.	…	Claudia	is	uitzinnig	als Johnny Depp vervolgens	rustig de tijd neemt om met haar te kletsen

Experimental Stimulus List 2

Number	Introduction	Character Manipulation	Continuation	Transition	Name	Verb	Affective State Adjective	Neutral Segment	Affect Reason
1	Claudia heeft boodschappen gedaan en loopt naar de auto. Ze laadt haar boodschappen in en stapt in. Claudia start de auto, zet de radio aan en geeft gas. Claudia heeft echter niet door dat de auto in zijn achteruit staat. De auto schiet naar achteren en knalt tegen de auto die erachter geparkeerd staat. Claudia stapt uit en ziet dat de auto achter haar erg beschadigd is, maar haar eigen auto niet.	Claudia kijkt om zich heen, ziet dat gelukkig niemand het incident gezien heeft, stapt in en rijdt snel weg.	Die avond staat Claudia bij bioscoop Tuschinski in Amsterdam. Er is een grote internationale filmpremière met veel beroemde acteurs. Claudia hoopt bij de rode loper een glimp van de sterren op te vangen. Tot de aanwezige sterren behoort ook Johnny Depp, een acteur die Claudia erg bewondert. Op een gegeven moment ziet Claudia hem op de rode loper verschijnen.	…	Claudia	is	teleurgesteld	als Johnny Depp vervolgens	haar nors negeert en ze zelfs geen handtekening krijgt
2	Jasper fietst 's avonds na een etentje bij vrienden naar huis. Op een gegeven moment nadert Jasper een kruispunt. Van links komt een meisje ook op het kruispunt af, ze is zichtbaar gehaast. Jasper heeft voorrang en steekt dus het kruispunt over. Het meisje ziet Jasper niet aankomen en rijdt ook door. Ze schrikt wanneer ze pas op het laatste moment Jasper ziet en valt met haar fiets op straat.	Jasper fietst door en roept in het voorbijgaan dat ze dan maar de verkeersregels had moeten leren, domme trut.	Jasper fietst verder en ziet in de verte een kruispunt met verkeerslichten. Hij stopt wanneer hij er aankomt, omdat het licht nog steeds op rood staat. Het licht blijft maar op rood staan, en Jasper wordt ongeduldig. Er komt niks aan en het kruispunt lijkt verlaten. Hij rijdt door het rode licht, maar ziet plots aan de overkant een politieagent staan.	…	Jasper	is	chagrijnig	wanneer hij op de agent affietst	en deze hem toch een fikse boete geeft
3	Tessa loopt na de film naar de tramhalte twee straten verderop. Onderweg komt ze langs de Febo en ze beseft ineens dat ze honger heeft. Ze trekt een kroket uit de muur en loopt verder. Aan het einde van de straat slaat ze rechtsaf naar het plein. Ze steekt het plein schuin over naar de halte. Op het plein passeert ze twee mannen die hand in hand lopen en heel gelukkig lijken.	Tessa gooit achteloos het laatste stukje kroket naar het stel, raakt de ene vol en roept 'vieze homo's'.	Een korte tramrit later is Tessa bijna thuis. Ze neemt de lift naar haar appartement op de vijfde etage. In de lift ziet ze dat één van haar veters los is en ze bukt om die te strikken. Als ze de lift uitkomt en de galerij op loopt ziet ze haar voordeur open staan. Ze herinnert zich in een flits dat ze de deur zelf niet goed dicht heeft gedaan.	…	Tessa	is	uitgelaten	als ze naar binnen rent en ziet dat	er niks weg is, wat een geluk!
4	Marloes is alweer een tijdje vrijgezel. Sinds kort is er bij Marloes op het werk een jongen die verliefd is op Marloes. Marloes weet dit, maar heeft absoluut geen gevoelens voor hem. Toch vindt Marloes de aandacht wel leuk en ze flirt er lustig op los. Na een avond uit met collega's zijn Marloes en de jongen alleen overgebleven. De jongen vraagt Marloes ineens blozend om met hem mee naar huis te gaan.	Marloes denkt even na en gaat lekker met hem mee naar huis en besluit hem de volgende ochtend te dumpen.	Later die week gaat Marloes naar de boekhandel. Het nieuwste deel van Marloes haar favoriete boekenserie is namelijk uit. Bovendien heeft de boekhandel vandaag een actie: de eerste 100 klanten krijgen het boek gratis. De winkel staat op het punt open te gaan en er staat al een grote menigte voor de deur Marloes sluit achter aan en schat haar kansen in.	…	Marloes	is	verbolgen	wanneer ze na een uur wachten	de 101ste klant is en de volle prijs moet betalen
5	Nienke loopt door het park op een zondagochtend. Ze is op weg naar de winkel om brood te halen. In de verte ziet ze een oud mannetje met een rollator lopen. Nienke loopt rustig door, maar heeft de man uiteraard al gauw bijna ingehaald. De man stopt even en graaft een zakdoek uit zijn broekzak. Nienke ziet hoe tegelijk met de zakdoek ook een pasje op de grond valt, maar de man merkt niks.	Nienke roept de man snel na dat hij iets verliest en raapt het voor hem op zodat de man niet hoeft te bukken.	Later die week loopt Nienke met een laptop onder de arm. Ze is onderweg naar de winkel waar ze hem de dag daarvoor heeft gekocht. Toen ze hem gisteravond probeerde te installeren bleek hij het niet te doen. Ze is helaas het bonnetje kwijt, maar hoopt dat ze haar nog herkennen. De man in de winkel herkent haar inderdaad en vraagt om het bonnetje.	…	Nienke	is	gelukkig	als de man vervolgens zegt dat	ze ook zonder bonnetje haar geld terugkrijgt
6	Anna staat aan de kassa bij de supermarkt. Terwijl de kassière de laatste boodschappen scant, pakt ze zoveel mogelijk in. Nadat ze het laatste artikel heeft gescand noemt ze het bedrag. Anna betaalt met 50 euro en de kassière geeft haar wisselgeld terug. Anna ziet meteen dat ze haar veel te veel wisselgeld geeft. Ze weet dat kassières flink problemen kunnen krijgen bij een kasverschil.	Anna wijst haar er dan ook vriendelijk op dat ze een fout heeft gemaakt, ze wil niet dat ze ontslagen wordt.	Na de supermarkt gaat ze ook nog even naar de boekwinkel. Ze moet echter opschieten want haar parkeerkaartje verloopt zo. Bij de boekwinkel kan ze niet gelijk vinden wat ze zoekt. Ze vraagt het de eigenaar en die helpt haar het boek te vinden. Anna rekent af en loopt, zo snel als ze kan met alle tassen, naar de parkeerplaats, ze is nu echt te laat.	…	Anna	wordt	kwaad	als de parkeerwachter haar ziet	en toch alsnog de boete uitschrijft
7	Patrick heeft sinds een jaar een eigen bedrijf. Het bedrijf is zo gegroeid dat Patrick een secretaresse nodig heeft. Hij heeft heel veel reacties gehad. Eén sollicitante steekt boven alle anderen uit door haar ervaring en opleiding. Patrick nodigt haar uit en is tijdens het gesprek erg enthousiast. Zijn zakenpartner wijst hem er later op dat ze wel wat ouder is en andere kandidaten er leuker uitzagen.	Patrick denkt nog eens na en besluit inderdaad dat hij liever een lekker ding heeft en neemt een ander aan.	Later die dag werkt Patrick aan een verslag voor een klant van zijn bedrijf. Het is een eindrapportage van alle werkzaamheden. Het is een groot document geworden dat Patrick veel kostbare tijd heeft gekost. Wanneer Patrick de laatste punt neerzet, valt ineens de stroom uit. Patrick bedenkt zich dat hij tussendoor niet heeft gesaved en start de computer weer op.	…	Patrick	is	ziedend	wanneer het opnieuw opstarten blijkt	dat het document verloren is en het opnieuw moet
8	Jeroen is twee weken op vakantie in Italië. Hij heeft een groot deel van de eerste week aan het strand doorgebracht. De tweede week wil hij iets meer cultuur opsnuiven. Vandaag heeft hij de trein genomen naar een oud stadje vlakbij. Er schijnt een heel mooie en oude kerk te staan. Wanneer hij in zijn hemd de kerk wil binnenlopen, wijst iemand hem op een bord met kledingvoorschriften.	Jeroen verontschuldigt zich uitvoerig en haalt een shirt met lange mouwen uit zijn tas en trekt dit snel aan.	Jeroen gaat op het terras zitten bij het enige restaurant dat hij heeft gezien. Hij spreekt geen Italiaans en hoopt dat ze Engels spreken. Hij probeert uit te puzzelen wat er op de kaart staat, hij is vegetariër. Het is een warme dag en Jeroen is blij met de schaduw terwijl hij op de ober wacht. Als de ober langskomt, vraagt Jeroen of hij Engels spreekt.	…	Jeroen	is	vergenoegd	als de ober vervolgens	geduldig zijn best doet om Engels te spreken
9	Thijs stapt snel op de fiets om nog iets te halen in de stad. Hij fietst de straat uit en richting centrum. Hij zet zijn fiets op slot tegen een paal bij het gemeentehuis. Hij hoeft maar één ding te halen en neemt verder niet de tijd om rond te kijken. Hij slalomt door de mensenmassa heen en schiet lekker op. Ineens staat hij vlak voor een gehandicapte jonge vrouw met een rollator.	Thijs duwt haar zowat omver wanneer hij zich langs haar wurmt, 'aan de kant, lelijke mongool' bijt hij haar toe.	Thijs vervolgt zijn weg naar de winkel en koopt snel wat hij nodig heeft. Daarna baant hij zich zo snel mogelijk een weg terug door de binnenstad. Halverwege voelt hij ineens een zachte aanraking. Hij voelt in zijn zak, merkt dat zijn portemonnee weg is en ziet ineens iemand snel weglopen. Thijs schreeuwt dat hij bestolen is en de dief zet het op een hollen.	…	Thijs	is	blij	als hij ziet hoe de omstanders	de dief grijpen voordat deze weg kan komen
10	Tamara is eigenaar van een koffiezaak. Ze heeft vandaag een aantal sollicitatiegesprekken. Het gaat voornamelijk om tijdelijke krachten die in de vakantieperiode bijspringen. Toch moet ze ook die zorgvuldig selecteren. De eerste drie kandidaten zijn niet super geschikt op het eerste gezicht. Tamara is verbaasd als de volgende een enigszins ruig uitziende Turkse jongen blijkt te zijn.	Tamara interviewt de jongen en uiteindelijk blijkt hij de meest geschikte kandidaat en krijgt de baan.	Aan het einde van de dag is Tamara met het schoonmaakwerk bezig. De balie is schoon en de vloer is geveegd, maar nog niet gedweild. Er zit nog één klant in de hoek en ze zijn officieel nog open. Tamara wacht daarom met dweilen en vult de suikerpotten bij. Tamara loopt met een zwaar dienblad vol suikerpotten als de laatste klant vol tegen haar op botst.	…	Tamara	wordt	blij	als de klant die haar aanstootte	aardig blijkt te zijn en helpt op te ruimen
11	Sander werkt bij een groot softwarebedrijf. De directeur van het bedrijf is kort geleden met pensioen gegaan. Sander zit in de sollicitatiecommissie om een geschikte nieuwe directeur te vinden. De eerste brieven en cv's van kandidaten zijn binnengekomen. Sander print de documenten uit en maakt een overzicht van alle kandidaten. Het valt hem op dat er veel vrouwelijke kandidaten zijn.	Sander denkt dat het goed zou zijn om een vrouw aan het roer te hebben, en hij zet de vrouwelijke kandidaten gewoon op de lijst.	Wanneer Sander klaar is met werken rijdt hij het half uurtje naar huis. Eenmaal thuis besluit hij een eindje te gaan hardlopen. Hij trekt zijn hardloopkleding aan en loopt zijn huis uit. Na wat warming-up en rekken en strekken gaat hij op pad. Na een half uurtje stevig lopen komt Sander bij een smalle straat waar een groepje jongeren de weg blokkeert.	…	Sander	wordt	blij	als het groepje jongeren	gauw aan de kant gaat zodat hij door kan lopen
12	Bart stapt uit de auto en loopt richting het uitvaartcentrum. Hij is hier met een paar andere mensen van het werk voor de uitvaart van een collega. Hij kende de man niet goed, maar sommigen van zijn collega's waren wel close. Hij zoekt samen met zijn collega's een plekje achterin. De dienst begint en na het eerste nummer spreekt zijn vrouw. Een collega van Bart begint zachtjes te huilen.	Bart slaat een arm om haar heen om haar te troosten en haar steun te bieden, wat ze duidelijk op prijs stelt.	Een week later heeft Bart een belangrijke afspraak met zijn manager. Het is een vervroegd kwartaalgesprek om zijn functioneren te evalueren. Bart heeft geen redenen om te denken dat zijn manager iets te klagen zou hebben. Maar zo'n gesprek is toch altijd spannend en stressvol. Als ze eenmaal zitten en de beleefdheden hebben gehad, steekt zijn manager van wal.	…	Bart	is	furieus	wanneer zijn manager hem vertelt	dat hij per direct ontslag krijgt
13	Rianne loopt door het bos met de hond. Het is zaterdagmiddag en er schijnt een waterig zonnetje. Terwijl de hond druk bezig is met snuffelen belt Rianne met een vriendin. Ze hebben elkaar al een poosje niet gesproken en praten bij. Rianne doet een verhaal uit de doeken over een dronken avond op zakenreis. Rianne merkt ineens dat ze langs het kerkhof loopt en dat een groep rouwers bij een nieuw graf staat.	Rianne gaat gelijk zachter praten en hangt snel op, ze knikt de rouwers toe bij wijze van groet en excuus.	Later die dag bedenkt Rianne zich dat ze wel zin heeft om wat te doen die avond. Ze is al een hele poos niet meer op stap geweest. Ze stuurt een berichtje op de groepsapp van een groep vrienden van het volleyballen. Ondertussen gaat Rianne alvast iets te eten klaar maken. Tijdens het eten checkt ze haar telefoon en leest de reacties van iedereen.	…	Rianne	wordt	vrolijk	als ze leest dat iedereen	enthousiast is en ze een leuke avond heeft
14	Mike is op weg naar de sportschool op een doordeweekse avond. Het begint net wat te schemeren en het regent gestaag. Hij steekt over bij de stoplichten en gaat de hoek om. Aan het eind van de straat ziet hij mensen in en uit lopen bij de sportschool. Aan de overkant van de straat fietst een oudere vrouw de andere kant op. Mike ziet hoe ze plots begint te slingeren en met fiets en al omvalt.	Mike bedenkt zich niet en rent er naar toe en helpt haar overeind, ze mankeert niks maar is blij met de hulp.	Eenmaal in de sportschool kleedt Mike zich om. Dan loopt hij snel naar de loopbanden, waar het altijd druk is. De loopbanden staan boven en halverwege de trap ziet hij dat zijn veter los zit. Hij bukt zich om deze te strikken en haast zich dan verder naar boven. Hij ziet dat er nog eentje vrij is, maar een andere man loopt ook op de band af.	…	Mike	is	gepikeerd	wanneer de man die ook wil rennen	net voor hem op de band springt
15	Niels is 's ochtends vroeg als eerste op kantoor. Het is verder muisstil op de afdeling. Na een half uurtje heeft Niels al flink wat werk verzet, dit is zijn favoriete deel van de dag. Hij legt de laatste hand aan een document en streept hem van zijn lijstje. Vervolgens gaat hij koffie halen en komt de eerste collega tegen. Niels kent haar vaag en weet toevallig dat ze sinds kort op dieet is.	Niels zegt goedemorgen en merkt op dat het dieet niet lijkt te helpen, misschien minder suiker in de koffie?	Niels werkt rustig door tot de lunch en daarna heeft hij een vergadering. Zijn team presenteert vandaag de resultaten van een project. Het project is een enorm succes geworden voor het bedrijf. Niels presenteert vandaag zelf niet, maar hij weet dat hij een integraal onderdeel van dit succes is. De belangrijkste ideeën voor het nieuwe programma kwamen van hem.	…	Niels	voelt	blijdschap	wanneer zijn manager in de meeting	hem prijst voor zijn originaliteit en inzet
16	Tom loopt naar huis van de supermarkt aan de overkant van de straat. Hij heeft één tas met boodschappen, maar die weegt niet zoveel. Met zijn vrije hand opent hij de zware portiekdeur van de flat. Achter hem loopt een vrouw met twee tassen, ze heeft moeite met het gewicht. Tom herkent haar en weet dat ze ook de flat in moet. Hij stopt even in de deuropening en denkt na wat te doen.	Hij wacht rustig tot ze er is en houdt de deur vriendelijk voor haar open, wat ze zichtbaar op prijs stelt.	Later die dag wil Tom een afspraak maken bij de dokter. Hij zoekt het nummer op en pakt de telefoon. Hij wil al een hele tijd een afspraak maken vanwege een zere knie. Tom is echter zo druk dat hij vaak geen zin heeft om te bellen, het wachten duurt altijd zo lang. Hij moet eigenlijk al weer de deur uit, maar wil toch even snel bellen.	…	Tom	is	pissig	wanneer de telefoon over gaat	en hij de 15de wachtende blijkt te zijn
17	Elise zit in de bus op weg naar de film. Ze heeft haar oordopjes in en zoekt de filmtijden nog eens op. De bus is halverwege en ze sms't haar vrienden dat ze eraan komt. Elise verandert de muziek naar iets stevigers en zet het harder. Er zitten weinig mensen in de bus behalve twee oudere dames voor haar. Eén van hen draait zich om om te zeggen dat haar muziek wel heel hard staat.	Elise verontschuldigt zich en zet haar muziek meteen zachter, vervolgens wenst ze de dames nog een fijne dag.	Twee haltes verder stapt Elise uit en loopt richting bioscoop. Bij de ingang ontmoet ze haar vrienden en ze gaan gauw naar de zaal. Na de film gaan ze nog even gezellig wat drinken. Elise bestelt een biertje, maar bedenkt zich later en loopt naar de bar om iets anders te bestellen. Ze zegt tegen de serveerster dat ze toch liever een witte wijn heeft.	…	Elise	wordt	vrolijk	als de serveerster reageert	op een vriendelijke manier en ze kletsen wat
18	Wouter werkt op de marketingafdeling van een groot bedrijf. Op dezelfde afdeling werkt zijn beste vriend. Ze werken al jaren heel veel samen en ze zijn een hecht team. Op een ochtend wordt Wouters vriend naar het kantoor van de baas geroepen. Wouter weet niet waar het over gaat, maar hij is wel nieuwsgierig. Later die dag hoort Wouter dat zijn vriend een promotie heeft gekregen en hij niet.	Wouter besluit de vriendschap te beëindigen en verspreidt een roddel dat hij geld zou hebben gestolen.	Later die week heeft Wouter een zeldzame vrije dag. Hij besluit de meubelzaken bij langs te gaan voor nieuwe meubels. Bij IKEA ziet hij een leuke garderobekast, die perfect past in zijn slaapkamer. Wouter aarzelt geen moment en koopt de kast meteen en gaat weer richting huis. Wouter begint thuis meteen enthousiast het bouwpakket in elkaar te zetten.	…	Wouter	is	woedend	als bij het monteren blijkt	dat de planken scheef zijn en er dingen ontbreken
19	Eva loopt snel over het plein richting het station. Ze gaat vandaag naar Haarlem voor een sollicitatiegesprek. Ze heeft zich goed voorbereid en is ruim op tijd van huis gegaan. Vlakbij de ingang naar de stationshal staat een zwerver te bedelen. Wanneer Eva langsloopt, houdt hij een papieren bekertje omhoog met wat kleingeld erin. De man kijkt hoopvol hoe Eva in haar broekzak graaft.	Eva vist wat muntgeld op en gooit dit in het bekertje van de zwerver, ze geeft altijd wat ze kan missen.	Ze loopt snel door en kijkt op het bord welk spoor ze moet hebben. Ze ziet dat er over 2 minuten een trein gaat en zet het op een rennen. Als ze deze trein kan halen, dan komt ze sowieso op tijd. Het is druk in de tunnel naar de sporen en Eva moet steeds uitwijken. Eindelijk bereikt ze de trap en sprint met drie treden per keer naar boven.	…	Eva	is	woedend	als de conducteur haar ziet rennen	de deuren expres voor haar neus sluit
20	Hendrik zit 's ochtends al vroeg op het kantoor. Hij is druk bezig met het tentamen schrijven dat zijn studenten volgende week moeten maken. Terwijl hij daar mee bezig is wordt er op de deur geklopt. Het is één van de studentes uit zijn werkgroep, een knappe verschijning. Het is al gauw duidelijk dat ze probeert Hendrik te verleiden om haar het tentamen in te laten zien. Ze zinspeelt op hoe dankbaar ze zou zijn.	Hendrik maakt misbruik van de situatie en belooft haar het tentamen als ze vanavond bij hem thuis komt.	Later die dag moet Hendrik college geven in een gebouw buiten de binnenstad. Hij pakt de fiets daarheen en trapt stevig door. Op een gegeven moment fietst hij op een drukke kruising af en stopt hij voor het licht. Even later springt het op groen en Hendrik steekt over. Halverwege de kruising vliegt de ketting er ineens af en staat hij midden op de kruising stil.	…	Hendrik	is	getergd	als de auto's die nu groen hebben	ongeduldig beginnen te toeteren en optrekken
21	Larissa is lerares op een basisschool. Ze geeft les aan de bovenbouw, groep vijf en zes. Op een woensdagochtend heeft ze pleinwacht tijdens de pauze. Ze loopt grote rondes over het plein en houdt alles goed in de gaten. Af en toe spreekt ze een leerling aan, maar verder is het rustig. Dan ziet ze een stel leerlingen uit groep 8 aan de rand van het plein rommel staan gooien naar een vrouw die langsloopt.	Larissa wil ze tot de orde roepen, maar ziet dan dat het een moslimvrouw is en laat ze lekker hun gang gaan.	Later die week gaat Larissa naar de bank voor een afspraak. Ze wil haar hypotheek oversluiten en snapt niet veel van financiën, dus wil ze persoonlijk advies. Ze loopt de bank binnen en gaat naar de balie om zich te melden. De receptioniste zegt haar plaats te nemen, ze komen haar zo halen. Larissa gaat zitten en leest een tijdschrift terwijl ze rustig wacht.	…	Larissa	is	laaiend	als ze twee uur later naar buiten loopt	zonder resultaat omdat ze haar afspraak waren vergeten
22	Julia zit op haar plek achter de receptie, de telefoon staat roodgloeiend. Ze handelt het ene na het andere telefoontje af. Voordat Julia het weet is de ochtend alweer voorbij. Na de lunch is het iets minder druk. Dan gaat de telefoon weer, het is iemand voor de man van accounting. De accountant heeft de laatste tijd steken laten vallen en ook nu belt er iemand met een probleem.	Julia geeft de boodschap lekker niet door en geniet wanneer de man daardoor in problemen komt en ontslagen wordt.	Later die week is Julia aan het winkelen. Ze zoekt een specifiek computerspelletje voor de verjaardag van haar broertje. Ze heeft hem beloofd dat hij deze morgen voor zijn verjaardag krijgt. Julia heeft echter weinig tijd gehad om te winkelen de laatste tijd. Eindelijk vind ze één kopie van het spelletje, maar een andere klant ziet hem tegelijkertijd.	…	Julia	is	vrolijk	als de andere klant het spelletje	aan haar geeft zodat ze haar cadeau heeft
23	Inge zit 's middags in de trein op weg naar Groningen. Het is ontzettend rustig in de trein sinds Zwolle. Er zitten naast Inge maar vier mensen in de hele coupé. Er zit een man in de vierzits aan de overzijde van het gangpad een boek te lezen. Wanneer de trein weer stopt staat de man op om uit te stappen. Ineens ziet Inge dat de man een tas onder de bank heeft laten staan.	Inge springt gauw overeind en weet de man nog net voordat de deuren sluiten zijn tas te overhandigen.	Eenmaal in Groningen aangekomen pakt Inge de bus richting Oranjewijk. Ze logeert bij een vriendin en ze gaan naar een concert in de Oosterpoort vandaag. De bus doet er iets langer over dan normaal, maar Inge heeft geen haast. Ze stapt uit en loopt de laatste vijf minuten naar haar huis. Vlak voor ze aanbelt, gaat haar telefoon en ze neemt op.	…	Inge	voelt	woede	wanneer ze te horen krijgt	dat er bij haar is ingebroken en alles weg is
24	Jesse komt 's middags rond half vier aanrijden bij de school. Hij parkeert in de rij wachtende ouders. Er zijn nog geen kinderen op het plein en hij wacht rustig. Hij is zo verzonken in gedachten dat hij niet merkt dat de kinderen naar buiten komen. Hij is dan ook verrast wanneer zijn dochtertje plots achterin klimt. Ze heeft een tekening gemaakt die ze vol trots laat zien en aan hem wil geven.	Jesse wijst haar erop dat ze nog steeds slordig en buiten de lijntjes kleurt en dat ze beter haar best moet doen.	Later die avond als zijn dochtertje al op bed ligt kijkt Jesse voetbal. Hij heeft geld gezet op de uitkomst van deze wedstrijd. Het was een flink bedrag en hij wil graag winnen, want hij kon het geld eigenlijk niet missen. Vlak voor tijd staat het 1-1 en hij heeft geld gezet op 2-1 winst. Hij kijkt gespannen toe hoe zijn team een laatste aanval inzet.	…	Jesse	is	uitgelaten	wanneer vlak voor het einde	zijn team scoort en hij dus 300 euro wint

25	Anouk zit alvast in de vergaderruimte waar ze straks overleg heeft. Ze neemt de documenten die ze heeft voorbereid nog eens door. In de excel sheets van de begroting spot ze ineens een grove fout. Deze fout betekent dat het project veel meer gaat kosten dan ze had gezegd. Anouk heeft weinig zin om dit op te moeten biechten. Voordat ze echt na heeft kunnen denken komen haar bazen binnen.	Anouk besluit de schuld te geven aan een medewerker onder haar die waarschijnlijk toch ontslagen gaat worden.	De vergadering verloopt verder prima en Anouk gaat door met haar dag. Tegen een uur of acht is ze thuis en haalt de post op. Eenmaal binnen ziet ze een brief van het elektriciteitsbedrijf. Het is de jaarafrekening en dat was vorig jaar een dure grap. Anouk heeft toen het voorschot bij laten stellen en hopelijk hoeft ze nu niet bij te betalen.	…	Anouk	is	opgetogen	als uit de brief blijkt dat ze	dit jaar zelf een fiks bedrag terugkrijgt
26	Kevin loopt 's avonds naar het huis van zijn ouders. Hij is bij een vriend geweest die hij niet had gezien sinds hij is verhuisd. Het is schemerig en het is een eind lopen, maar dat vindt Kevin niet erg. Het is fijn om weer door bekende straten te slenteren. Verderop ziet Kevin een gedaante op vier poten uit de bosjes komen. Het is een gewonde hond die zacht jankt en naar hem toe loopt.	Kevin belt gauw de dierenambulance en aait het beestje en stelt hem gerust terwijl hij op de ambulance wacht.	Eenmaal thuis na het incident met de hond gaat Kevin direct naar bed. De volgende dag staat hij vroeg op om nog wat mensen op te zoeken. Hij leent de auto van zijn ouders omdat sommigen buiten de stad wonen. Halverwege, midden tussen de weilanden, houdt de motor er mee op. Na 20 minuten wachten langs de weg komt er eindelijk een andere auto aan.	…	Kevin	is	pissig	wanneer de andere automobilist	wel langzamer gaat rijden, maar niet stopt
27	Bram is op stap met een groepje vrienden. Ze kennen elkaar nog van hun studietijd en spreken af en toe af. Bram heeft al een aantal biertjes op en de rest heeft hem ook aardig zitten. Op een gegeven moment besluiten ze naar de volgende kroeg te gaan. Voor de ingang van de kroeg staat een groepje kerels te roken. Één van Bram zijn vrienden loopt op ze af en begint ruzie te zoeken.	Bram blijft kalm en springt er tussen, hij dwingt zijn vriend excuus te maken voordat de boel kan escaleren.	Daarna is de avond snel afgelopen en gaat Bram naar zijn fiets. Zijn fiets staat helemaal aan de andere kant van het centrum. Het is ondertussen drie uur geweest en hij wil niets liever dan slapen. Het schiet Bram ineens te binnen dat hij zijn fiets helemaal niet op slot heeft gezet. Hij graaft in zijn zakken en kan de sleutel nergens vinden.	…	Bram	is	razend	als hij aankomt waar hij zijn fiets had staan	en hij echt gejat is en hij naar huis kan lopen
28	Myrthe krijgt haar schoonouders vanavond op bezoek en ze blijven eten. Ze wil indruk op hen maken met haar kookkunsten. Ze heeft alleen nog niks in huis en rijdt daarom naar de supermarkt. Myrthe parkeert haar auto op de parkeerplaats en loopt naar de ingang van de supermarkt. Bij de ingang staat een man die overduidelijk dakloos is. De man loopt op Myrthe af en vraagt om wat kleingeld.	Myrthe spuugt naar de man, geeft hem een duw, en sist hem toe 'kruip terug in een hoekje vieze mongool'.	Wanneer Myrthe de boodschappen heeft gekocht, rijdt ze weer naar huis. Thuis aangekomen loopt ze met haar boodschappen naar de voordeur. Myrthe kan haar voordeursleutel niet vinden. Op dat moment bedenkt ze zich dat ze zonder sleutel de deur achter zich dicht heeft gedaan. Snel loopt ze naar het huis van haar buren, die een reservesleutel hebben.	…	Myrthe	is	getergd	als ze bij de buren aanbelt en	die op vakantie zijn en ze een slotenmaker moet bellen
29	Mark rent snel terug richting het eigen doel na de gefaalde aanval. De tegenstander heeft net de bal terugveroverd. Zijn team heeft moeite met terugschakelen naar verdedigen. Over de flank krijgt de spits van de tegenstander een geweldige lange pass. Mark sprint om hem af te snijden op weg naar het doel. Mark zet een tackle in en beseft zich meteen dat hij veel te hard in heeft gezet.	Mark trekt zijn been gauw in omdat hij niemand wil verwonden, ook al kan daardoor de aanval wel doorgaan.	De bal komt bij de keeper terecht en die speelt Mark aan. Mark neemt de bal aan en begint een soloactie langs de lijn. Hij passeert twee man en een derde komt veel te laat aanrennen. Mark trekt naar binnen richting de zestien meter van de tegenstander. Zijn teamgenoten zijn nog niet mee naar voren en dus legt Mark zelf aan om uit te halen.	…	Mark	is	boos	als hij aanlegt om te schieten	en hij keihard onderuit wordt gehaald
30	Joris loopt 's ochtend over de markt richting station. Hij heeft zijn oordopjes in en luistert naar muziek. De marktkooplui zijn in de weer met het opbouwen van hun kramen. De klinkers zijn wit uitgeslagen van de vorst van vannacht. Een man passeert voor Joris met een stapel kisten vol mandarijnen in zijn armen. De bovenste wankelt wat en even later ligt de straat bezaaid met mandarijnen.	Joris stopt en helpt de man gauw met het oprapen van de mandarijnen en maakt ondertussen een praatje.	Later die ochtend komt Joris terug van de lunch. Hij gaat weer achter zijn bureau zitten en kijkt naar zijn lijstje met taken voor vandaag. Hij is lekker opgeschoten vanochtend, maar kan nu eigenlijk niet verder. Hij wacht op bericht van een afdeling in het buitenland. Hij heeft ze gemaild of ze op willen schieten omdat hij niet verder kan zonder de info.	…	Joris	is	blij	als hij een mailtje krijgt met daarin	eindelijk het verlossende woord
31	Tim loopt 's middags door de supermarkt. Op zijn lijstje staat van alles om zelf een verjaardagstaart te maken. Wanneer hij alles heeft gevonden gaat hij naar de kassa. Voor hem in het gangpad loopt een oudere vrouw. Ze is duidelijk ook onderweg naar de kassa, maar ze loopt minder snel dan Tim. Het gangpad is niet zo heel breed en er komt iemand van de andere kant aanlopen.	Tim houdt netjes zijn pas in en laat de oude dame voor gaan in de rij, hij heeft tenslotte geen haast.	Later die dag is hij bezig de taart te bakken, hij staat in de oven. Hij viert vandaag zijn verjaardag en heeft iedereen eigengemaakte taart beloofd. Tim staat niet bepaald bekend om zijn culinaire hoogstandjes. Een aantal van zijn vrienden en familie reageerde dan ook nogal sceptisch. Het is nu een kwestie van eer geworden dat deze taart goed lukt.	…	Tim	is	geïrriteerd	wanneer de cake uiteindelijk	aangebrand uit de oven komt
32	Saskia is vrijwilliger bij een grote manege en vandaag hebben ze open dag. Vanuit de hele omgeving komen ouders en vooral kinderen naar de manege. Er worden rijlessen en workshops over paardenverzorging gegeven. Saskia heeft de hele ochtend workshops gegeven.'s Middags laat ze jonge kinderen ritjes maken in de bak. Wanneer Saskia zich omdraait naar het volgende kind ziet ze meteen dat het downsyndroom heeft.	Saskia doet extra haar best om het meisje een mooie tijd te geven en ze straalt van geluk op het paard.	De volgende ochtend is Saskia vroeg op haar normale werk. Ze werkt bij een groot accountantsbedrijf. Ze moet om 9:00 bij de baas komen en ze weet niet precies waarvoor. Ze heeft er slecht van geslapen vannacht terwijl ze probeerde te verzinnen waar het over zal gaan. Wanneer het eenmaal negen uur is en Saskia bij de baas zit, steekt hij gelijk van wal.	…	Saskia	is	uitzinnig	wanneer de baas in het gesprek	vertelt dat ze promotie krijgt en opslag
33	Dennis stapt de lift in in het ziekenhuis. De vrouw van zijn broer is gisteren bevallen en hij gaat op kraamvisite. Hij heeft een grote knuffel onder zijn arm en een bos bloemen. Hij stapt op de begane grond in en moet op de vijfde verdieping zijn. Op de derde verdieping stopt de lift en stapt een vrouw in. De vrouw is kaal en mager, ze heeft wallen onder haar ogen en een grauwe huid.	Dennis groet haar vriendelijk en maakt een praatje met haar voordat hij op de vijfde verdieping uitstapt.	Wanneer hij eenmaal zijn jongste neefje heeft bekeken gaat Dennis weer. Hij is met de bus, die stopt vlak bij hem voor de deur. Hij is binnen een half uurtje thuis en maakt wat te eten. In de keuken ziet hij op het bord zijn to-do lijstje en herinnert zich dat de tuin een wildernis is. Hij werpt een snelle blik naar buiten om te zien hoe erg het is.	…	Dennis	is	dankbaar	als hij de tuin inkijkt en ziet dat	de buurman al het werk voor hem heeft gedaan
34	Ruben zit op de bank met zijn laptop op schoot wanneer de bel gaat. Hij zet de laptop op de bank naast zich en staat op. Het is een pakketje voor iemand anders in het gebouw. Ruben neemt het aan en ziet op de pakbon staan dat het een geluidsinstallatie is. Ruben gaat weer verder met waar hij mee bezig was. Later die dag gaat de deurbel en het is de buurvrouw die haar pakketje komt halen.	Ruben doet net of hij geen pakje heeft en houdt stug vol tot ze weg gaat, de stereo zet hij in zijn slaapkamer.	De volgende dag is Ruben onderweg naar huis van de supermarkt. Hij heeft twee volle en zware tassen boodschappen. Bij de stoplichten zet hij de tassen naast zich neer en wacht op groen. Wanneer het groen wordt pakt hij de tassen op en haast zich naar de overkant. Bijna aan de overkant struikelt Ruben over de stoeprand en valt tegen de man voor hem aan.	…	Ruben	wordt	vrolijk	wanneer de man voor hem	hem vriendelijk helpt overeind te blijven
35	Jordy loopt over het strand terug naar zijn handdoek. Onderweg komt hij langs een kraampje dat friet en dergelijke verkoopt. Het is er niet druk, het is überhaupt niet druk vandaag. Ondanks dat het een mooie dag is, is er maar een tiental mensen. Jordy loopt voorbij een meisje met een mitella dat probeert een flesje cola te openen. Het wil niet echt lukken met één hand en ze kijkt gefrustreerd.	Jordy blijft staan, lacht haar vierkant uit om haar gestuntel en loopt dan nog steeds geamuseerd door.	Jordy gaat nog een stukje zwemmen in de aanzienlijke golfslag. Daarna gaat hij liggen drogen in de zon en leest zijn boek. Hij gaat helemaal op in het verhaal en de tijd verstrijkt. Op een gegeven moment bedenkt hij zich dat de laatste bus vrij vroeg gaat op zondag. Hij checkt hoe laat het is en bedenkt zich dat hij zal moeten opschieten om de bus te halen.	…	Jordy	is	blij	als de buschauffeur hem ziet	en vriendelijk even wacht zodat hij mee kan
36	Maarten is als enige van zijn team op werkbezoek in het buitenland. De bedoeling is om een kijkje te nemen bij een vergelijkbaar team op het hoofdkantoor. Maarten weet dat dit team op hun projecten uit is. Ze willen maar wat graag dat Maartens afdeling moet sluiten. De teamleider van het rivaliserende team wil een één op één gesprek met Maarten. Hij nodigt Maarten uit om bij hen te komen werken als hij zijn team saboteert.	Maarten denkt alleen maar aan zichzelf en stemt meteen toe, hij hoort toch liever bij het winnende team.	Later die week vliegt Maarten terug naar huis. Het is een lange vlucht en hij heeft onderweg veel om over na te denken. Als het vliegtuig eindelijk landt wacht hij ongeduldig op zijn bagage. Het is hartstikke vroeg en hij wil zijn vrouw verrassen met een ontbijt op bed. Onderweg naar huis koopt hij allerlei lekkere dingen en gaat dan snel door naar huis.	…	Maarten	voelt	blijdschap	als hij eenmaal thuiskomt	en ze samen een romantische ochtend hebben
37	Remco zit in de auto, op weg naar zijn moeder. Hij is de stad nog niet uit en het spoelt van de regen. Het regent onophoudelijk sinds gistermiddag en er hebben zich flinke plassen gevormd. Op sommige plekken staat de straat volledig blank. Er zijn weinig auto's en al helemaal weinig fietsers en voetgangers op straat. Remco stevent op een gigantische plas af en ziet iemand op de stoep lopen.	Remco geeft extra gas om een zo groot mogelijke golf water te creëren, de wandelaar wordt zeiknat gespetterd.	Eenmaal buiten de stad schiet het lekker op op de snelweg. Het blijft rustig op de weg en Remco geniet van het landschap en de rit. Hij heeft de radio hard aan staan en zingt luidkeels mee. Wanneer hij naar het dashboard kijkt om de zender bij te stellen ziet hij een lampje. Hij is vergeten te tanken en de benzine was al heel lang echt bijna op.	…	Remco	is	verheugd	als hij na een paar minuten rijden	een bezinestation ziet en hij kan tanken
38	Frank loopt richting de ingang van het strandpaviljoen. Hij heeft met vrienden afgesproken, maar is aan de vroege kant. Frank loopt blootsvoets over het zand door de branding. Ter hoogte van het paviljoen begint hij aan de klim het strand op. De eerste trede van de trap naar boven is vrij hoog boven het zand. De stap is zo groot dat een jochie van een jaar of vier de trede niet op komt.	Frank hurkt bij het kind neer en helpt hem de trede op en neemt rustig de tijd om hem verder omhoog te helpen.	Frank zoekt binnen een tafel waar iedereen kan zitten en bestelt alvast wat te drinken. Een half uurtje later is iedereen aanwezig en bestellen ze wat te eten. Frank bestelt een biefstuk, medium rare met friet. Wanneer de biefstuk arriveert, blijkt hij bijna well-done te zijn. Frank roept de ober terug om het probleem uit te leggen en laat de biefstuk zien.	…	Frank	is	blij	als de ober reageert op een	vriendelijke en correcte manier
39	Cynthia is op het werk de penningmeester van de personeelsvereniging. Het is een initiatief van en voor werknemers en ze organiseren af en toe gezellige uitjes. Daarnaast verwelkomen ze nieuwe collega's met een cadeautje. Eén keer per jaar innen ze daarvoor vrijwillig contributie van iedereen. Cynthia heeft vorige week een mailtje uitgestuurd met het verzoek te betalen. Ondertussen heeft bijna iedereen het geld overgemaakt.	Cynthia koopt zich die middag een nieuwe winterjas van het geld, er is toch niemand die haar controleert.	De week daarop moet Cynthia naar de dokter. Ze is al een poosje erg vermoeid en heeft weinig eetlust. Ze heeft daarom vorige week bij de dokter bloed laten prikken. De doktersassistente belde gisteren dat ze de volgende ochtend langs moest komen. Het is standaard procedure dit soort nieuws niet aan de telefoon te vertellen, maar ze is toch nerveus.	…	Cynthia	is	opgelucht	als uiteindelijk blijkt dat	de resultaten goed zijn, ze is kerngezond
40	Koen rijdt met de auto door de binnenstad. Het is druk op de weg en er komt een onoverzichtelijke kruising aan. Fietsers en auto's moeten hier de trambaan over vanwege werkzaamheden. Een oudere man op de fiets wankelt onzeker de trambaan op. Het is duidelijk dat de man in de war is en niet goed weet wat hij moet doen. De man komt bijna volledig tot stilstand recht voor Koen zijn auto.	Koen stapt uit om de man veilig naar de overkant te loodsen, terwijl hij de toeterende auto's tot stilte maant.	Drie straten verder slaat Koen rechstaf richting zijn appartement. Hij parkeert een straat verderop waar bijna altijd wel plaats is. Koen zet de motor af en graait zijn tas van de achterbank. Nadat Koen de auto op slot heeft gedaan zwaait hij zijn tas over zijn schouder. Bij de entree van zijn gebouw ziet hij vanuit zijn ooghoek iemand aan komen lopen.	…	Koen	is	laaiend	wanneer de jongen op hem afloopt	ineens Koens tas grijpt en hard wegrent
41	Nina staat 's ochtends vroeg op en komt gelijk in actie. Ze gaat vandaag weer naar huis en ze wil nog winkelen voor ze vliegt. Ze heeft in het huis van vrienden van vrienden gezeten. Ze kent ze verder niet, maar ze hebben haar gratis laten logeren. Nina komt de keuken binnen en trekt de koelkast open. Het lampje springt niet aan en Nina komt er achter dat er een stop is gesprongen.	Nina besluit dat winkelen minder belangrijk is en laat een electricien komen die ze vervolgens zelf betaalt.	Later die dag rent Nina beladen met al haar bagage naar de trein. Ze springt net op tijd in de trein richting het vliegveld. De trein vertrekt op tijd maar tien minuten later staan ze ineens stil tussen de weilanden. Nina begint zich zorgen te maken dat ze te laat gaat komen. Een uur later dan gepland komt de trein eindelijk aan op Schiphol.	…	Nina	is	verbolgen	als ze aankomt op het vliegveld en	blijkt dat ze te laat is om nog te boarden
42	Thomas zit in de kroeg met vrienden. Het is een mannenavondje en ze drinken er lustig op los. Thomas heeft vakantie en zijn vriendin is een week bij haar ouders. Hij mist haar wel, maar aan de andere kant is wat vrijheid ook wel fijn. Als iedereen besluit naar huis te gaan blijft Thomas nog even hangen. Hij raakt aan de praat met een mooie vrouw aan de bar die hem wel ziet zitten.	Thomas maakt een praatje met haar en merkt terloops op dat hij een vriendin heeft, en gaat later alleen naar huis.	De volgende dag sleept Thomas zich rond twee uur naar de supermarkt. Hij heeft niks meer in huis en ondanks een lichte kater toch honger. Wanneer hij uit de supermarkt komt zet hij de tassen even neer. Hij heeft gelijk maar grote boodschappen gedaan en het weegt heel wat. Wanneer hij bij zijn flat aankomt, ziet hij iemand vlak voor zich de deur openen.	…	Thomas	is	verheugd	als de man omkijkt, even wacht en	de deur vriendelijk glimlachend open houdt
43	Leonie gaat naar de schouwburg voor een toneelvoorstelling. Het is druk, want het is een mooi stuk en er spelen veel bekende acteurs mee. Voor de kassa staat een enorme rij, waar Leonie achter aansluit. Plots hoort Leonie iemand verderop haar naam roepen. Verderop in de rij staat een vriend van Leonie naar haar te zwaaien. Hij wenkt Leonie en gebaart dat ze bij hem in de rij moet komen staan.	Leonie loopt naar voren en sluit bij haar vriend aan, een vrouw die er wat van zegt scheldt ze uit voor hoer.	Na de voorstelling loopt Leonie naar de garderobe. Ze had een jas en een paraplu meegenomen, omdat er stevige buien waren voorspeld. Bij de garderobe staat personeel dat de jassen en dergelijk ophaalt. Leonie haalt het garderobenummer tevoorschijn dat ze had gekregen. Ze geeft het nummer aan het garderobepersoneel, dat op zoek gaat naar haar spullen.	…	Leonie	voelt	irritatie	wanneer het garderobeperson eel	pas na tien minuten haar spullen vindt en ze zeiknat wordt
44	Laura komt pas om een uur of acht uit een vergadering. Ze is moe, maar tevreden over de uitkomst. Ze besluit naar huis te lopen omdat het een mooie zomeravond is. Eenmaal uit de drukte van het centrum is het heerlijk rustig op straat. Een paar straten van haar huis ziet Laura het portier van een auto op een kier staan. Ze kijkt rond of er iemand in de buurt is, maar er is niemand.	Laura denkt na en besluit dat ze het beste gewoon het portier kan sluiten zodat niemand de auto leeg kan halen.	De volgende ochtend staat Laura vroeg op en gaat naar haar werk. Ze is nieuwsgierig naar de uitkomst van een subsidieaanvraag die ze heeft ingediend. Bij de vergadering van gisteren heeft ze geprobeerd de laatste twijfelaars over te halen. Het leek erop dat de aanvraag ingewilligd ging worden. Laura probeert gewoon te werken terwijl ze op nieuws wacht.	…	Laura	is	woedend	wanneer blijkt dat haar aanvraag	is afgekeurd om een enorm domme reden
45	Wendy komt wat laat aan bij een feestje van haar broer. Het is een soort housewarming. Haar broer heeft zowel vrienden en familie als collega's uitgenodigd. Het is een flink groot feest, maar Wendy haar broer had geen zin om drie keer iets te organiseren. Wendy wordt gespot door haar broer en her en der voorgesteld. Dan stelt haar broer haar voor aan een collega die een mismaakte hand heeft.	Wendy schudt de man de hand alsof ze het niet doorheeft en groet hem vriendelijk, ze wil de man niet beledigen.	Later op de avond staat Wendy met wat familieleden te kletsen. Een aantal neven en een tante zijn ook aanwezig. Wendy heeft ze al een poos niet gezien en ze praat ze bij over haar leven. Wanneer Wendy even naar de wc gaat en weer terugkomt, treft ze iedereen lachend aan. Ze stoppen abrupt als ze er weer bij komt staan en Wendy kijkt ze vragend aan.	…	Wendy	is	vrolijk	wanneer ze even later	ook bij de grap wordt betrokken en meelacht
46	Michael gaat met zijn vrienden naar een voetbalwedstrijd. Hun club speelt uit tegen de aartsrivaal. De sfeer is gespannen, want de winnaar heeft grote kans kampioen te worden. Ze komen in een vak met medesupporters te zitten. Voor aanvang wordt er om een minuut stilte gevraagd voor een overleden speler van de tegenpartij. Het is rumoerig in het vak en ook Michaels vrienden praten en lachen.	Michael heeft weinig respect voor de tegenstander en zet luidkeels het clublied in, en de rest zingt mee.	Een week later gaat Michael op vakantie. Hij vliegt 's ochtends vroeg naar Cyprus, waar hij een week in de zon wil liggen. Op internet heeft hij een goedkoop appartementje geboekt. De foto's van het appartement waren wat onduidelijk, maar Michael kon de aanbieding niet laten gaan. Eenmaal geland op Cyprus huurt Michael een auto en rijdt naar het appartement.	…	Michael	is	furieus	wanneer bij aankomst blijkt dat het	appartement niet bestaat en hij nergens heen kan
47	David staat op de steiger op het punt om te beginnen te schuren. Het is halverwege de ochtend en het is al flink heet. Het is de derde week van deze klus en ze zijn met het laatste stuk bezig. Hij is alleen aan het werk aan deze kant, zijn collega's zijn achter bezig. Hij ziet door het raam een vrouw achter een bureau zitten. Haar telefoon gaat en ze neemt aan en begint een heel gesprek.	David wil haar niet storen en wacht even tot ze klaar is en waarschuwt dan dat hij lawaai gaat maken.	Na de lunch klimt David weer de steiger op. Hij gaat verder met het schuurwerk, ondertussen is hij bij het laatste raam. Het werk is goed opgeschoten ondanks de hitte. David werkt gestaag door en al gauw is ook het laatste kozijn geschuurd. David zet de machine uit en terwijl hij zijn bezwete voorhoofd afveegt glipt de schuurmachine uit zijn vingers.	…	David	is	opgelucht	als hij ziet hoe het apparaat	niet van de steiger valt en nog heel is
48	Kim stapt 's middags in de tram naar huis. Ze checkt in met haar pas en loopt door naar achter om een plaatsje te zoeken. Achter Kim stapt ook nog een vrouw in, ze loopt achter haar aan. Ze is hoogzwanger en volgt Kim verder de tram in. Kim ziet achterin nog één vrije zitplaats en stevent er op af. Voordat Kim de zitplaats bereikt begint de tram schokkend weer te rijden.	Kim aarzelt niet en stapt opzij zodat de zwangere vrouw rustig kan gaan zitten in de slingerende tram.	Eenmaal thuis neemt Kim de stapel post door. Er zit een zoveelste brief bij van de belastingdienst. Ze is al maanden bezig om haar aanslag gecorrigeerd te krijgen. De belastingdienst heeft een fout gemaakt en weigert die te herstellen. Ze hoopt dat het eindelijk is opgelost, ze heeft er genoeg van.	…	Kim	is	gefrustreerd	wanneer blijkt dat haar aanslag	nog steeds niet klopt
49	Kelly loopt door het park op weg naar een feestje. Het schemert al, maar het is nog redelijk licht. Het is een warme dag geweest en er liggen nog mensen op handdoeken op het gras. Kelly pakt haar telefoon om op te zoeken waar ze precies moet zijn. Dan ziet ze vanuit haar ooghoek een portemonnee liggen. Er is niemand te bekennen en Kelly pakt de portemonnee op die vol geld blijkt te zitten.	Kelly haalt alle cash eruit, 100 euro, en gooit de portemonnee weer terug op de grond, dat is mooi meegenomen.	Kelly vervolgt haar weg naar het feestje en gaat daarna nog even de kroeg in. De volgende ochtend komt ze redelijk op tijd uit bed. Gelukkig heeft ze geen kater want ze moet nog voor haar studie aan de slag vandaag. Ze zet de laptop aan en gaat dan eerst koffie maken. Als ze terug komt opent ze haar mail en ziet een mailtje van de studieadviseur.	…	Kelly	is	dolblij	als ze in de email leest dat ze	een herkansing mag doen en kan afstuderen

50	Adriana loopt door het bos op een zondagochtend. Het is vroeg in de herfst en de bladeren beginnen net te verkleuren. Ze heeft onderweg koffie gehaald bij de enige koffiezaak die op een zondagochtend open is. Terwijl ze wandelt, neemt ze af en toe een slokje. Ze loopt graag in dit bos, het is redelijk groot en ze komt doordeweeks niet veel buiten. Halverwege haar gewone route heeft ze haar koffie op.	Adriana gooit het bekertje in de bosjes en loopt rustig verder, iemand anders raapt het vast wel weer op.	Een uur later loopt Adriana nog steeds in het bos. Ze is ondertussen een heel eind bij de auto vandaan, maar ze is nog lang niet moe. Dan komt haar voet ineens in een gat terecht en verzwikt ze haar enkel. Ze voelt een scherpe pijn en valt om omdat ze niet meer op dat been kan staan. De pijn bonst stug door in haar enkel en ze kan niet anders dan langs het pad blijven zitten.	…	Adriana	voelt	boosheid	als er even later iemand langskomt	en diegene haar vraag om hulp compleet negeert
51	Judith zit op haar fiets voor de supermarkt. Ze is met een groepje van drie vrienden en ze hangen maar wat. Terwijl ze wat dom ouwehoeren ziet Judith een hardloper aankomen. De loper is hevig bezweet en het is hem aan te zien dat hij tot het uiterste gaat. Judith en haar vrienden staan pal in het pad van de hardloper. Judith haar vrienden doen een paar stappen opzij om plaats te maken.	Judith stapt snel van haar fiets af en tilt haar fiets aan de kant zodat de loper ongehinderd door kan lopen.	Later die dag loopt Judith van een terrasje door de stad naar huis. Het is nog redelijk vroeg, maar het begint al te schemeren. De zomer loopt ten einde, maar de temperatuur is nog lekker. Judith steekt de brug over en merkt ineens dat haar portemonnee niet in haar zak zit. Ze zou zweren dat ze hem net nog had en draait zich om om te kijken of hij ergens ligt.	…	Judith	voelt	opluchting	als ze zich omdraait en	een man haar portemonnee teruggeeft
52	Kirsten hangt wat rond met haar vrienden in het park. Ze drinken biertjes en zijn wat aangeschoten en baldadig. Een van Kirstens vrienden die meedrinkt begint opmerkingen te maken over mensen die langslopen. Eerst zachtjes, maar later steeds harder. Plotseling loopt een donkere jongen langs het groepje vrienden. Kirstens vriend roept een racistische opmerking naar de jongen en daagt hem uit.	Kirsten lacht lekker mee en doet er een schepje bovenop door de jongen ook uit te schelden en hem te bespugen.	De volgende dag gaat Kirsten naar de elektronicazaak. Ze heeft al een tijd een Ultra HD-tv op het oog en vandaag wil ze hem kopen. De tv is erg duur en Kirsten heeft er lang voor gespaard. Ze heeft de achterbank van haar auto vast naar beneden geklapt om ruimte te maken. Aangekomen bij de winkel vraagt Kirsten direct een medewerker naar de tv die ze wil.	…	Kirsten	voelt	boosheid	wanneer in de winkel blijkt dat	de tv die ze wil niet meer op voorraad blijkt te zijn
53	Danny is aan het werk in de werkplaats van zijn fietsenzaak. Hij is bezig een achterwiel te vervangen. Het is halverwege de ochtend en nog lekker rustig. Wanneer de bel gaat maakt Danny snel af wat hij aan het doen was. Hij veegt zijn handen schoon en loopt dan snel naar voren. Er staat een vrouw met een hoofddoek te wachten en het blijkt al gauw dat haar Nederlands niet heel goed is.	Danny schudt meewarig het hoofd, negeert de vrouw straal en gaat weer verder in de werkplaats, domme allochtonen.	Later die dag, na de lunch, worden er onderdelen geleverd. Danny tekent voor ontvangst en begint de pakketjes te sorteren. Er is één bepaald onderdeel waar hij al een hele tijd op zit te wachten. Het is een heel speciaal onderdeel voor een luxe mountainbike. Het onderdeel is al twee keer verkeerd geleverd en de klant begint ongeduldig te worden.	…	Danny	voelt	opluchting	dat het binnengekomen onderdeel	nu wel het goede blijkt te zijn, eindelijk!
54	Sarah heeft de nachtdienst op de verpleegafdeling. Het is tijd om een ronde te lopen en haar collega en zij doen elk de helft. Sarah controleert alle infusen en kijkt of iedereen goed slaapt. Ze gaat stilletjes van kamer tot kamer. Op één van de laatste kamers ziet ze een portemonnee liggen. Hij is van de oudere man die vanmiddag zijn kleinkinderen met hun rapporten op bezoek had.	Sarah kijkt snel om zich heen, haalt er 50 euro uit en vervolgt haar ronde, de man heeft toch niet lang meer.	Aan het einde van haar dienst gaat Sarah gauw richting huis. Eenmaal thuis zet ze een kop thee en zet haar laptop aan. Met de laptop in één hand en de thee in de andere loopt ze richting de woonkamer. Ze ziet door het voorraam dat de zon ondertussen op is gekomen. Dan struikelt ze ineens over de drempel en laat de laptop vallen en morst thee.	…	Sarah	constateert	opgelucht	dat de laptop als ze hem weer aanzet	het weer gewoon blijkt te doen, gelukkig!
55	Lieke zit met een vriendin op het terras. Het is donderdagmiddag en ze hebben allebei lekker vakantie. Haar vriendin moet naar het toilet, waar het waarschijnlijk stervensdruk zal zijn. Terwijl Lieke alleen zit komt een vriend van haar langsfietsen. Hij vertelt Lieke over een feestje vanavond en moet gelijk verder. Lieke wil wel heen, maar ze heeft eigenlijk al plannen gemaakt met die andere vriendin.	Lieke vertelt eerlijk over het feestje en vraagt of de vriendin het leuk zou vinden om daarheen te gaan.	Later die middag springt Lieke gauw onder de douche thuis. Onder het afdrogen bedenkt ze zich dat ze cash geld nodig zal hebben. Dat herinnert haar eraan dat haar salaris één dezer dagen binnen moet komen. Nadat ze zich heeft aangekleed rommelt ze in de la om de random reader te vinden. Eenmaal gevonden logt ze gauw in om haar saldo te checken.	…	Lieke	constateert	opgetogen	dat de salarisadminstrati e	heeft gezorgd dat haar salaris op tijd is dit keer
56	Sanne zit naar een voorstelling onder regie van een vriend te kijken. Het stuk is ongeveer halverwege en Sanne is al volledig uitgecheckt. De acteurs zijn niet bijster goed en het decor is amateuristisch. Ook het verhaal zelf kan niet echt boeien. Sanne heeft sterk de neiging om halverwege naar huis te gaan. Maar ze blijft toch tot de receptie en komt daar haar vriend, de regisseur, tegen.	Sanne schudt hem de hand en steekt vervolgens van wal en vertelt keihard hoe slecht ze het stuk vond.	Na de receptie loopt Sanne richting het dichtstbijzijnde grote plein. De trams rijden al niet meer en het begint steeds harder te regenen. Eenmaal bij het plein aangekomen wil ze een taxi aanhouden. Er zijn echter weinig taxi's en veel mensen die een taxi willen vanwege de regen. Eindelijk weet ze een taxi aan te houden en deze stopt vlakbij haar.	…	Sanne	is	opgetogen	wanneer de taxi vlak voor haar stopt	en ze in kan stappen voor ze doorweekt is
57	Stefan zit achter de computer op het werk. Hij is bezig concertkaartjes te bestellen voor hem en drie collega's in zijn team. Ze gaan wel vaker met z'n allen iets doen buiten het werk om. Het is ondertussen meer een vriendengroep dan collega's. Er is een nieuw teamlid dat er nog niet echt bij hoort. Dat nieuwe teamlid komt even later binnen en zegt ook fan te zijn van die band.	Stefan nodigt hem uit om mee te gaan, zodat hij zich meer thuis gaat voelen als nieuweling binnen een hecht team.	Tegen half vijf besluit Stefan dat het leuk is geweest voor die week. Hij sluit zijn computer af en pakt zijn spullen bij elkaar. Voor vijven is hij op het station en wacht hij op de trein. Het nadeel van op tijd weggaan op vrijdag is dat hij niet de enige is. Het is hartstikke druk op het perron en hij ziet dat de binnenkomende trein ook al goed vol zit.	…	Stefan	is	opgetogen	wanneer in het gedrang voor de inagng	iemand hem vriendelijk voor laat gaan
58	Maria loopt met een ingepakt cadeau over straat. Ze is onderweg naar een kraamvisite bij een collega. Maria heeft niks met baby's en gelukkig heeft iemand anders het cadeau uitgezocht. Maria belt aan en het duurt even voordat de deur opengaat. De kamer zit vol met familie en vrienden die ze niet kent. De baby wordt uiteraard meteen getoond en Maria vindt het maar een lelijke baby.	Maria zegt desgevraagd dat het een mormel is en dat ze sowieso niet snapt wat er mooi is aan baby's.	Wanneer Maria bij de kraamvisite vandaan komt loopt ze het centrum in. Ze heeft honger en gaat op zoek naar iets warms voor de lunch. Ze schiet een döner tent binnen en bestelt een broodje döner. Ze gaat zitten en valt meteen aan wanneer het broodje klaar is. Bij de eerste hap valt er gelijk een enorme klodder saus uit de zijkant van het broodje.	…	Maria	is	opgelucht	als de klodder gemorste saus	op tafel valt en niet op haar nieuwe blouse
59	Martijn fietst door de stad naar de sportschool. Hij heeft een lange dag achter de rug en heeft zin om intensief te sporten. Martijn is halverwege wanneer hij door een erg smal steegje fietst. Een bejaarde man loopt langzaam in dezelfde richting door het steegje. In het voorbijgaan schampt Martijn met zijn fiets de oude man. De man komt ten val en Martijn hoort hem kermen van de pijn.	Martijn beseft dat hij fout zat, maar doet alsof er niks gebeurd is en fietst door, laat een ander die ouwe helpen.	De volgende dag staat Martijn vroeg op. Hij heeft een sollicitatiegesprek bij een bedrijf voor een leidinggevende functie. Het bedrijf is gevestigd in een nabijgelegen stad, 20 kilometer verderop. Omdat Martijn geen rijbewijs heeft reist hij met openbaar vervoer. Hij wil voor de zekerheid een trein eerder nemen en zorgt dat hij op tijd op het station is.	…	Martijn	is	kwaad	wanneer hij op het perron komt en	hoort omroepen dat er geen treinen rijden
60	Ilse staat te wachten bij de pinautomaat. Er staat één man voor haar en niemand achter haar. Terwijl ze wacht kijkt Ilse wat op haar telefoon en bedenkt hoeveel geld ze op zal nemen. Vanuit haar ooghoek ziet Ilse dat de man voor haar bijna klaar is. Ilse stopt haar telefoon weg en pakt haar portemonnee. Als de man wegloopt, ziet Ilse een briefje van 50 vallen maar de man heeft niks door.	Ilse roept de man gauw na en pakt het briefje van 50 op om terug te geven, de man bedankt Ilse uitvoerig.	Later die avond is Ilse onderweg naar huis. Ze bedenkt zich ineens dat ze niks meer voor het ontbijt in huis heeft. Ze kijkt hoe laat het is en gaat meteen wat sneller lopen. Er is gelukkig een Albert Heijn vlakbij, maar die gaat al bijna dicht. Als Ilse de hoek om loopt ziet ze hoe een medewerker een bord binnenhaalt en ze gaat nog sneller lopen.	…	Ilse	is	pissig	wanneer de medewerker haar ziet	haar vuil aankijkt en de deur snel sluit
61	Emma loopt over de Dam richting de Kalverstraat. Ze moet een verjaardagscadeautje halen voor een vriend. Het is gelukkig niet heel druk, maar het loopt al tegen sluitingstijd. Op de hoek van de Kalverstraat zit een blinde straatmuzikant. Hij speelt de sterren van de hemel en zingt met een doorleefde stem. Op de gitaarhoes voor hem ligt al heel wat kleingeld en zelfs een paar briefjes.	Emma heeft alleen een tientje, maar de muzikant is zo goed dat ze het hem gunt, hij kan het vast gebruiken.	Later die week is de verjaardag waarvoor ze op pad was. Er zijn al gauw 25 mensen en het feestje is in volle gang. Emma kent echter niet veel van de mensen die er zijn, en bekenden zijn er nog niet. Ze probeert zich in het gesprek van een groepje naast zich te mengen. Emma gaat erbij staan en wanneer een stilte valt doet ze ook een duit in het zakje.	…	Emma	voelt	woede	opkomen wanneer het groepje	haar aanstaart en vervolgens negeert
62	Jeffrey komt na een dag hard werken thuis. Net op het moment dat hij de voordeur achter zich dicht trekt begint het te onweren. Het bliksemt en begint hard te regenen. Dan ziet Jeffrey dat er die dag post is bezorgd bij hem. Tussen de folders en rekeningen die zijn bezorgd ziet Jeffrey een rouwkaart liggen. De kaart blijkt verkeerd bezorgd te zijn en eigenlijk bestemd voor zijn overburen.	Jeffrey heeft geen zin om zich nat te laten regenen en verscheurt de rouwkaart en gooit hem in de prullenbak.	Het noodweer blijft die dag aanhouden. Jeffrey hoort dat het KNMI code rood afgeeft en aanraadt binnen te blijven. Het begint hard te hagelen en Jeffrey ziet buiten enorme hagelstenen uit de lucht vallen. Een hagelsteen zo groot als een golfbal landt in Jeffreys tuin en Jeffrey vreest voor zijn nieuwe auto. Zodra het droog is gaat Jeffrey de schade opnemen.	…	Jeffrey	voelt	frustratie	wanneer hij bij de auto aan komt lopen	en die onder de kleine deuken zit van de hagelstenen
63	De afdeling waar Wesley werkt heeft het heel erg druk. De opdrachten blijven maar binnenstromen. Daarom is er kort geleden een nieuwe medewerker aangenomen om het team te versterken. De nieuwe medewerker is een jong en knap meisje dat pas afgestudeerd is. Op een dag komt Wesley haar alleen tegen in het kopieerhok. Ze staat bij het kopieerapparaat en heeft duidelijk ruzie met het apparaat.	Wesley gaat achter haar staan, vraagt wat er is, en streelt zogenaamd per ongeluk de billen van het meisje.	Grijnzend werkt Wesley die middag verder en gaat om vijf uur naar huis. Thuis aangekomen parkeert hij zijn auto en loopt naar de voordeur van zijn huis. Wesley hoopt dat bij de post de dvd zit die hij besteld had. Hij had de hele vond vrijgehouden om het nieuwe seizoen van de tv serie te kijken. Wesley doet de deur open en ziet een stapel post op de deurmat.	…	Wesley	is	geërgerd	wanneer hij de post doorbladert	de dvd er niet bijzit, maar wel een boete
64	Sandra heeft al een tijdje een conflict met haar buren. Ze zet haar auto vaak op de parkeerplek voor de deur van haar buren. Haar buurman en buurvrouw beweren echter dat die plek speciaal voor hun auto is. Op een dag hoort Sandra van de andere buren dat haar buurman verongelukt is. Sandra aarzelt niet en loopt direct naar het huis van haar buren. Ze belt aan en haar buurvrouw doet de deur open.	Sandra bijt haar toe dat haar man toch een vervelende vent was en het waarschijnlijk verdiende om dood te gaan.	Sandra loopt weer naar huis en gaat verder met koken. Die avond pakt Sandra haar tablet en leest het financiële nieuws. Sandra heeft kort geleden veel van haar spaargeld in aandelen gestoken. Ze houdt sindsdien de markt goed in de gaten en nu maakt ze zich zorgen. Ze heeft slechte berichten gehoord en checkt de koersen van de aandelen in haar portfolio.	…	Sandra	is	woedend	als ze de gegevens doorleest	en haar aandelen niks meer waard blijken te zijn

Experimental Stimulus List 3

Number	Introduction	Character Manipulation	Continuation	Transition	Name	Verb	Affective State Adjective	Neutral Segment	Affect Reason
1	Wesley heeft al een tijdje een conflict met zijn buren. Hij zet zijn auto vaak op de parkeerplek voor de deur van zijn buren. Zijn buurman en buurvrouw beweren echter dat die plek speciaal voor hun auto is. Op een dag hoort Wesley van de andere buren dat zijn buurman verongelukt is. Wesley aarzelt niet en loopt direct naar het huis van zijn buren. Hij belt aan en zijn buurvrouw doet de deur open.	Wesley condoleert haar en biedt aan de komende dagen voor haar te koken en wat ze verder maar nodig heeft.	Wesley loopt weer naar huis en gaat verder met koken. Die avond pakt Wesley zijn tablet en leest het financiële nieuws. Wesley heeft kort geleden veel van zijn spaargeld in aandelen gestoken. Hij houdt sindsdien de markt goed in de gaten en nu maakt hij zich zorgen. Hij heeft slechte berichten gehoord en checkt de koersen van de aandelen in zijn portfolio.	…	Wesley	is	dolblij	als hij de gegevens doorleest	en zijn aandelen in waarde verdubbbeld zijn
2	De afdeling waar Sander werkt heeft het heel erg druk. De opdrachten blijven maar binnenstromen. Daarom is er kort geleden een nieuwe medewerker aangenomen om het team te versterken. De nieuwe medewerker is een jong en knap meisje dat pas afgestudeerd is. Op een dag komt Sander haar alleen tegen in het kopieerhok. Ze staat bij het kopieerapparaat en heeft duidelijk ruzie met het apparaat.	Sander helpt haar en legt uit hoe het apparaat werkt, ze is duidelijk opgelucht en bedankt hem vriendelijk.	Grijnzend werkt Sander die middag verder en gaat om vijf uur naar huis. Thuis aangekomen parkeert hij zijn auto en loopt naar de voordeur van zijn huis. Sander hoopt dat bij de post de dvd zit die hij besteld had. Hij had de hele avond vrijgehouden om het nieuwe seizoen van de tv serie te kijken. Sander doet de deur open en ziet een stapel post op de deurmat.	…	Sander	is	blij	wanneer hij de post doorbladert	en de dvd erbij zit en hij dus door kan kijken
3	Marloes komt na een dag hard werken thuis. Net op het moment dat ze de voordeur achter zich dicht trekt begint het te onweren. Het bliksemt en begint hard te regenen. Dan ziet Marloes dat er die dag post is bezorgd bij haar. Tussen de folders en rekeningen die zijn bezorgd ziet Marloes een rouwkaart liggen. De kaart blijkt verkeerd bezorgd te zijn en eigenlijk bestemd voor zijn overburen.	Marloes trekt haar jas weer aan en loopt door de regen naar het huis van haar buren om de kaart netjes af te geven.	Het noodweer blijft die dag aanhouden. Marloes hoort dat het KNMI code rood afgeeft en aanraadt binnen te blijven. Het begint hard te hagelen en Marloes ziet buiten enorme hagelstenen uit de lucht vallen. Een hagelsteen zo groot als een golfbal landt in Marloes' tuin en Marloes vreest voor haar nieuwe auto. Zodra het droog is gaat Marloes de schade opnemen.	…	Marloes	voelt	opluchting	wanneer ze bij de auto aan komt lopen	en deze geen beschadigingen blijkt te hebben
4	Mike loopt over de Dam richting de Kalverstraat. Hij moet een verjaardagscadeautje halen voor een vriend. Het is gelukkig niet heel druk, maar het loopt al tegen sluitingstijd. Op de hoek van de Kalverstraat zit een blinde straatmuzikant. Hij speelt de sterren van de hemel en zingt met een doorleefde stem. Op de gitaarhoes voor hem ligt al heel wat kleingeld en zelfs een paar briefjes.	Mike buigt zich alsof hij wat geeft maar grist in plaats daarvan snel een handvol munten en briefjes mee.	Later die week is de verjaardag waarvoor hij op pad was. Er zijn al gauw 25 mensen en het feestje is in volle gang. Mike kent echter niet veel van de mensen die er zijn, en bekenden zijn er nog niet. Hij probeert zich in het gesprek van een groepje naast zich te mengen. Mike gaat erbij staan en wanneer een stilte valt doet hij ook een duit in het zakje.	…	Mike	voelt	blijdschap	opkomen wanneer het groepje	hem gelijk welkom doet voelen
5	Bart staat te wachten bij de pinautomaat. Er staat één man voor hem en niemand achter hem. Terwijl hij wacht kijkt Bart wat op zijn telefoon en bedenkt hoeveel geld hij op zal nemen. Vanuit zijn ooghoek ziet Bart dat de man voor hem bijna klaar is. Bart stopt zijn telefoon weg en pakt zijn portemonnee. Als de man wegloopt ziet Bart een briefje van 50 vallen maar de man heeft niks door.	Bart houdt zich lekker stil en steekt de 50 euro gauw in zijn zak, eigen schuld, wat een domme vent!	Later die avond is Bart onderweg naar huis. Hij bedenkt zich ineens dat hij niks meer voor het ontbijt in huis heeft. Hij kijkt hoe laat het is en gaat meteen wat sneller lopen. Er is gelukkig een Albert Heijn vlakbij, maar die gaat al bijna dicht. Als Bart de hoek om loopt ziet hij hoe een medewerker een bord binnenhaalt en hij gaat nog sneller lopen.	…	Bart	is	dankbaar	wanneer de medewerker hem ziet	en toch nog even snel binnenlaat
6	Kirsten fietst door de stad naar de sportschool. Ze heeft een lange dag achter de rug en heeft zin om intensief te sporten. Kirsten is halverwege wanneer ze door een erg smal steegje fietst. Een bejaarde man loopt langzaam in dezelfde richting door het steegje. In het voorbijgaan schampt Kirsten met haar fiets de oude man. De man komt ten val en Kirsten hoort hem kermen van de pijn.	Kirsten stopt direct, gaat terug naar de man, belt een ambulance en geeft de man haar jas om hem warm te houden.	De volgende dag staat Kirsten vroeg op. Ze heeft een sollicitatiegesprek bij een bedrijf voor een leidinggevende functie. Het bedrijf is gevestigd in een nabijgelegen stad, 20 kilometer verderop. Omdat Kirsten geen rijbewijs heeft reist ze met openbaar vervoer. Ze wil voor de zekerheid een trein eerder nemen en zorgt dat ze op tijd op het station is.	…	Kirsten	is	euforisch	wanneer ze op het perron komt en	op tijd blijkt te zijn en de juiste trein haalt
7	Maria loopt met een ingepakt cadeau over straat. Ze is onderweg naar een kraamvisite bij een collega. Maria heeft niks met baby's en gelukkig heeft iemand anders het cadeau uitgezocht. Maria belt aan en het duurt even voordat de deur opengaat. De kamer zit vol met familie en vrienden die ze niet kent. De baby wordt uiteraard meteen getoond en Maria vindt het maar een lelijke baby.	Maria zegt dat natuurlijk niet en ze complimenteert de moeder met zo'n ontzettend schitterende baby.	Wanneer Maria bij de kraamvisite vandaan komt loopt ze het centrum in. Ze heeft honger en gaat op zoek naar iets warms voor de lunch. Ze schiet een döner tent binnen en bestelt een broodje döner. Ze gaat zitten en valt meteen aan wanneer het broodje klaar is. Bij de eerste hap valt er gelijk een enorme klodder saus uit de zijkant van het broodje.	…	Maria	is	nijdig	als de klodder gemorste saus	op haar nieuwe blouse valt en dus verpest is
8	Stefan zit achter de computer op het werk. Hij is bezig concertkaartjes te bestellen voor hem en drie collega's in zijn team. Ze gaan wel vaker met z'n allen iets doen buiten het werk om. Het is ondertussen meer een vriendengroep dan collega's. Er is een nieuw teamlid dat er nog niet echt bij hoort. Dat nieuwe teamlid komt even later binnen en zegt ook fan te zijn van die band.	Stefan liegt en zegt dat ze toch niet gaan omdat de kaartjes te duur zijn en bestelt later alsnog de kaartjes.	Tegen half vijf besluit Stefan dat het leuk is geweest voor die week. Hij sluit zijn computer af en pakt zijn spullen bij elkaar. Voor vijven is hij op het station en wacht hij op de trein. Het nadeel van op tijd weggaan op vrijdag is dat hij niet de enige is. Het is hartstikke druk op het perron en hij ziet dat de binnenkomende trein ook al goed vol zit.	…	Stefan	is	boos	wanneer in het gedrang voor de inagng	iemand hem zonder pardon omver duwt
9	Sanne zit naar een voorstelling onder regie van een vriend te kijken. Het stuk is ongeveer halverwege en Sanne is al volledig uitgecheckt. De acteurs zijn niet bijster goed en het decor is amateuristisch. Ook het verhaal zelf kan niet echt boeien. Sanne heeft sterk de neiging om halverwege naar huis te gaan. Maar ze blijft toch tot de receptie en komt daar haar vriend, de regisseur, tegen.	Sanne schudt hem de hand en feliciteert hem van harte, toch zoveel mogelijk positief commentaar gevend.	Na de receptie loopt Sanne richting het dichtstbijzijnde grote plein. De trams rijden al niet meer en het begint steeds harder te regenen. Eenmaal bij het plein aangekomen wil ze een taxi aanhouden. Er zijn echter weinig taxi's en veel mensen die een taxi willen vanwege de regen. Eindelijk weet ze een taxi aan te houden en deze stopt vlakbij haar.	…	Sanne	is	verontwaardigd	wanneer de taxi vlak voor haar stopt	en iemand haar opzij duwt en gauw instapt
10	Lieke zit met een vriendin op het terras. Het is donderdagmiddag en ze hebben allebei lekker vakantie. Haar vriendin moet naar het toilet, waar het waarschijnlijk stervensdruk zal zijn. Terwijl Lieke alleen zit komt een vriend van haar langsfietsen. Hij vertelt Lieke over een feestje vanavond en moet gelijk verder. Lieke wil wel heen, maar ze heeft eigenlijk al plannen gemaakt met die andere vriendin.	Lieke liegt dat ze een andere afspraak was vergeten zodat ze toch naar het feestje van die vriend kan.	Later die middag springt Lieke gauw onder de douche thuis. Onder het afdrogen bedenkt ze zich dat ze cash geld nodig zal hebben. Dat herinnert haar eraan dat haar salaris één dezer dagen binnen moet komen. Nadat ze zich heeft aangekleed rommelt ze in de la om de random reader te vinden. Eenmaal gevonden logt ze gauw in om haar saldo te checken.	…	Lieke	constateert	nors	dat de salarisadminstratie	weer eens heeft gefaald, nog geen geld
11	Sarah heeft de nachtdienst op de verpleegafdeling. Het is tijd om een ronde te lopen en haar collega en zij doen elk de helft. Sarah controleert alle infusen en kijkt of iedereen goed slaapt. Ze gaat stilletjes van kamer tot kamer. Op één van de laatste kamers ziet ze een portemonnee liggen. Hij is van de oudere man die vanmiddag zijn kleinkinderen met hun rapporten op bezoek had.	Sarah bergt de portemonnee netjes op in het kastje en neemt zich voor de man te waarschuwen voorzichtiger te zijn.	Aan het einde van haar dienst gaat Sarah gauw richting huis. Eenmaal thuis zet ze een kop thee en zet haar laptop aan. Met de laptop in één hand en de thee in de andere loopt ze richting de woonkamer. Ze ziet door het voorraam dat de zon ondertussen op is gekomen. Dan struikelt ze ineens over de drempel en laat de laptop vallen en morst thee.	…	Sarah	constateert	pissig	dat de laptop als ze hem weer aanzet	helemaal niks meer doet, compleet kapot
12	Danny is aan het werk in de werkplaats van zijn fietsenzaak. Hij is bezig een achterwiel te vervangen. Het is halverwege de ochtend en nog lekker rustig. Wanneer de bel gaat maakt Danny snel af wat hij aan het doen was. Hij veegt zijn handen schoon en loopt dan snel naar voren. Er staat een vrouw met een hoofddoek te wachten en het blijkt al gauw dat haar Nederlands niet heel goed is.	Danny neemt rustig de tijd en met de paar woorden Marokkaans die hij kent komen ze er uiteindelijk samen uit.	Later die dag, na de lunch, worden er onderdelen geleverd. Danny tekent voor ontvangst en begint de pakketjes te sorteren. Er is één bepaald onderdeel waar hij al een hele tijd op zit te wachten. Het is een heel speciaal onderdeel voor een luxe mountainbike. Het onderdeel is al twee keer verkeerd geleverd en de klant begint ongeduldig te worden.	…	Danny	voelt	frustratie	dat het binnengekomen onderdeel	weer het verkeerde blijkt, dit kost hem geld
13	Martijn hangt wat rond met zijn vrienden in het park. Ze drinken biertjes en zijn wat aangeschoten en baldadig. Een van Martijns vrienden die meedrinkt begint opmerkingen te maken over mensen die langslopen. Eerst zachtjes, maar later steeds harder. Plotseling loopt een donkere jongen langs het groepje vrienden. Martijns vriend roept een racistische opmerking naar de jongen en daagt hem uit.	Martijn is direct nuchter en eist verontwaardigd dat zijn vriend onmiddelijk zijn excuses aanbiedt aan de jongen.	De volgende dag gaat Martijn naar de elektronicazaak. Hij heeft al een tijd een Ultra HD-tv op het oog en vandaag wil hij hem kopen. De tv is erg duur en Martijn heeft er lang voor gespaard. Hij heeft de achterbank van zijn auto vast naar beneden geklapt om ruimte te maken. Aangekomen bij de winkel vraagt Martijn direct een medewerker naar de tv die hij wil.	…	Martijn	voelt	blijdschap	wanneer in de winkel blijkt dat	de tv zelfs in de aanbieding is en hij geld overhoudt
14	Judith zit op haar fiets voor de supermarkt. Ze is met een groepje van drie vrienden en ze hangen maar wat. Terwijl ze wat dom ouwehoeren ziet Judith een hardloper aankomen. De loper is hevig bezweet en het is hem aan te zien dat hij tot het uiterste gaat. Judith en haar vrienden staan pal in het pad van de hardloper. Judith haar vrienden doen een paar stappen opzij om plaats te maken.	Judith zet haar fiets lekker nog dwarser en als de hardloper uitwijkt, rolt Judith expres ook een stukje die kant op.	Later die dag loopt Judith van een terrasje door de stad naar huis. Het is nog redelijk vroeg, maar het begint al te schemeren. De zomer loopt ten einde, maar de temperatuur is nog lekker. Judith steekt de brug over en merkt ineens dat haar portemonnee niet in haar zak zit. Ze zou zweren dat ze hem net nog had en draait zich om om te kijken of hij ergens ligt.	…	Judith	voelt	woede	als ze zich omdraait en	ziet hoe een man wegrent met haar portemonnee
15	Adriana loopt door het bos op een zondagochtend. Het is vroeg in de herfst en de bladeren beginnen net te verkleuren. Ze heeft onderweg koffie gehaald bij de enige koffiezaak die op een zondag-ochtend open is. Terwijl ze wandelt, neemt ze af en toe een slokje. Ze loopt graag in dit bos, het is redelijk groot en ze komt doordeweeks niet veel buiten. Halverwege haar gewone route heeft ze haar koffie op.	Adriana houdt netjes de beker bij zich om op de parkeerplaats weg te gooien, zo blijft het bos voor iedereen schoon.	Een uur later loopt Adriana nog steeds in het bos. Ze is ondertussen een heel eind bij de auto vandaan, maar ze is nog lang niet moe. Dan komt haar voet ineens in een gat terecht en verzwikt ze haar enkel. Ze voelt een scherpe pijn en valt om omdat ze niet meer op dat been kan staan. De pijn bonst stug door in haar enkel en ze kan niet anders dan langs het pad blijven zitten.	…	Adriana	voelt	blijdschap	als er even later iemand langskomt	die haar vriendelijk naar de auto helpt
16	Kelly loopt door het park op weg naar een feestje. Het schemert al, maar het is nog redelijk licht. Het is een warme dag geweest en er liggen nog mensen op handdoeken op het gras. Kelly pakt haar telefoon om op te zoeken waar ze precies moet zijn. Dan ziet ze vanuit haar ooghoek een portemonnee liggen. Er is niemand te bekennen en Kelly pakt de portemonnee op die vol geld blijkt te zitten.	Kelly checkt of er iets met een naam en adres in zit en besluit de portemonnee netjes naar de politie te brengen.	Kelly vervolgt haar weg naar het feestje en gaat daarna nog even de kroeg in. De volgende ochtend komt ze redelijk op tijd uit bed. Gelukkig heeft ze geen kater want ze moet nog voor haar studie aan de slag vandaag. Ze zet de laptop aan en gaat dan eerst koffie maken. Als ze terug komt opent ze haar mail en ziet een mailtje van de studieadviseur.	…	Kelly	is	furieus	als ze in de email leest dat ze	niet mag herkansen en niet kan afstuderen
17	Tim stapt 's middags in de tram naar huis. Hij checkt in met zijn pas en loopt door naar achter om een plaatsje te zoeken. Achter Tim stapt ook nog een vrouw in, ze loopt achter hem aan. Ze is hoogzwanger en volgt hem verder de tram in. Tim ziet achterin nog één vrije zitplaats en stevent er op af. Voordat Tim de zitplaats bereikt begint de tram schokkend weer te rijden.	Tim neemt plaats en blijft zitten ook al wordt de zwangere vrouw vlak naast hem alle kanten op geslingerd.	Eenmaal thuis neemt Tim de stapel post door. Er zit een zoveelste brief bij van de belastingdienst. Hij is al maanden bezig om zijn aanslag gecorrigeerd te krijgen. De belastingdienst heeft een fout gemaakt en weigert die te herstellen. Hij hoopt dat het eindelijk is opgelost, hij heeft er genoeg van.	…	Tim	is	gefrustreerd	wanneer blijkt dat zijn aanslag	nog steeds niet klopt
18	David staat op de steiger op het punt om te beginnen te schuren. Het is halverwege de ochtend en het is al flink heet. Het is de derde week van deze klus en ze zijn met het laatste stuk bezig. Hij is alleen aan het werk aan deze kant, zijn collega's zijn achter bezig. Hij ziet door het raam een vrouw achter een bureau zitten. Haar telefoon gaat en ze neemt aan en begint een heel gesprek.	David gaat lekker schuren, en als ze boos wordt maakt hij een suggestief gebaar naar zijn kruis en gaat door.	Na de lunch klimt David weer de steiger op. Hij gaat verder met het schuurwerk, ondertussen is hij bij het laatste raam. Het werk is goed opgeschoten ondanks de hitte. David werkt gestaag door en al gauw is ook het laatste kozijn geschuurd. David zet de machine uit en terwijl hij zijn bezwete voorhoofd afveegt glipt de schuurmachine uit zijn vingers.	…	David	is	chagrijnig	als hij ziet hoe het apparaat	van de steiger valt en op de tegels kapot gaat
19	Michael gaat met zijn vrienden naar een voetbalwedstrijd. Hun club speelt uit tegen de aartsrivaal. De sfeer is gespannen, want de winnaar heeft grote kans kampioen te worden. Ze komen in een vak met medesupporters te zitten. Voor aanvang wordt er om een minuut stilte gevraagd voor een overleden speler van de tegenpartij. Het is rumoerig in het vak en ook Michaels vrienden praten en lachen.	Michael maant zijn vrienden aan stil te zijn en tot Michaels genoegen volgt de rest van het vak hun voorbeeld.	Een week later gaat Michael op vakantie. Hij vliegt 's ochtends vroeg naar Cyprus, waar hij een week in de zon wil liggen. Op internet heeft hij een goedkoop appartementje geboekt. De foto's van het appartement waren wat onduidelijk, maar Michael kon de aanbieding niet laten gaan. Eenmaal geland op Cyprus huurt Michael een auto en rijdt naar het appartement.	…	Michael	is	verheugd	wanneer bij aankomst blijkt dat het	nog mooier is dan gedacht en het weer schitterend is
20	Wendy komt wat laat aan bij een feestje van haar broer. Het is een soort housewarming. Haar broer heeft zowel vrienden en familie als collega's uitgenodigd. Het is een flink groot feest, maar Wendy haar broer had geen zin om drie keer iets te organiseren. Wendy wordt gespot door haar broer en her en der voorgesteld. Dan stelt haar broer haar voor aan een collega die een mismaakte hand heeft.	Wendy trekt een gezicht en weigert de uitgestoken hand, de man is duidelijk gekwetst, maar dat boeit haar niet.	Later op de avond staat Wendy met wat familieleden te kletsen. Een aantal neven en een tante zijn ook aanwezig. Wendy heeft ze al een poos niet gezien en ze praat ze bij over haar leven. Wanneer Wendy even naar de wc gaat en weer terugkomt, treft ze iedereen lachend aan. Ze stoppen abrupt als ze er weer bij komt staan en Wendy kijkt ze vragend aan.	…	Wendy	is	kwaad	wanneer ze even later	merkt dat ze haar belachelijk maakten
21	Laura komt pas om een uur of acht uit een vergadering. Ze is moe, maar tevreden over de uitkomst. Ze besluit naar huis te lopen omdat het een mooie zomeravond is. Eenmaal uit de drukte van het centrum is het heerlijk rustig op straat. Een paar straten van haar huis ziet Laura het portier van een auto op een kier staan. Ze kijkt rond of er iemand in de buurt is, maar er is niemand.	Laura denkt even na en duikt dan gauw met haar kop in de auto, haalt de autoradio eruit en loopt gauw verder.	De volgende ochtend staat Laura vroeg op en gaat naar haar werk. Ze is nieuwsgierig naar de uitkomst van een subsidieaanvraag die ze heeft ingediend. Bij de vergadering van gisteren heeft ze geprobeerd de laatste twijfelaars over te halen. Het leek erop dat de aanvraag ingewilligd ging worden. Laura probeert gewoon te werken terwijl ze op nieuws wacht.	…	Laura	is	trots	wanneer blijkt dat haar aanvraag	is goedgekeurd en haar project kan starten
22	Jasper gaat naar de schouwburg voor een toneelvoorstelling. Het is druk, want het is een mooi stuk en er spelen veel bekende acteurs mee. Voor de kassa staat een enorme rij, waar Jasper achter aansluit. Plots hoort Jasper iemand verderop zijn naam roepen. Verderop in de rij staat een vriend van Jasper naar hem te zwaaien. Hij wenkt Jasper en gebaart dat hij bij hem in de rij moet komen staan.	Jasper wil niet voordringen en gebaart naar zijn vriend dat hij hem binnen wel ziet en wacht op zijn beurt.	Na de voorstelling loopt Jasper naar de garderobe. Hij had een jas en een paraplu meegenomen, omdat er stevige buien waren voorspeld. Bij de garderobe staat personeel dat de jassen en dergelijk ophaalt. Jasper haalt het garderobenummer tevoorschijn dat hij had gekregen. Hij geeft het nummer aan het garderobepersoneel, dat op zoek gaat naar zijn spullen.	…	Jasper	voelt	blijdschap	wanneer het garderobepersoneel	zijn spullen snel vindt en hij droog thuiskomt
23	Thomas zit in de kroeg met vrienden. Het is een mannenavondje en ze drinken er lustig op los. Thomas heeft vakantie en zijn vriendin is een week bij haar ouders. Hij mist haar wel, maar aan de andere kant is wat vrijheid ook wel fijn. Als iedereen besluit naar huis te gaan blijft Thomas nog even hangen. Hij raakt aan de praat met een mooie vrouw aan de bar die hem wel ziet zitten.	Thomas verzwijgt dat hij een vriendin heeft en gaat later met haar mee naar huis, zijn vriendin hoeft niks te weten.	De volgende dag sleept Thomas zich rond twee uur naar de supermarkt. Hij heeft niks meer in huis en ondanks een lichte kater toch honger. Wanneer hij uit de supermarkt komt zet hij de tassen even neer. Hij heeft gelijk maar grote boodschappen gedaan en het weegt heel wat. Wanneer hij bij zijn flat aankomt, ziet hij iemand vlak voor zich de deur openen.	…	Thomas	is	furieus	als de man omkijkt, even wacht en	dan de deur in zijn gezicht dicht laat vallen
24	Bram staat 's ochtends vroeg op en komt gelijk in actie. Hij gaat vandaag weer naar huis en hij wil nog winkelen voor hij vliegt. Hij heeft in het huis van vrienden van vrienden gezeten. Hij kent ze verder niet, maar ze hebben hem gratis laten logeren. Bram komt de keuken binnen en trekt de koelkast open. Het lampje springt niet aan en Bram komt er achter dat er een stop is gesprongen.	Bram besluit het te negeren en gaat lekker winkelen terwijl de vriezer langzaam ontdooit en overal water lekt.	Later die dag rent Bram beladen met al zijn bagage naar de trein. Hij springt net op tijd in de trein richting het vliegveld. De trein vertrekt op tijd maar tien minuten later staan ze ineens stil tussen de weilanden. Bram begint zich zorgen te maken dat hij te laat gaat komen. Een uur later dan gepland komt de trein eindelijk aan op Schiphol.	…	Bram	is	opgelucht	als hij aankomt op het vliegveld en	blijkt dat zijn vlucht ook vertraagd is

25	Nina rijdt met de auto door de binnenstad. Het is druk op de weg en er komt een onoverzichtelijke kruising aan. Fietsers en auto's moeten hier de trambaan over vanwege werkzaamheden. Een oudere man op de fiets wankelt onzeker de trambaan op. Het is duidelijk dat de man in de war is en niet goed weet wat hij moet doen. De man komt bijna volledig tot stilstand recht voor Nina haar auto.	Nina toetert kwaad en scheurt rakelings langs de oude man, in haar spiegel ziet Nina de man omvallen.	Drie straten verder slaat Nina rechstaf richting haar appartement. Ze parkeert een straat verderop waar bijna altijd wel plaats is. Nina zet de motor af en graait haar tas van de achterbank. Nadat Nina de auto op slot heeft gedaan zwaait ze haar tas over haar schouder. Bij de entree van haar gebouw ziet ze vanuit haar ooghoek iemand aan komen lopen.	…	Nina	is	uitzinnnig	wanneer de jongen op haar afloopt	en haar sleutels gevonden blijkt te hebben
26	Cynthia is op het werk de penningmeester van de personeelsvereniging. Het is een initiatief van en voor werknemers en ze organiseren af en toe gezellige uitjes. Daarnaast verwelkomen ze nieuwe collega's met een cadeautje. Eén keer per jaar innen ze daarvoor vrijwillig contributie van iedereen. Cynthia heeft vorige week een mailtje uitgestuurd met het verzoek te betalen. Ondertussen heeft bijna iedereen het geld overgemaakt.	Cynthia stopt het geld in de kluis, dan maakt ze een rapport op en zorgt dat iedereen het kan inzien en controleren.	De week daarop moet Cynthia naar de dokter. Ze is al een poosje erg vermoeid en heeft weinig eetlust. Ze heeft daarom vorige week bij de dokter bloed laten prikken. De doktersassistente belde gisteren dat ze de volgende ochtend langs moest komen. Het is standaard procedure dit soort nieuws niet aan de telefoon te vertellen, maar ze is toch nerveus.	…	Cynthia	is	kwaad	als uiteindelijk blijkt dat	de resultaten zoek zijn en ze opnieuw moet
27	Frank loopt richting de ingang van het strandpaviljoen. Hij heeft met vrienden afgesproken, maar is aan de vroege kant. Frank loopt blootsvoets over het zand door de branding. Ter hoogte van het paviljoen begint hij aan de klim het strand op. De eerste trede van de trap naar boven is vrij hoog boven het zand. De stap is zo groot dat een jochie van een jaar of vier de trede niet op komt.	Frank dendert langs het joch en zorgt ervoor dat deze zijn balans verliest, omvalt en begint te huilen.	Frank zoekt binnen een tafel waar iedereen kan zitten en bestelt alvast wat te drinken. Een half uurtje later is iedereen aanwezig en bestellen ze wat te eten. Frank bestelt een biefstuk, medium rare met friet. Wanneer de biefstuk arriveert, blijkt hij bijna well-done te zijn. Frank roept de ober terug om het probleem uit te leggen en laat de biefstuk zien.	…	Frank	is	ziedend	als de ober reageert op een	boze manier en net doet of hij zeurt
28	Remco zit in de auto, op weg naar zijn moeder. Hij is de stad nog niet uit en het spoelt van de regen. Het regent onophoudelijk sinds gistermiddag en er hebben zich flinke plassen gevormd. Op sommige plekken staat de straat volledig blank. Er zijn weinig auto's en al helemaal weinig fietsers en voetgangers op straat. Remco stevent op een gigantische plas af en ziet iemand op de stoep lopen.	Remco gaat langzamer rijden en wijkt ietsje uit om te voorkomen dat hij de wandelaar zeiknat spettert.	Eenmaal buiten de stad schiet het lekker op op de snelweg. Het blijft rustig op de weg en Remco geniet van het landschap en de rit. Hij heeft de radio hard aan staan en zingt luidkeels mee. Wanneer hij naar het dashboard kijkt om de zender bij te stellen ziet hij een lampje. Hij is vergeten te tanken en de benzine was al heel lang echt bijna op.	…	Remco	is	gefrustreerd	als hij na een paar minuten rijden	geen bezine meer heeft en stil komt te staan
29	Maarten is als enige van zijn team op werkbezoek in het buitenland. De bedoeling is om een kijkje te nemen bij een vergelijkbaar team op het hoofdkantoor. Maarten weet dat dit team op hun projecten uit is. Ze willen maar wat graag dat Maartens afdeling moet sluiten. De teamleider van het rivaliserende team wil een één op één gesprek met Maarten. Hij nodigt Maarten uit om bij hen te komen werken als hij zijn team saboteert.	Maarten overweegt dat geen moment en weigert, hij zou nooit zijn collega's thuis verraden voor eigenbelang.	Later die week vliegt Maarten terug naar huis. Het is een lange vlucht en hij heeft onderweg veel om over na te denken. Als het vliegtuig eindelijk landt wacht hij ongeduldig op zijn bagage. Het is hartstikke vroeg en hij wil zijn vrouw verrassen met een ontbijt op bed. Onderweg naar huis koopt hij allerlei lekkere dingen en gaat dan snel door naar huis.	…	Maarten	voelt	razernij	als hij eenmaal thuiskomt	en zijn vrouw met ander in bed aantreft
30	Jordy loopt over het strand terug naar zijn handdoek. Onderweg komt hij langs een kraampje dat friet en dergelijke verkoopt. Het is er niet druk, het is überhaupt niet druk vandaag. Ondanks dat het een mooie dag is, is er maar een tiental mensen. Jordy loopt voorbij een meisje met een mitella dat probeert een flesje cola te openen. Het wil niet echt lukken met één hand en ze kijkt gefrustreerd.	Jordy stopt en helpt haar even, het is een kleine moeite en hij zou zelf ook willen dat iemand hem hielp.	Jordy gaat nog een stukje zwemmen in de aanzienlijke golfslag. Daarna gaat hij liggen drogen in de zon en leest zijn boek. Hij gaat helemaal op in het verhaal en de tijd verstrijkt. Op een gegeven moment bedenkt hij zich dat de laatste bus vrij vroeg gaat op zondag. Hij checkt hoe laat het is en bedenkt zich dat hij zal moeten opschieten om de bus te halen.	…	Jordy	is	pissig	als de buschauffeur hem ziet	en gewoon doorrijdt en hem daar laat staan
31	Ruben zit op de bank met zijn laptop op schoot wanneer de bel gaat. Hij zet de laptop op de bank naast zich en staat op. Het is een pakketje voor iemand anders in het gebouw. Ruben neemt het aan en ziet op de pakbon staan dat het een geluidsinstallatie is. Ruben gaat weer verder met waar hij mee bezig was. Later die dag gaat de deurbel en het is de buurvrouw die haar pakketje komt halen.	Ruben pakt de zware doos voor haar en biedt aan om hem voor haar te tillen, wat ze dankbaar accepteert.	De volgende dag is Ruben onderweg naar huis van de supermarkt. Hij heeft twee volle en zware tassen boodschappen. Bij de stoplichten zet hij de tassen naast zich neer en wacht op groen. Wanneer het groen wordt pakt hij de tassen op en haast zich naar de overkant. Bijna aan de overkant struikelt Ruben over de stoeprand en valt tegen de man voor hem aan.	…	Ruben	wordt	witheet	wanneer de man voor hem	hem een duw geeft en hij dus keihard valt
32	Dennis stapt de lift in in het ziekenhuis. De vrouw van zijn broer is gisteren bevallen en hij gaat op kraamvisite. Hij heeft een grote knuffel onder zijn arm en een bos bloemen. Hij stapt op de begane grond in en moet op de vijfde verdieping zijn. Op de derde verdieping stopt de lift en stapt een vrouw in. De vrouw is kaal en mager, ze heeft wallen onder haar ogen en een grauwe huid.	Dennis gaat zover mogelijk bij haar vandaan staan en staart naar haar, ze voelt zich zichtbaar ongemakkelijk.	Wanneer hij eenmaal zijn jongste neefje heeft bekeken gaat Dennis weer. Hij is met de bus, die stopt vlak bij hem voor de deur. Hij is binnen een half uurtje thuis en maakt wat te eten. In de keuken ziet hij op het bord zijn to-do lijstje en herinnert zich dat de tuin een wildernis is. Hij werpt een snelle blik naar buiten om te zien hoe erg het is.	…	Dennis	is	kwaad	als hij de tuin inkijkt en ziet dat	de buren hun tuinafval in zijn tuin hebben gegooid
33	Saskia is vrijwilliger bij een grote manege en vandaag hebben ze open dag. Vanuit de hele omgeving komen ouders en vooral kinderen naar de manege. Er worden rijlessen en workshops over paardenverzorging gegeven. Saskia heeft de hele ochtend workshops gegeven. 's Middags laat ze jonge kinderen ritjes maken in de bak. Wanneer Saskia zich omdraait naar het volgende kind ziet ze meteen dat het downsyndroom heeft.	Saskia weigert om haar een ritje te laten maken, het idee om haar aan te moeten raken geeft haar huiveringen.	De volgende ochtend is Saskia vroeg op haar normale werk. Ze werkt bij een groot accountantsbedrijf. Ze moet om 9:00 bij de baas komen en ze weet niet precies waarvoor. Ze heeft er slecht van geslapen vannacht terwijl ze probeerde te verzinnen waar het over zal gaan. Wanneer het eenmaal negen uur is en Saskia bij de baas zit steekt hij gelijk van wal.	…	Saskia	is	laaiend	wanneer de baas in het gesprek	haar onterecht dingen aanrekent en ontslaat
34	Kim loopt 's middags door de supermarkt. Op haar lijstje staat van alles om zelf een verjaardagstaart te maken. Wanneer ze alles heeft gevonden gaat ze naar de kassa. Voor haar in het gangpad loopt een oudere vrouw. Ze is duidelijk ook onderweg naar de kassa, maar ze loopt minder snel dan Kim. Het gangpad is niet zo heel breed en er komt iemand van de andere kant aanlopen.	Kim heeft geen zin om te wachten en haalt haar snel in en sluit lekker vlak voor haar neus aan in de rij.	Later die dag is ze bezig de taart te bakken, hij staat in de oven. Ze viert vandaag haar verjaardag en heeft iedereen eigengemaakte taart beloofd. Kim staat niet bepaald bekend om haar culinaire hoogstandjes. Een aantal van haar vrienden en familie reageerde dan ook nogal sceptisch. Het is nu een kwestie van eer geworden dat deze taart goed lukt.	…	Kim	is	blij	wanneer de cake uiteindelijk	perfect uit de oven komt
35	Joris loopt 's ochtend over de markt richting station. Hij heeft zijn oordopjes in en luistert naar muziek. De marktkooplui zijn in de weer met het opbouwen van hun kramen. De klinkers zijn wit uitgeslagen van de vorst van vannacht. Een man passeert voor Joris met een stapel kisten vol mandarijnen in zijn armen. De bovenste wankelt wat en even later ligt de straat bezaaid met mandarijnen.	Joris stampt expres twee mandarijnen kapot, schopt een derde tegen een andere kraam en loopt dan snel door.	Later die ochtend komt Joris terug van de lunch. Hij gaat weer achter zijn bureau zitten en kijkt naar zijn lijstje met taken voor vandaag. Hij is lekker opgeschoten vanochtend, maar kan nu eigenlijk niet verder. Hij wacht op bericht van een afdeling in het buitenland. Hij heeft ze gemaild of ze op willen schieten omdat hij niet verder kan zonder de info.	…	Joris	is	woest	als hij een mailtje krijgt met daarin	informatie die hij 3 maanden geleden nodig had
36	Tom rent snel terug richting het eigen doel na de gefaalde aanval. De tegenstander heeft net de bal terugveroverd. Zijn team heeft moeite met terugschakelen naar verdedigen. Over de flank krijgt de spits van de tegenstander een geweldige lange pass. Tom sprint om hem af te snijden op weg naar het doel. Tom zet een tackle in en beseft zich meteen dat hij veel te hard in heeft gezet.	Tom strekt zijn been expres nog verder en met genoegen voelt hij de enkel van de tegenstander knakken.	De bal komt bij de keeper terecht en die speelt Tom aan. Tom neemt de bal aan en begint een soloactie langs de lijn. Hij passeert twee man en een derde komt veel te laat aanrennen. Tom trekt naar binnen richting de zestien meter van de tegenstander. Zijn teamgenoten zijn nog niet mee naar voren en dus legt Tom zelf aan om uit te halen.	…	Tom	is	trots	als hij aanlegt om te schieten	en de keeper passeert en scoort
37	Sandra krijgt haar schoonouders vanavond op bezoek en ze blijven eten. Ze wil indruk op hen maken met haar kookkunsten. Ze heeft alleen nog niks in huis en rijdt daarom naar de supermarkt. Sandra parkeert haar auto op de parkeerplaats en loopt naar de ingang van de supermarkt. Bij de ingang staat een man die overduidelijk dakloos is. De man loopt op Sandra af en vraagt om wat kleingeld.	Sandra vist wat kleingeld uit haar broekzak en geeft het aan de man, die er overduidelijk blij mee is.	Wanneer Sandra de boodschappen heeft gekocht, rijdt ze weer naar huis. Thuis aangekomen loopt ze met haar boodschappen naar de voordeur. Sandra kan haar voordeursleutel niet vinden. Op dat moment bedenkt ze zich dat ze zonder sleutel de deur achter zich dicht heeft gedaan. Snel loopt ze naar het huis van haar buren, die een reservesleutel hebben.	…	Sandra	is	opgelucht	als ze bij de buren aanbelt en	die thuis blijken te zijn in ze dus haar huis in kan
38	Koen is op stap met een groepje vrienden. Ze kennen elkaar nog van hun studietijd en spreken af en toe af. Koen heeft al een aantal biertjes op en de rest heeft hem ook aardig zitten. Op een gegeven moment besluiten ze naar de volgende kroeg te gaan. Voor de ingang van de kroeg staat een groepje kerels te roken. Één van Koen zijn vrienden loopt op ze af en begint ruzie te zoeken.	Koen bedenkt zich niet en haalt uit naar één van de andere jongens in het groepje, die gelijk plat gaat.	Daarna is de avond snel afgelopen en gaat Koen naar zijn fiets. Zijn fiets staat helemaal aan de andere kant van het centrum. Het is ondertussen drie uur geweest en hij wil niets liever dan slapen. Het schiet Koen ineens te binnen dat hij zijn fiets helemaal niet op slot heeft gezet. Hij graaft in zijn zakken en kan de sleutel nergens vinden.	…	Koen	is	gelukkig	als hij aankomt waar hij zijn fiets had staan	en ziet dat deze er gewoon nog staat
39	Inge loopt 's avonds naar het huis van haar ouders. Ze is bij een vriend geweest die ze niet had gezien sinds ze is verhuisd. Het is schemerig en het is een eind lopen, maar dat vindt Inge niet erg. Het is fijn om weer door bekende straten te slenteren. Verderop ziet Inge een gedaante op vier poten uit de bosjes komen. Het is een gewonde hond die zacht jankt en naar haar toe loopt.	Inge bedenkt zich niet en geeft het beest een flinke schop en de hond verdwijnt jankend weer de bosjes in.	Eenmaal thuis na het incident met de hond gaat Inge direct naar bed. De volgende dag staat ze vroeg op om nog wat mensen op te zoeken. Ze leent de auto van haar ouders omdat sommigen buiten de stad wonen. Halverwege, midden tussen de weilanden, houdt de motor er mee op. Na 20 minuten wachten langs de weg komt er eindelijk een andere auto aan.	…	Inge	is	uitgelaten	wanneer de andere automobilist	stopt en haar auto kan repareren
40	Anouk zit alvast in de vergaderruimte waar ze straks overleg heeft. Ze neemt de documenten die ze heeft voorbereid nog eens door. In de excel sheets van de begroting spot ze ineens een grove fout. Deze fout betekent dat het project veel meer gaat kosten dan ze had gezegd. Anouk heeft weinig zin om dit op te moeten biechten. Voordat ze echt na heeft kunnen denken komen haar bazen binnen.	Anouk begint de vergadering met de fout eerlijk toe te geven en gelukkig wordt haar eerlijkheid gewaardeerd.	De vergadering verloopt verder prima en Anouk gaat door met haar dag. Tegen een uur of acht is ze thuis en haalt de post op. Eenmaal binnen ziet ze een brief van het elektriciteitsbedrijf. Het is de jaarafrekening en dat was vorig jaar een dure grap. Anouk heeft toen het voorschot bij laten stellen en hopelijk hoeft ze nu niet bij te betalen.	…	Anouk	is	driftig	als uit de brief blijkt dat ze	wederom een groot bedrag moet bijbetalen
41	Jesse komt 's middags rond half vier aanrijden bij de school. Hij parkeert in de rij wachtende ouders. Er zijn nog geen kinderen op het plein en hij wacht rustig. Hij is zo verzonken in gedachten dat hij niet merkt dat de kinderen naar buiten komen. Hij is dan ook verrast wanneer zijn dochtertje plots achterin klimt. Ze heeft een tekening gemaakt die ze vol trots laat zien en aan hem wil geven.	Jesse prijst haar tekening uitbundig en zegt dat ze met zo'n mooie tekening wel een ijsje verdiend heeft.	Later die avond als zijn dochtertje al op bed ligt kijkt Jesse voetbal. Hij heeft geld gezet op de uitkomst van deze wedstrijd. Het was een flink bedrag en hij wil graag winnen, want hij kon het geld eigenlijk niet missen. Vlak voor tijd staat het 1-1 en hij heeft geld gezet op 2-1 winst. Hij kijkt gespannen toe hoe zijn team een laatste aanval inzet.	…	Jesse	is	woest	wanneer vlak voor het einde	de tegenpartij scoort en hij zijn geld kwijt is
42	Kevin zit 's middags in de trein op weg naar Groningen. Het is ontzettend rustig in de trein sinds Zwolle. Er zitten naast Kevin maar vier mensen in de hele coupé. Er zit een man in de vierzits aan de overzijde van het gangpad een boek te lezen. Wanneer de trein weer stopt staat de man op om uit te stappen. Ineens ziet Kevin dat de man een tas onder de bank heeft laten staan.	Kevin houdt zich lekker stil en wacht tot de trein weer rijdt en spit dan de tas door voor waardevolle spullen.	Eenmaal in Groningen aangekomen pakt Kevin de bus richting Oranjewijk. Hij logeert bij een vriendin en ze gaan naar een concert in de Oosterpoort vandaag. De bus doet er iets langer over dan normaal, maar Kevin heeft geen haast. Hij stapt uit en loopt de laatste vijf minuten naar haar huis. Vlak voor hij aanbelt, gaat zijn telefoon en hij neemt op.	…	Kevin	voelt	vreugde	wanneer hij te horen krijgt	dat zijn bod op een huis is geaccepteerd
43	Julia zit op haar plek achter de receptie, de telefoon staat roodgloeiend. Ze handelt het ene na het andere telefoontje af. Voordat Julia het weet is de ochtend alweer voorbij. Na de lunch is het iets minder druk. Dan gaat de telefoon weer, het is iemand voor de man van accounting. De man heeft de laatste tijd wat steken laten vallen en ook nu weer belt er iemand met een probleem.	Julia loopt snel even naar de man toe om hem de kans te geven de fout te herstellen voordat iemand anders het merkt.	Later die week is Julia aan het winkelen. Ze zoekt een specifiek computerspelletje voor de verjaardag van haar broertje. Ze heeft hem beloofd dat hij deze morgen voor zijn verjaardag krijgt. Julia heeft echter weinig tijd gehad om te winkelen de laatste tijd. Eindelijk vind ze één kopie van het spelletje, maar een andere klant ziet hem tegelijkertijd.	…	Julia	is	geïrriteerd	als de andere klant het spelletje	snel afrekent en haar compleet negeert
44	Larissa is lerares op een basisschool. Ze geeft les aan de bovenbouw, groep vijf en zes. Op een woensdagochtend heeft ze pleinwacht tijdens de pauze. Ze loopt grote rondes over het plein en houdt alles goed in de gaten. Af en toe spreekt ze een leerling aan, maar verder is het rustig. Dan ziet ze een stel leerlingen uit groep 8 aan de rand van het plein rommel staan gooien naar een vrouw die langsloopt.	Larissa roept de jongens meteen tot de orde, zorgt dat ze hun excuses aanbieden en laat ze 's middags nablijven.	Later die week gaat Larissa naar de bank voor een afspraak. Ze wil haar hypotheek oversluiten en snapt niet veel van financiën, dus wil ze persoonlijk advies. Ze loopt de bank binnen en gaat naar de balie om zich te melden. De receptioniste zegt haar plaats te nemen, ze komen haar zo halen. Larissa gaat zitten en leest een tijdschrift terwijl ze rustig wacht.	…	Larissa	is	gerustgesteld	als ze twee uur later naar buiten loopt	met een nieuwe en zeer gunstige hypotheek
45	Hendrik zit 's ochtends al vroeg op het kantoor. Hij is druk bezig met het tentamen schrijven dat zijn studenten volgende week moeten maken. Terwijl hij daar mee bezig is wordt er op de deur geklopt. Het is één van de studentes uit zijn werkgroep, een knappe verschijning. Het is al gauw duidelijk dat ze probeert Hendrik te verleiden om haar het tentamen in te laten zien. Ze zinspeelt op hoe dankbaar ze zou zijn.	Hendrik vertelt haar in niet mis te verstane bewoordingen dat hij daar niet voor in is en dat ze weg moet gaan.	Later die dag moet Hendrik college geven in een gebouw buiten de binnenstad. Hij pakt de fiets daarheen en trapt stevig door. Op een gegeven moment fietst hij op een drukke kruising af en stopt hij voor het licht. Even later springt het op groen en Hendrik steekt over. Halverwege de kruising vliegt de ketting er ineens af en staat hij midden op de kruising stil.	…	Hendrik	is	dankbaar	als de auto's die nu groen hebben	rustig wachten en hem vriendelijk de tijd geven
46	Mark loopt snel over het plein richting het station. Hij gaat vandaag naar Haarlem voor een sollicitatiegesprek. Hij heeft zich goed voorbereid en is ruim op tijd van huis gegaan. Vlakbij de ingang naar de stationshal staat een zwerver te bedelen. Wanneer Mark langsloopt, houdt hij een papieren bekertje omhoog met wat kleingeld erin. De man kijkt hoopvol hoe Mark in zijn broekzak graaft.	Mark vist wat snoeppapiertjes op en gooit deze in het bekertje van de zwerver, laat hem werk gaan zoeken.	Hij loopt snel door en kijkt op het bord welk spoor hij moet hebben. Hij ziet dat er over 2 minuten een trein gaat en zet het op een rennen. Als hij deze trein kan halen, dan komt hij sowieso op tijd. Het is druk in de tunnel naar de sporen en Mark moet steeds uitwijken. Eindelijk bereikt hij de trap en sprint met drie treden per keer naar boven.	…	Mark	is	dankbaar	als de conducteur hem ziet rennen	en vriendelijk even op hem wacht
47	Myrthe werkt op de marketingafdeling van een groot bedrijf. Op dezelfde afdeling werkt haar beste vriendin. Samen werken ze al jaren samen en ze zijn een hecht team. Op een ochtend wordt Myrthes vriendin naar het kantoor van de baas geroepen. Myrthe weet niet waar het over gaat, maar ze is wel nieuwsgierig. Later die dag hoort Myrthe dat haar vriendin een promotie heeft gekregen en zij niet.	Myrthe is teleurgesteld, maar blij te hebben bijgedragen aan het succes van haar vriendin en feliciteert haar.	Later die week heeft Myrthe een zeldzame vrije dag. Ze besluit de meubelzaken bij langs te gaan voor nieuwe meubels. Bij IKEA ziet ze een leuke garderobekast, die perfect past in haar slaapkamer. Myrthe aarzelt geen moment en koopt de kast meteen en gaat weer richting huis. Myrthe begint thuis meteen enthousiast het bouwpakket in elkaar te zetten.	…	Myrthe	is	tevreden	als bij het monteren blijkt	dat de kast perfect past waar ze hem wilde
48	Elise zit in de bus op weg naar de film. Ze heeft haar oordopjes in en zoekt de filmtijden nog eens op. De bus is halverwege en ze sms't haar vrienden dat ze eraan komt. Elise verandert de muziek naar iets stevigers en zet het harder. Er zitten weinig mensen in de bus behalve twee oudere dames voor haar. Één van hen draait zich om om te zeggen dat haar muziek wel heel hard staat.	Elise zet haar muziek lekker nog harder en maakt een obsceen gebaar naar haar, waar bemoeit die ouwe tang zich mee?	Twee haltes verder stapt Elise uit en loopt richting bioscoop. Bij de ingang ontmoet ze haar vrienden en ze gaan gauw naar de zaal. Na de film gaan ze nog even gezellig wat drinken. Elise bestelt een biertje, maar bedenkt zich later en loopt naar de bar om iets anders te bestellen. Ze zegt tegen de serveerster dat ze toch liever een witte wijn heeft.	…	Elise	wordt	chagrijnig	als de serveerster reageert	op een norse manier en haar boos aankijkt
49	Eva loopt naar huis van de supermarkt aan de overkant van de straat. Ze heeft één tas met boodschappen, maar die weegt niet zoveel. Met haar vrije hand opent ze de zware portiekdeur van de flat. Achter haar loopt een vrouw met twee tassen, ze heeft moeite met het gewicht. Eva herkent haar en weet dat ze ook de flat in moet. Ze stopt even in de deuropening en denkt na wat te doen.	Ze besluit lekker toch gauw door te lopen en laat de deur mooi in het gezicht van de vrouw dicht vallen.	Later die dag wil Eva een afspraak maken bij de dokter. Ze zoekt het nummer op en pakt de telefoon. Ze wil al een hele tijd een afspraak maken vanwege een zere knie. Eva is echter zo druk dat ze vaak geen zin heeft om te bellen, het wachten duurt altijd zo lang. Ze moet eigenlijk al weer de deur uit, maar wil toch even snel bellen.	…	Eva	is	verrast	wanneer de telefoon over gaat	en ze gelijk de assistente krijgt

50	Niels is 's ochtends vroeg als eerste op kantoor. Het is verder muisstil op de afdeling. Na een half uurtje heeft Niels al flink wat werk verzet, dit is zijn favoriete deel van de dag. Hij legt de laatste hand aan een document en streept hem van zijn lijstje. Vervolgens gaat hij koffie halen en komt de eerste collega tegen. Niels kent haar vaag en weet toevallig dat ze sindskort op dieet is.	Niels zegt haar goedemorgen en complimenteert haar met het resultaat van het dieet en hoe goed ze eruitziet.	Niels werkt rustig door tot de lunch en daarna heeft hij een vergadering. Zijn team presenteert vandaag de resultaten van een project. Het project is een enorm succes geworden voor het bedrijf. Niels presenteert vandaag zelf niet, maar hij weet dat hij een integraal onderdeel van dit succes is. De belangrijkste ideeën voor het nieuwe programma kwamen van hem.	…	Niels	voelt	razernij	wanneer zijn manager in de meeting	liegt en alle eer voorzichzelf opstrijkt
51	Emma is op weg naar de sportschool op een doordeweekse avond. Het begint net wat te schemeren en het regent gestaag. Ze steekt over bij de stoplichten en gaat de hoek om. Aan het eind van de straat ziet ze mensen in en uit lopen bij de sportschool. Aan de overkant van de straat fietst een oudere vrouw de andere kant op. Emma ziet hoe ze plots begint te slingeren en met fiets en al omvalt.	Emma kijkt even en haalt haar schouders op en loopt gewoon door, ze heeft geen zin om nog natter te worden.	Eenmaal in de sportschool kleedt Emma zich om. Dan loopt ze snel naar de loopbanden, waar het altijd druk is. De loopbanden staan boven en halverwege de trap ziet ze dat haar veter los zit. Ze bukt zich om deze te strikken en haast zich dan verder naar boven. Ze ziet dat er nog eentje vrij is, maar een andere vrouw loopt ook op de band af.	…	Emma	is	dankbaar	wanneer de vrouw die ook wil rennen	haar vriendelijk voor laat gaan
52	Rianne loopt door het bos met de hond. Het is zaterdagmiddag en er schijnt een waterig zonnetje. Terwijl de hond druk bezig is met snuffelen belt Rianne met een vriendin. Ze hebben elkaar al een poosje niet gesproken en praten bij. Rianne doet een verhaal uit de doeken over een dronken avond op zakenreis. Rianne merkt ineens dat ze langs het kerkhof loopt en dat een groep rouwers bij een nieuw graf staat.	Rianne gaat op luide toon verder en vertelt haar vriendin dat een groep zielenpoten haar dom aan staat te kijken.	Later die dag bedenkt Rianne zich dat ze wel zin heeft om wat te doen die avond. Ze is al een hele poos niet meer op stap geweest. Ze stuurt een berichtje op de groepsapp van een groep vrienden van het volleyballen. Ondertussen gaat Rianne alvast iets te eten klaar maken. Tijdens het eten checkt ze haar telefoon en leest de reacties van iedereen.	…	Rianne	wordt	woest	als ze leest dat iedereen	zegt niet te kunnen terwijl ze weet dat ze wel uitgaan
53	Ilse stapt uit de auto en loopt richting het uitvaartcentrum. Ze is hier met een paar andere mensen van het werk voor de uitvaart van een collega. Ze kende de man niet goed, maar sommigen van zijn collega's waren wel close. Ze zoekt samen met haar collega's een plekje achterin. De dienst begint en na het eerste nummer spreekt zijn vrouw. Een collega van Ilse begint zachtjes te huilen.	Ilse zucht diep en pakt haar telefoon om haar email en facebook te checken, wat een dramatisch gedoe!	Een week later heeft Ilse een belangrijke afspraak met haar manager. Het is een vervroegd kwartaalgesprek om haar functioneren te evalueren. Ilse heeft geen redenen om te denken dat haar manager iets te klagen zou hebben. Maar zo'n gesprek is toch altijd spannend en stressvol. Als ze eenmaal zitten en de beleefdheden hebben gehad, steekt haar manager van wal.	…	Ilse	is	opgelucht	wanneer haar manager haar vertelt	dat zij een promotie en opslag krijgt
54	Sander werkt bij een groot softwarebedrijf. De directeur van het bedrijf is kort geleden met pensioen gegaan. Sander zit in de sollicitatiecommissie om een geschikte nieuwe directeur te vinden. De eerste brieven en cv's van kandidaten zijn binnengekomen. Sander print de documenten uit en maakt een overzicht van alle kandidaten. Het valt hem op dat er veel vrouwelijke kandidaten zijn.	Sander gruwt van de gedachte om een vrouw als baas te moeten hebben, en hij haalt de vrouwelijke kandidaten allemaal van de lijst.	Wanneer Sander klaar is met werken rijdt hij het half uurtje naar huis. Eenmaal thuis besluit hij een eindje te gaan hardlopen. Hij trekt zijn hardloopkleding aan en loopt zijn huis uit. Na wat warming-up en rekken en strekken gaat hij op pad. Na een half uurtje stevig lopen komt Sander bij een smalle straat waar een groepje jongeren de weg blokkeert.	…	Sander	wordt	boos	als het groepje jongeren	expres de weg blijft blokkeren en lacht
55	Tamara is eigenaar van een koffiezaak. Ze heeft vandaag een aantal sollicitatiegesprekken. Het gaat voornamelijk om tijdelijke krachten die in de vakantieperiode bijspringen. Toch moet ze ook die zorgvuldig selecteren. De eerste drie kandidaten zijn niet super geschikt op het eerste gezicht. Tamara is verbaasd als de volgende een enigszins ruig uitziende Turkse jongen blijkt te zijn.	Tamara stopt de jongen voor hij iets kan zeggen en zegt dat haar klanten Nederlanders zijn en ze geen Turk wil.	Aan het einde van de dag is Tamara met het schoonmaakwerk bezig. De balie is schoon en de vloer is geveegd, maar nog niet gedweild. Er zit nog één klant in de hoek en ze zijn officieel nog open. Tamara wacht daarom met dweilen en vult de suikerpotten bij. Tamara loopt met een zwaar dienblad vol suikerpotten als de laatste klant vol tegen haar op botst.	…	Tamara	wordt	furieus	als de klant die haar aanstootte	geen sorry zegt en zelfs geamuseerd omkijkt
56	Thijs stapt snel op de fiets om nog iets te halen in de stad. Hij fietst de straat uit en richting centrum. Hij zet zijn fiets op slot tegen een paal bij het gemeentehuis. Hij hoeft maar één ding te halen en neemt verder niet de tijd om rond te kijken. Hij slalomt door de mensenmassa heen en schiet lekker op. Ineens staat hij vlak voor een gehandicapte jonge vrouw met een rollator.	Thijs groet haar vriendelijk, stapt opzij en knoopt even een praatje met haar aan, ze vindt het duidelijk gezellig.	Thijs vervolgt zijn weg naar de winkel en koopt snel wat hij nodig heeft. Daarna baant hij zich zo snel mogelijk een weg terug door de binnenstad. Halverwege voelt hij ineens een zachte aanraking. Hij voelt in zijn zak, merkt dat zijn portemonnee weg is en ziet ineens iemand snel weglopen. Thijs schreeuwt dat hij bestolen is en de dief zet het op een hollen.	…	Thijs	is	ziedend	als hij ziet hoe de omstanders	domweg opzij gaan en de dief ontsnapt
57	Jeroen is twee weken op vakantie in Italië. Hij heeft een groot deel van de eerste week aan het strand doorgebracht. De tweede week wil hij iets meer cultuur opsnuiven. Vandaag heeft hij de trein genomen naar een oud stadje vlakbij. Er schijnt een heel mooie en oude kerk te staan. Wanneer hij in zijn hemd de kerk wil binnenlopen, wijst iemand hem op een bord met kledingvoorschriften.	Jeroen kijkt verbouwereerd om zich heen, spuugt op de drempel van de kerk en loopt boos weg, wat een onzin.	Jeroen gaat op het terras zitten bij het enige restaurant dat hij heeft gezien. Hij spreekt geen Italiaans en hoopt dat ze Engels spreken. Hij probeert uit te puzzelen wat er op de kaart staat, hij is vegetariër. Het is een warme dag en Jeroen is blij met de schaduw terwijl hij op de ober wacht. Als de ober langskomt, vraagt Jeroen of hij Engels spreekt.	…	Jeroen	is	witheet	als de ober vervolgens	zonder wat te zeggen omdraait en hem negeert
58	Patrick heeft sinds een jaar een eigen bedrijf. Het bedrijf is zo gegroeid dat Patrick een secretaresse nodig heeft. Hij heeft heel veel reacties gehad. Eén sollicitante steekt boven alle anderen uit door haar ervaring en opleiding. Patrick nodigt haar uit en is tijdens het gesprek erg enthousiast. Zijn zakenpartner wijst hem er later op dat ze wel wat ouder is en andere kandidaten er leuker uitzagen.	Patrick maakt dat echter niks uit en neemt haar gelijk aan en berispt de man voor zijn seksistische opmerking.	Later die dag werkt Patrick aan een verslag voor een klant van zijn bedrijf. Het is een eindrapportage van alle werkzaamheden. Het is een groot document geworden dat Patrick veel kostbare tijd heeft gekost. Wanneer Patrick de laatste punt neerzet, valt ineens de stroom uit. Patrick bedenkt zich dat hij tussendoor niet heeft gesaved en start de computer weer op.	…	Patrick	is	opgelucht	wanneer na het opnieuw opstarten blijkt	dat er een automatische back-up beschikbaar is
59	Anna staat aan de kassa bij de supermarkt. Terwijl de kassière de laatste boodschappen scant, pakt ze zoveel mogelijk in. Nadat ze het laatste artikel heeft gescand noemt ze het bedrag. Anna betaalt met 50 euro en de kassière geeft haar wisselgeld terug. Anna ziet meteen dat ze haar veel te veel wisselgeld geeft. Ze weet dat kassières flink problemen kunnen krijgen bij een kasverschil.	Anna zegt lekker niks en neemt het geld aan, dan moet ze maar beter opletten, zij is niet verantwoordelijk.	Na de supermarkt gaat ze ook nog even naar de boekwinkel. Ze moet echter opschieten want haar parkeerkaartje verloopt zo. Bij de boekwinkel kan ze niet gelijk vinden wat ze zoekt. Ze vraagt het de eigenaar en die helpt haar het boek te vinden. Anna rekent af en loopt, zo snel als ze kan met alle tassen, naar de parkeerplaats, ze is nu echt te laat.	…	Anna	wordt	vrolijk	als de parkeerwachter haar ziet	en besluit de boete te verscheuren
60	Nienke loopt door het park op een zondagochtend. Ze is op weg naar de winkel om brood te halen. In de verte ziet ze een oud mannetje met een rollator lopen. Nienke loopt rustig door, maar heeft de man uiteraard al gauw bijna ingehaald. De man stopt even en graaft een zakdoek uit zijn broekzak. Nienke ziet hoe tegelijk met de zakdoek ook een pasje op de grond valt, maar de man merkt niks.	Nienke zegt niks, het blijkt een creditcard te zijn en ze gaat snel naar huis en shopt een eind weg online.	Later die week loopt Nienke met een laptop onder de arm. Ze is onderweg naar de winkel waar ze hem de dag daarvoor heeft gekocht. Toen ze hem gisteravond probeerde te installeren bleek hij het niet te doen. Ze is helaas het bonnetje kwijt, maar hoopt dat ze haar nog herkennen. De man in de winkel herkent haar inderdaad en vraagt om het bonnetje.	…	Nienke	is	gepikeerd	als de man vervolgens zegt dat	ze zonder bonnetje naar haar geld kan fluiten
61	Jeffrey is alweer een tijdje vrijgezel. Sinds kort is er bij Jeffrey op het werk een meisje dat verliefd is op Jeffrey. Jeffrey weet dit, maar heeft absoluut geen gevoelens voor haar. Toch vindt Jeffrey de aandacht wel leuk en hij flirt er lustig op los. Na een avond uit met collega's zijn Jeffrey en het meisje alleen overgebleven. Het meisje vraagt Jeffrey ineens blozend om met haar mee naar huis te gaan.	Jeffrey wil geen misbruik van haar gevoelens maken en zegt vriendelijk dat ze beter alleen naar huis kan gaan.	Later die week gaat Jeffrey naar de boekhandel. Het nieuwste deel van Jeffreys favoriete boekenserie is namelijk uit. Bovendien heeft de boekhandel vandaag een actie: de eerste 100 klanten krijgen het boek gratis. De winkel staat op het punt open te gaan en er staat al een grote menigte voor de deur. Jeffrey sluit achter aan en schat zijn kansen in.	…	Jeffrey	is	dolblij	wanneer hij na een uur wachten	de 100ste klant is en het boek gratis krijgt
62	Tessa loopt na de film naar de tramhalte twee straten verderop. Onderweg komt ze langs de Febo en ze beseft ineens dat ze honger heeft. Ze trekt een kroket uit de muur en loopt verder. Aan het einde van de straat slaat ze rechtsaf naar het plein. Ze steekt het plein schuin over naar de halte. Op het plein passeert ze twee mannen die hand in hand lopen en heel gelukkig lijken.	Tessa knikt het stel heel vriendelijk toe, ze vindt het altijd mooi om mensen samen gelukkig te zien.	Een korte tramrit later is Tessa bijna thuis. Ze neemt de lift naar haar appartement op de vijfde etage. In de lift ziet ze dat één van haar veters los is en ze bukt om die te strikken. Als ze de lift uitkomt en de galerij op loopt ziet ze haar voordeur open staan. Ze herinnert zich in een flits dat ze de deur zelf niet goed dicht heeft gedaan.	…	Tessa	is	laaiend	als ze naar binnen rent en ziet dat	alles overhoop ligt en dure dingen zijn gejat
63	Leonie fietst 's avonds na een etentje bij vrienden naar huis. Op een gegeven moment nadert Leonie een kruispunt. Van links komt een meisje ook op het kruispunt af, ze is zichtbaar gehaast. Leonie heeft voorrang en steekt dus het kruispunt over. Het meisje ziet JLeonie niet aankomen en rijdt ook door. Het meisje schrikt wanneer ze pas op het laatste moment Leonie ziet en valt met haar fiets op straat.	Leonie stapt af, helpt het geschrokken meisje overeind, kalmeert haar en biedt aan haar naar huis te helpen.	Leonie fietst verder en ziet in de verte een kruispunt met verkeerslichten. Ze stopt wanneer ze er aankomt, omdat het licht nog steeds op rood staat. Het licht blijft maar op rood staan, en Leonie wordt ongeduldig. Er komt niks aan en het kruispunt lijkt verlaten. Ze rijdt door het rode licht, maar ziet plots aan de overkant een politieagent staan.	…	Leonie	is	opgelucht	wanneer ze op de agent affietst	en deze knipoogt en zegt dat ze door mag rijden
64	Claudia heeft boodschappen gedaan en loopt naar de auto. Ze laadt haar boodschappen in en stapt in. Claudia start de auto, zet de radio aan en geeft gas. Claudia heeft echter niet door dat de auto in zijn achteruit staat. De auto schiet naar achteren en knalt tegen de auto die erachter geparkeerd staat. Claudia stapt uit en ziet dat de auto achter haar erg beschadigd is, maar haar eigen auto niet.	Claudia schrijft netjes een briefje met adres- en verzekeringsgegevens dat ze onder de ruitenwisser stopt.	Die avond staat Claudia bij bioscoop Tuschinski in Amsterdam. Er is een grote internationale filmpremiére met veel beroemde acteurs. Claudia hoopt bij de rode loper een glimp van de sterren op te vangen. Tot de aanwezige sterren behoort ook Johnny Depp, een acteur die Claudia erg bewondert. Op een gegeven moment ziet Claudia hem op de rode loper verschijnen.	…	Claudia	is	uitzinnig	als Johnny Depp vervolgens	rustig de tijd neemt om met haar te kletsen

Experimental Stimulus List 4

Number	Introduction	Character Manipulation	Continuation	Transition	Name	Verb	Affective State Adjective	Neutral Segment	Affect Reason
1	Claudia heeft boodschappen gedaan en loopt naar de auto. Ze laadt haar boodschappen in en stapt in. Claudia start de auto, zet de radio aan en geeft gas. Claudia heeft echter niet door dat de auto in zijn achteruit staat. De auto schiet naar achteren en knalt tegen de auto die erachter geparkeerd staat. Claudia stapt uit en ziet dat de auto achter haar erg beschadigd is, maar haar eigen auto niet.	Claudia schrijft netjes een briefje met adres- en verzekeringsgegevens dat ze onder de ruitenwisser stopt.	Die avond staat Claudia bij bioscoop Tuschinski in Amsterdam. Er is een grote internationale filmpremière met veel beroemde acteurs. Claudia hoopt bij de rode loper een glimp van de sterren op te vangen. Tot de aanwezige sterren behoort ook Johnny Depp, een acteur die Claudia erg bewondert. Op een gegeven moment ziet Claudia hem op de rode loper verschijnen.	…	Claudia	is	teleurgesteld	als Johnny Depp vervolgens	haar nors negeert en ze zelfs geen handtekening krijgt
2	Jasper fietst 's avonds na een etentje bij vrienden naar huis. Op een gegeven moment nadert Jasper een kruispunt. Van links komt een meisje ook op het kruispunt af, ze is zichtbaar gehaast. Jasper heeft voorrang en steekt dus het kruispunt over. Het meisje ziet Jasper niet aankomen en rijdt ook door. Ze schrikt wanneer ze pas op het laatste moment Jasper ziet en valt met haar fiets op straat.	Jasper stapt af, helpt het geschrokken meisje overeind, kalmeert haar en biedt aan haar naar huis te helpen.	Jasper fietst verder en ziet in de verte een kruispunt met verkeerslichten. Hij stopt wanneer hij er aankomt, omdat het licht nog steeds op rood staat. Het licht blijft maar op rood staan, en Jasper wordt ongeduldig. Er komt niks aan en het kruispunt lijkt verlaten. Hij rijdt door het rode licht, maar ziet plots aan de overkant een politieagent staan.	…	Jasper	is	chagrijnig	wanneer hij op de agent affietst	en deze hem toch een fikse boete geeft
3	Tessa loopt na de film naar de tramhalte twee straten verderop. Onderweg komt ze langs de Febo en ze beseft ineens dat ze honger heeft. Ze trekt een kroket uit de muur en loopt verder. Aan het einde van de straat slaat ze rechtsaf naar het plein. Ze steekt het plein schuin over naar de halte. Op het plein passeert ze twee mannen die hand in hand lopen en heel gelukkig lijken.	Tessa knikt het stel heel vriendelijk toe, ze vindt het altijd mooi om mensen samen gelukkig te zien.	Een korte tramrit later is Tessa bijna thuis. Ze neemt de lift naar haar appartement op de vijfde etage. In de lift ziet ze dat één van haar veters los is en ze bukt om die te strikken. Als ze de lift uitkomt en de galerij op loopt ziet ze haar voordeur open staan. Ze herinnert zich in een flits dat ze de deur zelf niet goed dicht heeft gedaan.	…	Tessa	is	uitgelaten	als ze naar binnen rent en ziet dat	er niks weg is, wat een geluk!
4	Marloes is alweer een tijdje vrijgezel. Sinds kort is er bij Marloes op het werk een jongen die verliefd is op Marloes. Marloes weet dit, maar heeft absoluut geen gevoelens voor hem. Toch vindt Marloes de aandacht wel leuk en ze flirt er lustig op los. Na een avond uit met collega's zijn Marloes en de jongen alleen overgebleven. De jongen vraagt Marloes ineens blozend om met hem mee naar huis te gaan.	Marloes wil geen misbruik van zijn gevoelens maken en zegt vriendelijk dat hij beter alleen naar huis kan gaan.	Later die week gaat Marloes naar de boekhandel. Het nieuwste deel van Marloes haar favoriete boekenserie is namelijk uit. Bovendien heeft de boekhandel vandaag een actie: de eerste 100 klanten krijgen het boek gratis. De winkel staat op het punt open te gaan en er staat al een grote menigte voor de deur. Marloes sluit achter aan en schat haar kansen in.	…	Marloes	is	verbolgen	wanneer ze na een uur wachten	de 101ste klant is en de volle prijs moet betalen
5	Nienke loopt door het park op een zondagochtend. Ze is op weg naar de winkel om brood te halen. In de verte ziet ze een oud mannetje met een rollator lopen. Nienke loopt rustig door, maar heeft de man uiteraard al gauw bijna ingehaald. De man stopt even en graaft een zakdoek uit zijn broekzak. Nienke ziet hoe tegelijk met de zakdoek ook een pasje op de grond valt, maar de man merkt niks.	Nienke zegt niks, het blijkt een creditcard te zijn en ze gaat snel naar huis en shopt een eind weg online.	Later die week loopt Nienke met een laptop onder de arm. Ze is onderweg naar de winkel waar ze hem de dag daarvoor heeft gekocht. Toen ze hem gisteravond probeerde te installeren bleek hij het niet te doen. Ze is helaas het bonnetje kwijt, maar hoopt dat ze haar nog herkennen. De man in de winkel herkent haar inderdaad en vraagt om het bonnetje.	…	Nienke	is	gelukkig	als de man vervolgens zegt dat	ze ook zonder bonnetje haar geld terugkrijgt
6	Anna staat aan de kassa bij de supermarkt. Terwijl de kassière de laatste boodschappen scant, pakt ze zoveel mogelijk in. Nadat ze het laatste artikel heeft gescand noemt ze het bedrag. Anna betaalt met 50 euro en de kassière geeft haar wisselgeld terug. Anna ziet meteen dat ze haar veel te veel wisselgeld geeft. Ze weet dat kassières flink problemen kunnen krijgen bij een kasverschil.	Anna zegt lekker niks en neemt het geld aan, dan moet ze maar beter opletten, zij is niet verantwoordelijk.	Na de supermarkt gaat ze ook nog even naar de boekwinkel. Ze moet echter opschieten want haar parkeerkaartje verloopt zo. Bij de boekwinkel kan ze niet gelijk vinden wat ze zoekt. Ze vraagt het de eigenaar en die helpt haar het boek te vinden. Anna rekent af en loopt, zo snel als ze kan met alle tassen, naar de parkeerplaats, ze is nu echt te laat.	…	Anna	wordt	kwaad	als de parkeerwachter haar ziet	en toch alsnog de boete uitschrijft
7	Patrick heeft sinds een jaar een eigen bedrijf. Het bedrijf is zo gegroeid dat Patrick een secretaresse nodig heeft. Hij heeft heel veel reacties gehad. Eén sollicitante steekt boven alle anderen uit door haar ervaring en opleiding. Patrick nodigt haar uit en is tijdens het gesprek erg enthousiast. Zijn zakenpartner wijst hem er later op dat ze wel wat ouder is en andere kandidaten er leuker uitzagen.	Patrick maakt dat echter niks uit en neemt haar gelijk aan en berispt de man voor zijn seksistische opmerking.	Later die dag werkt Patrick aan een verslag voor een klant van zijn bedrijf. Het is een eindrapportage van alle werkzaamheden. Het is een groot document geworden dat Patrick veel kostbare tijd heeft gekost. Wanneer Patrick de laatste punt neerzet, valt ineens de stroom uit. Patrick bedenkt zich dat hij tussendoor niet heeft gesaved en start de computer weer op.	…	Patrick	is	ziedend	wanneer het opnieuw opstarten blijkt	dat het document verloren is en het opnieuw moet
8	Jeroen is twee weken op vakantie in Italië. Hij heeft een groot deel van de eerste week aan het strand doorgebracht. De tweede week wil hij iets meer cultuur opsnuiven. Vandaag heeft hij de trein genomen naar een oud stadje vlakbij. Er schijnt een heel mooie en oude kerk te staan. Wanneer hij in zijn hemd de kerk wil binnenlopen, wijst iemand hem op een bord met kledingvoorschriften.	Jeroen kijkt verbouwereerd om zich heen, spuugt op de drempel van de kerk en loopt boos weg, wat een onzin.	Jeroen gaat op het terras zitten bij het enige restaurant dat hij heeft gezien. Hij spreekt geen Italiaans en hoopt dat ze Engels spreken. Hij probeert uit te puzzelen wat er op de kaart staat, hij is vegetariër. Het is een warme dag en Jeroen is blij met de schaduw terwijl hij op de ober wacht. Als de ober langskomt, vraagt Jeroen of hij Engels spreekt.	…	Jeroen	is	vergenoegd	als de ober vervolgens	geduldig zijn best doet om Engels te spreken
9	Thijs stapt snel op de fiets om nog iets te halen in de stad. Hij fietst de straat uit en richting centrum. Hij zet zijn fiets op slot tegen een paal bij het gemeentehuis. Hij hoeft maar één ding te halen en neemt verder niet de tijd om rond te kijken. Hij slalomt door de mensenmassa heen en schiet lekker op. Ineens staat hij vlak voor een gehandicapte jonge vrouw met een rollator.	Thijs groet haar vriendelijk, stapt opzij en knoopt even een praatje met haar aan, ze vindt het duidelijk gezellig.	Thijs vervolgt zijn weg naar de winkel en koopt snel wat hij nodig heeft. Daarna baant hij zich zo snel mogelijk een weg terug door de binnenstad. Halverwege voelt hij ineens een zachte aanraking. Hij voelt in zijn zak, merkt dat zijn portemonnee weg is en ziet ineens iemand snel weglopen. Thijs schreeuwt dat hij bestolen is en de dief zet het op een hollen.	…	Thijs	is	blij	als hij ziet hoe de omstanders	de dief grijpen voordat deze weg kan komen
10	Tamara is eigenaar van een koffiezaak. Ze heeft vandaag een aantal sollicitatiegesprekken. Het gaat voornamelijk om tijdelijke krachten die in de vakantieperiode bijspringen. Toch moet ze ook die zorgvuldig selecteren. De eerste drie kandidaten zijn niet super geschikt op het eerste gezicht. Tamara is verbaasd als de volgende een enigszins ruig uitziende Turkse jongen blijkt te zijn.	Tamara stopt de jongen voor hij iets kan zeggen en zegt dat haar klanten Nederlanders zijn en ze geen Turk wil.	Aan het einde van de dag is Tamara met het schoonmaakwerk bezig. De balie is schoon en de vloer is geveegd, maar nog niet gedweild. Er zit nog één klant in de hoek en ze zijn officieel nog open. Tamara wacht daarom met dweilen en vult de suikerpotten bij. Tamara loopt met een zwaar dienblad vol suikerpotten als de laatste klant vol tegen haar op botst.	…	Tamara	wordt	blij	als de klant die haar aanstootte	aardig blijkt te zijn en helpt op te ruimen
11	Sander werkt bij een groot softwarebedrijf. De directeur van het bedrijf is kort geleden met pensioen gegaan. Sander zit in de sollicitatiecommissie om een geschikte nieuwe directeur te vinden. De eerste brieven en cv's van kandidaten zijn binnengekomen. Sander print de documenten uit en maakt een overzicht van alle kandidaten. Het valt hem op dat er veel vrouwelijke kandidaten zijn.	Sander gruwt van de gedachte om een vrouw als baas te moeten hebben, en hij haalt de vrouwelijke kandidaten allemaal van de lijst.	Wanneer Sander klaar is met werken rijdt hij het half uurtje naar huis. Eenmaal thuis besluit hij een eindje te gaan hardlopen. Hij trekt zijn hardloopkleding aan en loopt zijn huis uit. Na wat warming-up en rekken en strekken gaat hij op pad. Na een half uurtje stevig lopen komt Sander bij een smalle straat waar een groepje jongeren de weg blokkeert.	…	Sander	wordt	blij	als het groepje jongeren	gauw aan de kant gaat zodat hij door kan lopen
12	Bart stapt uit de auto en loopt richting het uitvaartcentrum. Hij is hier met een paar andere mensen van het werk voor de uitvaart van een collega. Hij kende de man niet goed, maar sommigen van zijn collega's waren wel close. Hij zoekt samen met zijn collega's een plekje achterin. De dienst begint en na het eerste nummer spreekt zijn vrouw. Een collega van Bart begint zachtjes te huilen.	Bart zucht diep en pakt zijn telefoon om zijn email en facebook te checken, wat een dramatisch gedoe!	Een week later heeft Bart een belangrijke afspraak met zijn manager. Het is een vervroegd kwartaalgesprek om zijn functioneren te evalueren. Bart heeft geen redenen om te denken dat zijn manager iets te klagen zou hebben. Maar zo'n gesprek is toch altijd spannend en stressvol. Als ze eenmaal zitten en de beleefdheden hebben gehad, steekt zijn manager van wal.	…	Bart	is	furieus	wanneer zijn manager hem vertelt	dat hij per direct ontslag krijgt
13	Rianne loopt door het bos met de hond. Het is zaterdagmiddag en er schijnt een waterig zonnetje. Terwijl de hond druk bezig is met snuffelen belt Rianne met een vriendin. Ze hebben elkaar al een poosje niet gesproken en praten bij. Rianne doet een verhaal uit de doeken over een dronken avond op zakenreis. Rianne merkt ineens dat ze langs het kerkhof loopt en dat een groep rouwers bij een nieuw graf staat.	Rianne gaat op luide toon verder en vertelt haar vriendin dat een groep zielenpoten haar dom aan staat te kijken.	Later die dag bedenkt Rianne zich dat ze wel zin heeft om wat te doen die avond. Ze is al een hele poos niet meer op stap geweest. Ze stuurt een berichtje op de groepsapp van een groep vrienden van het volleyballen. Ondertussen gaat Rianne alvast iets te eten klaar maken. Tijdens het eten checkt ze haar telefoon en leest de reacties van iedereen.	…	Rianne	wordt	vrolijk	als ze leest dat iedereen	enthousiast is en ze een leuke avond heeft
14	Mike is op weg naar de sportschool op een doordeweekse avond. Het begint net wat te schemeren en het regent gestaag. Hij steekt over bij de stoplichten en gaat de hoek om. Aan het eind van de straat ziet hij mensen in en uit lopen bij de sportschool. Aan de overkant van de straat fietst een oudere vrouw de andere kant op. Mike ziet hoe ze plots begint te slingeren en met fiets en al omvalt.	Mike kijkt even en haalt zijn schouders op en loopt gewoon door, hij heeft geen zin om nog natter te worden.	Eenmaal in de sportschool kleedt Mike zich om. Dan loopt hij snel naar de loopbanden, waar het altijd druk is. De loopbanden staan boven en halverwege de trap ziet hij dat zijn veter los zit. Hij bukt zich om deze te strikken en haast zich dan verder naar boven. Hij ziet dat er nog eentje vrij is, maar een andere man loopt ook op de band af.	…	Mike	is	gepikeerd	wanneer de man die ook wil rennen	net voor hem op de band springt
15	Niels is 's ochtends vroeg als eerste op kantoor. Het is verder muisstil op de afdeling. Na een half uurtje heeft Niels al flink wat werk verzet, dit is zijn favoriete deel van de dag. Hij legt de laatste hand aan een document en streept hem van zijn lijstje. Vervolgens gaat hij koffie halen en komt de eerste collega tegen. Niels kent haar vaag en weet toevallig dat ze sinds kort op dieet is.	Niels zegt haar goedemorgen en complimenteert haar met het resultaat van het dieet en hoe goed ze eruitziet.	Niels werkt rustig door tot de lunch en daarna heeft hij een vergadering. Zijn team presenteert vandaag de resultatenvan een project. Het project is een enorm succes geworden voor het bedrijf. Niels presenteert vandaag zelf niet, maar hij weet dat hij een integraal onderdeel van dit succes is. De belangrijkste ideeën voor het nieuwe programma kwamen van hem.	…	Niels	voelt	blijdschap	wanneer zijn manager in de meeting	hem prijst voor zijn originaliteit en inzet
16	Tom loopt naar huis van de supermarkt aan de overkant van de straat. Hij heeft één tas met boodschappen, maar die weegt niet zoveel. Met zijn vrije hand opent hij de zware portiekdeur van de flat. Achter hem loopt een vrouw met twee tassen, ze heeft moeite met het gewicht. Tom herkent haar en weet dat ze ook de flat in moet. Hij stopt even in de deuropening en denkt na wat te doen.	Hij besluit lekker toch gauw door te lopen en laat de deur mooi in het gezicht van de vrouw dicht vallen.	Later die dag wil Tom een afspraak maken bij de dokter. Hij zoekt het nummer op en pakt de telefoon. Hij wil al een hele tijd een afspraak maken vanwege een zere knie. Tom is echter zo druk dat hij vaak geen zin heeft om te bellen, het wachten duurt altijd zo lang. Hij moet eigenlijk al weer de deur uit, maar wil toch even snel bellen.	…	Tom	is	pissig	wanneer de telefoon over gaat	en hij de 15de wachtende blijkt te zijn
17	Elise zit in de bus op weg naar de film. Ze heeft haar oordopjes in en zoekt de filmtijden nog eens op. De bus is halverwege en ze sms't haar vrienden dat ze eraan komt. Elise verandert de muziek naar iets stevigers en zet het harder. Er zitten weinig mensen in de bus behalve twee oudere dames voor haar. Eén van hen draait zich om om te zeggen dat haar muziek wel heel hard staat.	Elise zet haar muziek lekker nog harder en maakt een obsceen gebaar naar haar, waar bemoeit die ouwe tang zich mee?	Twee haltes verder stapt Elise uit en loopt richting bioscoop. Bij de ingang ontmoet ze haar vrienden en ze gaan gauw naar de zaal. Na de film gaan ze nog even gezellig wat drinken. Elise bestelt een biertje, maar bedenkt zich later en loopt naar de bar om iets anders te bestellen. Ze zegt tegen de serveerster dat ze toch liever een witte wijn heeft.	…	Elise	wordt	vrolijk	als de serveerster reageert	op een vriendelijke manier en ze kletsen wat
18	Wouter werkt op de marketingafdeling van een groot bedrijf. Op dezelfde afdeling werkt zijn beste vriend. Ze werken al jaren heel veel samen en ze zijn een hecht team. Op een ochtend wordt Wouters vriend naar het kantoor van de baas geroepen. Wouter weet niet waar het over gaat, maar hij is wel nieuwsgierig. Later die dag hoort Wouter dat zijn vriend een promotie heeft gekregen en hij niet.	Wouter is teleurgesteld, maar blij te hebben bijgedragen aan het succes van zijn vriend en feliciteert hem.	Later die week heeft Wouter een zeldzame vrije dag. Hij besluit de meubelzaken bij langs te gaan voor nieuwe meubels. Bij IKEA ziet hij een leuke garderobekast, die perfect past in zijn slaapkamer. Wouter aarzelt geen moment en koopt de kast meteen en gaat weer richting huis. Wouter begint thuis meteen enthousiast het bouwpakket in elkaar te zetten.	…	Wouter	is	woedend	als bij het monteren blijkt	dat de planken scheef zijn en er dingen ontbreken
19	Eva loopt snel over het plein richting het station. Ze gaat vandaag naar Haarlem voor een sollicitatiegesprek. Ze heeft zich goed voorbereid en is ruim op tijd van huis gegaan. Vlakbij de ingang naar de stationshal staat een zwerver te bedelen. Wanneer Eva langsloopt, houdt hij een papieren bekertje omhoog met wat kleingeld erin. De man kijkt hoopvol hoe Eva in haar broekzak graaft.	Eva vist wat snoeppapiertjes op en gooit deze in het bekertje van de zwerver, laat hem werk gaan zoeken.	Ze loopt snel door en kijkt op het bord welk spoor ze moet hebben. Ze ziet dat er over 2 minuten een trein gaat en zet het op een rennen. Als ze deze trein kan halen, dan komt ze sowieso op tijd. Het is druk in de tunnel naar de sporen en Eva moet steeds uitwijken. Eindelijk bereikt ze de trap en sprint met drie treden per keer naar boven.	…	Eva	is	woedend	als de conducteur haar ziet rennen	de deuren expres voor haar neus sluit
20	Hendrik zit 's ochtends al vroeg op het kantoor. Hij is druk bezig met het tentamen schrijven dat zijn studenten volgende week moeten maken. Terwijl hij daar mee bezig is wordt er op de deur geklopt. Het is één van de studentes uit zijn werkgroep, een knappe verschijning. Het is al gauw duidelijk dat ze probeert Hendrik te verleiden om haar het tentamen in te laten zien. Ze zinspeelt op hoe dankbaar ze zou zijn.	Hendrik vertelt haar in niet mis te verstane bewoordingen dat hij daar niet voor in is en dat ze weg moet gaan.	Later die dag moet Hendrik college geven in een gebouw buiten de binnenstad. Hij pakt de fiets daarheen en trapt stevig door. Op een gegeven moment fietst hij op een drukke kruising af en stopt hij voor het licht. Even later springt het op groen en Hendrik steekt over. Halverwege de kruising vliegt de ketting er ineens af en hij staat hij midden op de kruising stil.	…	Hendrik	is	getergd	als de auto's die nu groen hebben	ongeduldig beginnen te toeteren en optrekken
21	Larissa is lerares op een basisschool. Ze geeft les aan de bovenbouw, groep vijf en zes. Op een woensdagochtend heeft ze pleinwacht tijdens de pauze. Ze loopt grote rondes over het plein en houdt alles goed in de gaten. Af en toe spreekt ze een leerling aan, maar verder is het rustig. Dan ziet ze een stel leerlingen uit groep 8 aan de rand van het plein rommel staan gooien naar een vrouw die langsloopt.	Larissa roept de jongens meteen tot de orde, zorgt dat ze hun excuses aanbieden en laat ze 's middags nablijven.	Later die week gaat Larissa naar de bank voor een afspraak. Ze wil haar hypotheek oversluiten en snapt niet veel van financiën, dus wil ze persoonlijk advies. Ze loopt de bank binnen en gaat naar de balie om zich te melden. De receptioniste zegt haar plaats te nemen, ze komen haar zo halen. Larissa gaat zitten en leest een tijdschrift terwijl ze rustig wacht.	…	Larissa	is	laaiend	als ze twee uur later naar buiten loopt	zonder resultaat omdat ze haar afspraak waren vergeten
22	Julia zit op haar plek achter de receptie, de telefoon staat roodgloeiend. Ze handelt het ene na het andere telefoontje af. Voordat Julia het weet is de ochtend alweer voorbij. Na de lunch is het iets minder druk. Dan gaat de telefoon weer, het is iemand voor de man van accounting. De accountant heeft de laatste tijd steken laten vallen en ook nu belt er iemand met een probleem.	Julia loopt snel even naar de man toe om hem de kans te geven de fout te herstellen voordat iemand anders het merkt.	Later die week is Julia aan het winkelen. Ze zoekt een specifiek computerspelletje voor de verjaardag van haar broertje. Ze heeft hem beloofd dat hij deze morgen voor zijn verjaardag krijgt. Julia heeft echter weinig tijd gehad om te winkelen de laatste tijd. Eindelijk vind ze één kopie van het spelletje, maar een andere klant ziet hem tegelijkertijd.	…	Julia	is	vrolijk	als de andere klant het spelletje	aan haar geeft zodat ze haar cadeau heeft
23	Inge zit 's middags in de trein op weg naar Groningen. Het is ontzettend rustig in de trein sinds Zwolle. Er zitten naast Inge maar vier mensen in de hele coupé. Er zit een man in de vierzits aan de overzijde van het gangpad een boek te lezen. Wanneer de trein weer stopt staat de man op om uit te stappen. Ineens ziet Inge dat de man een tas onder de bank heeft laten staan.	Inge houdt zich lekker stil en wacht tot de trein weer rijdt en spit dan de tas door voor waardevolle spullen.	Eenmaal in Groningen aangekomen pakt Inge de bus richting Oranjewijk. Ze logeert bij een vriendin en ze gaan naar een concert in de Oosterpoort vandaag. De bus doet er iets langer over dan normaal, maar Inge heeft geen haast. Ze stapt uit en loopt de laatste vijf minuten naar haar huis. Vlak voor ze aanbelt, gaat haar telefoon en ze neemt op.	…	Inge	voelt	woede	wanneer ze te horen krijgt	dat er bij haar is ingebroken en alles weg is
24	Jesse komt 's middags rond half vier aanrijden bij de school. Hij parkeert in de rij wachtende ouders. Er zijn nog geen kinderen op het plein en hij wacht rustig. Hij is zo verzonken in gedachten dat hij niet merkt dat de kinderen naar buiten komen. Hij is dan ook verrast wanneer zijn dochtertje plots achterin klimt. Ze heeft een tekening gemaakt die ze vol trots laat zien en aan hem wil geven.	Jesse prijst haar tekening uitbundig en zegt dat ze met zo'n mooie tekening wel een ijsje verdiend heeft.	Later die avond als zijn dochtertje al op bed ligt kijkt Jesse voetbal. Hij heeft geld gezet op de uitkomst van deze wedstrijd. Het was een flink bedrag en hij wil graag winnen, want hij kon het geld eigenlijk niet missen. Vlak voor tijd staat het 1-1 en hij heeft geld gezet op 2-1 winst. Hij kijkt gespannen toe hoe zijn team een laatste aanval inzet.	…	Jesse	is	uitgelaten	wanneer vlak voor het einde	zijn team scoort en hij dus 300 euro wint

25	Anouk zit alvast in de vergaderruimte waar ze straks overleg heeft. Ze neemt de documenten die ze heeft voorbereid nog eens door. In de excel sheets van de begroting spot ze ineens een grove fout. Deze fout betekent dat het project veel meer gaat kosten dan ze had gezegd. Anouk heeft weinig zin om dit op te moeten biechten. Voordat ze echt na heeft kunnen denken komen haar bazen binnen.	Anouk begint de vergadering met de fout eerlijk toe te geven en gelukkig wordt haar eerlijkheid gewaardeerd.	De vergadering verloopt verder prima en Anouk gaat door met haar dag. Tegen een uur of acht is ze thuis en haalt de post op. Eenmaal binnen ziet ze een brief van het elektriciteitsbedrijf. Het is de jaarafrekening en dat was vorig jaar een dure grap. Anouk heeft toen het voorschot bij laten stellen en hopelijk hoeft ze nu niet bij te betalen.	…	Anouk	is	opgetogen	als uit de brief blijkt dat ze	dit jaar zelf een fiks bedrag terugkrijgt
26	Kevin loopt 's avonds naar het huis van zijn ouders. Hij is bij een vriend geweest die hij niet had gezien sinds hij is verhuisd. Het is schemerig en het is een eind lopen, maar dat vindt Kevin niet erg. Het is fijn om weer door bekende straten te slenteren. Verderop ziet Kevin een gedaante op vier poten uit de bosjes komen. Het is een gewonde hond die zacht jankt en naar hem toe loopt.	Kevin bedenkt zich niet en geeft het beest een flinke schop en de hond verdwijnt jankend weer de bosjes in.	Eenmaal thuis na het incident met de hond gaat Kevin direct naar bed. De volgende dag staat hij vroeg op om nog wat mensen op te zoeken. Hij leent de auto van zijn ouders omdat sommigen buiten de stad wonen. Halverwege, midden tussen de weilanden, houdt de motor er mee op. Na 20 minuten wachten langs de weg komt er eindelijk een andere auto aan.	…	Kevin	is	pissig	wanneer de andere automobilist	wel langzamer gaat rijden, maar niet stopt
27	Bram is op stap met een groepje vrienden. Ze kennen elkaar nog van hun studietijd en spreken af en toe af. Bram heeft al een aantal biertjes op en de rest heeft hem ook aardig zitten. Op een gegeven moment besluiten ze naar de volgende kroeg te gaan. Voor de ingang van de kroeg staat een groepje kerels te roken. Één van Bram zijn vrienden loopt op ze af en begint ruzie te zoeken.	Bram bedenkt zich niet en haalt uit naar één van de andere jongens in het groepje, die gelijk plat gaat.	Daarna is de avond snel afgelopen en gaat Bram naar zijn fiets. Zijn fiets staat helemaal aan de andere kant van het centrum. Het is ondertussen drie uur geweest en hij wil niets liever dan slapen. Het schiet Bram ineens te binnen dat hij zijn fiets helemaal niet op slot heeft gezet. Hij graaft in zijn zakken en kan de sleutel nergens vinden.	…	Bram	is	razend	als hij aankomt waar hij zijn fiets had staan	en hij echt gejat is en hij naar huis kan lopen
28	Myrthe krijgt haar schoonouders vanavond op bezoek en ze blijven eten. Ze wil indruk op hen maken met haar kookkunsten. Ze heeft alleen nog niks in huis en rijdt daarom naar de supermarkt. Myrthe parkeert haar auto op de parkeerplaats en loopt naar de ingang van de supermarkt. Bij de ingang staat een man die overduidelijk dakloos is. De man loopt op Myrthe af en vraagt om wat kleingeld.	Myrthe vist wat kleingeld uit haar broekzak en geeft het aan de man, die er overduidelijk blij mee is.	Wanneer Myrthe de boodschappen heeft gekocht, rijdt ze weer naar huis. Thuis aangekomen loopt ze met haar boodschappen naar de voordeur. Myrthe kan haar voordeursleutel niet vinden. Op dat moment bedenkt ze zich dat ze zonder sleutel de deur achter zich dicht heeft gedaan. Snel loopt ze naar het huis van haar buren, die een reservesleutel hebben.	…	Myrthe	is	getergd	als ze bij de buren aanbelt en	die op vakantie zijn en ze een slotenmaker moet bellen
29	Mark rent snel terug richting het eigen doel na de gefaalde aanval. De tegenstander heeft net de bal terugveroverd. Zijn team heeft moeite met terugschakelen naar verdedigen. Over de flank krijgt de spits van de tegenstander een geweldige lange pass. Mark sprint om hem af te snijden op weg naar het doel. Mark zet een tackle in en beseft zich meteen dat hij veel te hard in heeft gezet.	Mark strekt zijn been expres nog verder en met genoegen voelt hij de enkel van de tegenstander knakken.	De bal komt bij de keeper terecht en die speelt Mark aan. Mark neemt de bal aan en begint een soloactie langs de lijn. Hij passeert twee man en een derde komt veel te laat aanrennen. Mark trekt naar binnen richting de zestien meter van de tegenstander. Zijn teamgenoten zijn nog niet mee naar voren en dus legt Mark zelf aan om uit te halen.	…	Mark	is	boos	als hij aanlegt om te schieten	en hij keihard onderuit wordt gehaald
30	Joris loopt 's ochtend over de markt richting station. Hij heeft zijn oordopjes in en luistert naar muziek. De marktkooplui zijn in de weer met het opbouwen van hun kramen. De klinkers zijn wit uitgeslagen van de vorst van vannacht. Een man passeert voor Joris met een stapel kisten vol mandarijnen in zijn armen. De bovenste wankelt wat en even later ligt de straat bezaaid met mandarijnen.	Joris stampt expres twee mandarijnen kapot, schopt een derde tegen een andere kraam en loopt dan snel door.	Later die ochtend komt Joris terug van de lunch. Hij gaat weer achter zijn bureau zitten en kijkt naar zijn lijstje met taken voor vandaag. Hij is lekker opgeschoten vanochtend, maar kan nu eigenlijk niet verder. Hij wacht op bericht van een afdeling in het buitenland. Hij heeft ze gemaild of ze op willen schieten omdat hij niet verder kan zonder de info.	…	Joris	is	blij	als hij een mailtje krijgt met daarin	eindelijk het verlossende woord
31	Tim loopt 's middags door de supermarkt. Op zijn lijstje staat van alles om zelf een verjaardagstaart te maken. Wanneer hij alles heeft gevonden gaat hij naar de kassa. Voor hem in het gangpad loopt een oudere vrouw. Ze is duidelijk ook onderweg naar de kassa, maar ze loopt minder snel dan Tim. Het gangpad is niet zo heel breed en er komt iemand van de andere kant aanlopen.	Tim heeft geen zin om te wachten en haalt haar snel in en sluit lekker vlak voor haar neus aan in de rij.	Later die dag is hij bezig de taart te bakken, hij staat in de oven. Hij viert vandaag zijn verjaardag en heeft iedereen eigengemaakte taart beloofd. Tim staat niet bepaald bekend om zijn culinaire hoogstandjes. Een aantal van zijn vrienden en familie reageerde dan ook nogal sceptisch. Het is nu een kwestie van eer gewordendat deze taart goed lukt.	…	Tim	is	geïrriteerd	wanneer de cake uiteindelijk	aangebrand uit de oven komt
32	Saskia is vrijwilliger bij een grote manege en vandaag hebben ze open dag. Vanuit de hele omgeving komen ouders en vooral kinderen naar de manege. Er worden rijlessen en workshops over paardenverzorging gegeven. Saskia heeft de hele ochtend workshops gegeven.'s Middags laat ze jonge kinderen ritjes maken in de bak. Wanneer Saskia zich omdraait naar het volgende kind ziet ze meteen dat het downsyndroom heeft.	Saskia weigert om haar een ritje te laten maken, het idee om haar aan te moeten raken geeft haar huiveringen.	De volgende ochtend is Saskia vroeg op haar normale werk. Ze werkt bij een groot accountantsbedrijf. Ze moet om 9:00 bij de baas komen en ze weet niet precies waarvoor. Ze heeft er slecht van geslapen vannacht terwijl ze probeerde te verzinnen waar het over zal gaan. Wanneer het eenmaal negen uur is en Saskia bij de baas zit, steekt hij gelijk van wal.	…	Saskia	is	uitzinnig	wanneer de baas in het gesprek	vertelt dat ze promotie krijgt en opslag
33	Dennis stapt de lift in in het ziekenhuis. De vrouw van zijn broer is gisteren bevallen en hij gaat op kraamvisite. Hij heeft een grote knuffel onder zijn arm en een bos bloemen. Hij stapt op de begane grond in en moet op de vijfde verdieping zijn. Op de derde verdieping stopt de lift en stapt een vrouw in. De vrouw is kaal en mager, ze heeft wallen onder haar ogen en een grauwe huid.	Dennis gaat zover mogelijk bij haar vandaan staan en staart naar haar, ze voelt zich zichtbaar ongemakkelijk.	Wanneer hij eenmaal zijn jongste neefje heeft bekeken gaat Dennis weer. Hij is met de bus, die stopt vlak bij hem voor de deur. Hij is binnen een half uurtje thuis en maakt wat te eten. In de keuken ziet hij op het bord zijn to-do lijstje en herinnert zich dat de tuin een wildernis is. Hij werpt een snelle blik naar buiten om te zien hoe erg het is.	…	Dennis	is	dankbaar	als hij de tuin inkijkt en ziet dat	de buurman al het werk voor hem heeft gedaan
34	Ruben zit op de bank met zijn laptop op schoot wanneer de bel gaat. Hij zet de laptop op de bank naast zich en staat op. Het is een pakketje voor iemand anders in het gebouw. Ruben neemt het aan en ziet op de pakbon staan dat het een geluidsinstallatie is. Ruben gaat weer verder met waar hij mee bezig was. Later die dag gaat de deurbel en het is de buurvrouw die haar pakketje komt halen.	Ruben pakt de zware doos voor haar en biedt aan om hem voor haar te tillen, wat ze dankbaar accepteert.	De volgende dag is Ruben onderweg naar huis van de supermarkt. Hij heeft twee volle en zware tassen boodschappen. Bij de stoplichten zet hij de tassen naast zich neer en wacht op groen. Wanneer het groen wordt pakt hij de tassen op en haast zich naar de overkant. Bijna aan de overkant struikelt Ruben over de stoeprand en valt tegen de man voor hem aan.	…	Ruben	wordt	vrolijk	wanneer de man voor hem	hem vriendelijk helpt overeind te blijven
35	Jordy loopt over het strand terug naar zijn handdoek. Onderweg komt hij langs een kraampje dat friet en dergelijke verkoopt. Het is er niet druk, het is überhaupt niet druk vandaag. Ondanks dat het een mooie dag is, is er maar een tiental mensen. Jordy loopt voorbij een meisje met een mitella dat probeert een flesje cola te openen. Het wil niet echt lukken met één hand en ze kijkt gefrustreerd.	Jordy stopt en helpt haar even, het is een kleine moeite en hij zou zelf ook willen dat iemand hem hielp.	Jordy gaat nog een stukje zwemmen in de aanzienlijke golfslag. Daarna gaat hij liggen drogen in de zon en leest zijn boek. Hij gaat helemaal op in het verhaal en de tijd verstrijkt. Op een gegeven moment bedenkt hij zich dat de laatste bus vrij vroeg gaat op zondag. Hij checkt hoe laat het is en bedenkt zich dat hij zal moeten opschieten om de bus te halen.	…	Jordy	is	blij	als de buschauffeur hem ziet	en vriendelijk even wacht zodat hij mee kan
36	Maarten is als enige van zijn team op werkbezoek in het buitenland. De bedoeling is om een kijkje te nemen bij een vergelijkbaar team op het hoofdkantoor. Maarten weet dat dit team op hun projecten uit is. Ze willen maar wat graag dat Maartens afdeling moet sluiten. De teamleider van het rivaliserende team wil een één op één gesprek met Maarten. Hij nodigt Maarten uit om bij hen te komen werken als hij zijn team saboteert.	Maarten overweegt dat geen moment en weigert, hij zou nooit zijn collega's thuis verraden voor eigenbelang.	Later die week vliegt Maarten terug naar huis. Het is een lange vlucht en hij heeft onderweg veel om over na te denken. Als het vliegtuig eindelijk landt wacht hij ongeduldig op zijn bagage. Het is hartstikke vroeg en hij wil zijn vrouw verrassen met een ontbijt op bed. Onderweg naar huis koopt hij allerlei lekkere dingen en gaat dan snel door naar huis.	…	Maarten	voelt	blijdschap	als hij eenmaal thuiskomt	en ze samen een romantische ochtend hebben
37	Remco zit in de auto, op weg naar zijn moeder. Hij is de stad nog niet uit en het spoelt van de regen. Het regent onophoudelijk sinds gistermiddag en er hebben zich flinke plassen gevormd. Op sommige plekken staat de straat volledig blank. Er zijn weinig auto's en al helemaal weinig fietsers en voetgangers op straat. Remco stevent op een gigantische plas af en ziet iemand op de stoep lopen.	Remco gaat langzamer rijden en wijkt ietsje uit om te voorkomen dat hij de wandelaar zeiknat spettert.	Eenmaal buiten de stad schiet het lekker op op de snelweg. Het blijft rustig op de weg en Remco geniet van het landschap en de rit. Hij heeft de radio hard aan staan en zingt luidkeels mee. Wanneer hij naar het dashboard kijkt om de zender bij te stellen ziet hij een lampje. Hij is vergeten te tanken en de benzine was al heel lang echt bijna op.	…	Remco	is	verheugd	als hij na een paar minuten rijden	een bezinestation ziet en hij kan tanken
38	Frank loopt richting de ingang van het strandpaviljoen. Hij heeft met vrienden afgesproken, maar is aan de vroege kant. Frank loopt blootsvoets over het zand door de branding. Ter hoogte van het paviljoen begint hij aan de klim het strand op. De eerste trede van de trap naar boven is vrij hoog boven het zand. De stap is zo groot dat een jochie van een jaar of vier de trede niet op komt.	Frank dendert langs het joch en zorgt ervoor dat deze zijn balans verliest, omvalt en begint te huilen.	Frank zoekt binnen een tafel waar iedereen kan zitten en bestelt alvast wat te drinken. Een half uurtje later is iedereen aanwezig en bestellen ze wat te eten. Frank bestelt een biefstuk, medium rare met friet. Wanneer de biefstuk arriveert, blijkt hij bijna well-done te zijn. Frank roept de ober terug om het probleem uit te leggen en laat de biefstuk zien.	…	Frank	is	blij	als de ober reageert op een	vriendelijke en correcte manier
39	Cynthia is op het werk de penningmeester van de personeelsvereniging. Het is een initiatief van en voor werknemers en ze organiseren af en toe gezellige uitjes. Daarnaast verwelkomen ze nieuwe collega's met een cadeautje. Eén keer per jaar innen ze daarvoor vrijwillig contributie van iedereen. Cynthia heeft vorige week een mailtje uitgestuurd met het verzoek te betalen. Ondertussen heeft bijna iedereen het geld overgemaakt.	Cynthia stopt het geld in de kluis, dan maakt ze een rapport op en zorgt dat iedereen het kan inzien en controleren.	De week daarop moet Cynthia naar de dokter. Ze is al een poosje erg vermoeid en heeft weinig eetlust. Ze heeft daarom vorige week bij de dokter bloed laten prikken. De doktersassistente belde gisteren dat ze de volgende ochtend langs moest komen. Het is standaard procedure dit soort nieuws niet aan de telefoon te vertellen, maar ze is toch nerveus.	…	Cynthia	is	opgelucht	als uiteindelijk blijkt dat	de resultaten goed zijn, ze is kerngezond
40	Koen rijdt met de auto door de binnenstad. Het is druk op de weg en er komt een onoverzichtelijke kruising aan. Fietsers en auto's moeten hier de trambaan over vanwege werkzaamheden. Een oudere man op de fiets wankelt onzeker de trambaan op. Het is duidelijk dat de man in de war is en niet goed weet wat hij moet doen. De man komt bijna volledig tot stilstand recht voor Koen zijn auto.	Koen toetert kwaad en scheurt rakelings langs de oude man, in zijn spiegel ziet Koen de man omvallen.	Drie straten verder slaat Koen rechstaf richting zijn appartement. Hij parkeert een straat verderop waar bijna altijd wel plaats is. Koen zet de motor af en graait zijn tas van de achterbank. Nadat Koen de auto op slot heeft gedaan zwaait hij zijn tas over zijn schouder. Bij de entree van zijn gebouw ziet hij vanuit zijn ooghoek iemand aan komen lopen.	…	Koen	is	laaiend	wanneer de jongen op hem afloopt	ineens Koens tas grijpt en hard wegrent
41	Nina staat 's ochtends vroeg op en komt gelijk in actie. Ze gaat vandaag weer naar huis en ze wil nog winkelen voor ze vliegt. Ze heeft in het huis van vrienden van vrienden gezeten. Ze kent ze verder niet, maar ze hebben haar gratis laten logeren. Nina komt de keuken binnen en trekt de koelkast open. Het lampje springt niet aan en Nina komt er achter dat er een stop is gesprongen.	Nina besluit het te negeren en gaat lekker winkelen terwijl de vriezer langzaam ontdooit en overal water lekt.	Later die dag rent Nina beladen met al haar bagage naar de trein. Ze springt net op tijd in de trein richting het vliegveld. De trein vertrekt op tijd maar tien minuten later staan ze ineens stil tussen de weilanden. Nina begint zich zorgen te maken dat ze te laat gaat komen. Een uur later dan gepland komt de trein eindelijk aan op Schiphol.	…	Nina	is	verbolgen	als ze aankomt op het vliegveld en	blijkt dat ze te laat is om nog te boarden
42	Thomas zit in de kroeg met vrienden. Het is een mannenavondje en ze drinken er lustig op los. Thomas heeft vakantie en zijn vriendin is een week bij haar ouders. Hij mist haar wel, maar aan de andere kant is wat vrijheid ook wel fijn. Als iedereen besluit naar huis te gaan blijft Thomas nog even hangen. Hij raakt aan de praat met een mooie vrouw aan de bar die hem wel ziet zitten.	Thomas verzwijgt dat hij een vriendin heeft en gaat later met haar mee naar huis, zijn vriendin hoeft niks te weten.	De volgende dag sleept Thomas zich rond twee uur naar de supermarkt. Hij heeft niks meer in huis en ondanks een lichte kater toch honger. Wanneer hij uit de supermarkt komt zet hij de tassen even neer. Hij heeft gelijk maar grote boodschappen gedaan en het weegt heel wat. Wanneer hij bij zijn flat aankomt, ziet hij iemand vlak voor zich de deur openen.	…	Thomas	is	verheugd	als de man omkijkt, even wacht en	de deur vriendelijk glimlachend open houdt
43	Leonie gaat naar de schouwburg voor een toneelvoorstelling. Het is druk, want het is een mooi stuk en er spelen veel bekende acteurs mee. Voor de kassa staat een enorme rij, waar Leonie achter aansluit. Plots hoort Leonie iemand verderop haar naam roepen. Verderop in de rij staat een vriend van Leonie naar haar te zwaaien. Hij wenkt Leonie en gebaart dat ze bij hem in de rij moet komen staan.	Leonie wil niet voordringen en gebaart naar haar vriend dat ze hem binnen wel ziet en wacht op haar beurt.	Na de voorstelling loopt Leonie naar de garderobe. Ze had een jas en een paraplu meegenomen, omdat er stevige buien waren voorspeld. Bij de garderobe staat personeel dat de jassen en dergelijk ophaalt. Leonie haalt het garderobenummer tevoorschijn dat ze had gekregen. Ze geeft het nummer aan het garderobepersoneel, dat op zoek gaat naar haar spullen.	…	Leonie	voelt	irritatie	wanneer het garderobeperson eel	pas na tien minuten haar spullen vindt en ze zeiknat wordt
44	Laura komt pas om een uur of acht uit een vergadering. Ze is moe, maar tevreden over de uitkomst. Ze besluit naar huis te lopen omdat het een mooie zomeravond is. Eenmaal uit de drukte van het centrum is het heerlijk rustig op straat. Een paar straten van haar huis ziet Laura het portier van een auto op een kier staan. Ze kijkt rond of er iemand in de buurt is, maar er is niemand.	Laura denkt even na en duikt dan gauw met haar kop in de auto, haalt de autoradio eruit en loopt gauw verder.	De volgende ochtend staat Laura vroeg op en gaat naar haar werk. Ze is nieuwsgierig naar de uitkomst van een subsidieaanvraag die ze heeft ingediend. Bij de vergadering van gisteren heeft ze geprobeerd de laatste twijfelaars over te halen. Het leek erop dat de aanvraag ingewilligd ging worden. Laura probeert gewoon te werken terwijl ze op nieuws wacht.	…	Laura	is	woedend	wanneer blijkt dat haar aanvraag	is afgekeurd om een enorm domme reden
45	Wendy komt wat laat aan bij een feestje van haar broer. Het is een soort housewarming. Haar broer heeft zowel vrienden en familie als collega's uitgenodigd. Het is een flink groot feest, maar Wendy haar broer had geen zin om drie keer iets te organiseren. Wendy wordt gespot door haar broer en her en der voorgesteld. Dan stelt haar broer haar voor aan een collega die een mismaakte hand heeft.	Wendy trekt een gezicht en weigert de uitgestoken hand, de man is duidelijk gekwetst, maar dat boeit haar niet.	Later op de avond staat Wendy met wat familieleden te kletsen. Een aantal neven en een tante zijn ook aanwezig. Wendy heeft ze al een poos niet gezien en ze praat ze bij over haar leven. Wanneer Wendy even naar de wc gaat en weer terugkomt, treft ze iedereen lachend aan. Ze stoppen abrupt als ze er weer bij komt staan en Wendy kijkt ze vragend aan.	…	Wendy	is	vrolijk	wanneer ze even later	ook bij de grap wordt betrokken en meelacht
46	Michael gaat met zijn vrienden naar een voetbalwedstrijd. Hun club speelt uit tegen de aartsrivaal. De sfeer is gespannen, want de winnaar heeft grote kans kampioen te worden. Ze komen in een vak met medesupporters te zitten. Voor aanvang wordt er om een minuut stilte gevraagd voor een overleden speler van de tegenpartij. Het is rumoerig in het vak en ook Michaels vrienden praten en lachen.	Michael maant zijn vrienden aan stil te zijn en tot Michaels genoegen volgt de rest van het vak hun voorbeeld.	Een week later gaat Michael op vakantie. Hij vliegt 's ochtends vroeg naar Cyprus, waar hij een week in de zon wil liggen. Op internet heeft hij een goedkoop appartementje geboekt. De foto's van het appartement waren wat onduidelijk, maar Michael kon de aanbieding niet laten gaan. Eenmaal geland op Cyprus huurt Michael een auto en rijdt naar het appartement.	…	Michael	is	furieus	wanneer bij aankomst blijkt dat het	appartement niet bestaat en hij nergens heen kan
47	David staat op de steiger op het punt om te beginnen te schuren. Het is halverwege de ochtend en het is al flink heet. Het is de derde week van deze klus en ze zijn met het laatste stuk bezig. Hij is alleen aan het werk aan deze kant, zijn collega's zijn achter bezig. Hij ziet door het raam een vrouw achter een bureau zitten. Haar telefoon gaat en ze neemt aan en begint een heel gesprek.	David gaat lekker schuren, en als ze boos wordt maakt hij een suggestief gebaar naar zijn kruis en gaat door.	Na de lunch klimt David weer de steiger op. Hij gaat verder met het schuurwerk, ondertussen is hij bij het laatste raam. Het werk is goed opgeschoten ondanks de hitte. David werkt gestaag door en al gauw is ook het laatste kozijn geschuurd. David zet de machine uit en terwijl hij zijn bezwete voorhoofd afveegt glipt de schuurmachine uit zijn vingers.	…	David	is	opgelucht	als hij ziet hoe het apparaat	niet van de steiger valt en nog heel is
48	Kim stapt 's middags in de tram naar huis. Ze checkt in met haar pas en loopt door naar achter om een plaatsje te zoeken. Achter Kim stapt ook nog een vrouw in, ze loopt achter haar aan. Ze is hoogzwanger en volgt Kim verder de tram in. Kim ziet achterin nog één vrije zitplaats en stevent er op af. Voordat Kim de zitplaats bereikt begint de tram schokkend weer te rijden.	Kim neemt plaats en blijft zitten ook al wordt de zwangere vrouw vlak naast haar alle kanten op geslingerd.	Eenmaal thuis neemt Kim de stapel post door. Er zit een zoveelste brief bij van de belastingdienst. Ze is al maanden bezig om haar aanslag gecorrigeerd te krijgen. De belastingdienst heeft een fout gemaakt en weigert die te herstellen. Ze hoopt dat het eindelijk is opgelost, ze heeft er genoeg van.	…	Kim	is	gefrustreerd	wanneer blijkt dat haar aanslag	nog steeds niet klopt
49	Kelly loopt door het park op weg naar een feestje. Het schemert al, maar het is nog redelijk licht. Het is een warme dag geweest en er liggen nog mensen op handdoeken op het gras. Kelly pakt haar telefoon om op te zoeken waar ze precies moet zijn. Dan ziet ze vanuit haar ooghoek een portemonnee liggen. Er is niemand te bekennen en Kelly pakt de portemonnee op die vol geld blijkt te zitten.	Kelly checkt of er iets met een naam en adres in zit en besluit de portemonnee netjes naar de politie te brengen.	Kelly vervolgt haar weg naar het feestje en gaat daarna nog even de kroeg in. De volgende ochtend komt ze redelijk op tijd uit bed. Gelukkig heeft ze geen kater want ze moet nog voor haar studie aan de slag vandaag. Ze zet de laptop aan en gaat dan eerst koffie maken. Als ze terug komt opent ze haar mail en ziet een mailtje van de studieadviseur.	…	Kelly	is	dolblij	als ze in de email leest dat ze	een herkansing mag doen en kan afstuderen

50	Adriana loopt door het bos op een zondagochtend. Het is vroeg in de herfst en de bladeren beginnen net te verkleuren. Ze heeft onderweg koffie gehaald bij de enige koffiezaak die op een zondagochtend open is. Terwijl ze wandelt, neemt ze af en toe een slokje. Ze loopt graag in dit bos, het is redelijk groot en ze komt doordeweeks niet veel buiten. Halverwege haar gewone route heeft ze haar koffie op.	Adriana houdt netjes de beker bij zich om op de parkeerplaats weg te gooien, zo blijft het bos voor iedereen schoon.	Een uur later loopt Adriana nog steeds in het bos. Ze is ondertussen een heel eind bij de auto vandaan, maar ze is nog lang niet moe. Dan komt haar voet ineens in een gat terecht en verzwikt ze haar enkel. Ze voelt een scherpe pijn en valt om omdat ze niet meer op dat been kan staan. De pijn bonst stug door in haar enkel en ze kan niet anders dan langs het pad blijven zitten.	…	Adriana	voelt	boosheid	als er even later iemand langskomt	en diegene haar vraag om hulp compleet negeert
51	Judith zit op haar fiets voor de supermarkt. Ze is met een groepje van drie vrienden en ze hangen maar wat. Terwijl ze wat dom ouwehoeren ziet Judith een hardloper aankomen. De loper is hevig bezweet en het is hem aan te zien dat hij tot het uiterste gaat. Judith en haar vrienden staan pal in het pad van de hardloper. Judith haar vrienden doen een paar stappen opzij om plaats te maken.	Judith zet haar fiets lekker nog dwarser en als de hardloper uitwijkt, rolt Judith expres ook een stukje die kant op.	Later die dag loopt Judith van een terrasje door de stad naar huis. Het is nog redelijk vroeg, maar het begint al te schemeren. De zomer loopt ten einde, maar de temperatuur is nog lekker. Judith steekt de brug over en merkt ineens dat haar portemonnee niet in haar zak zit. Ze zou zweren dat ze hem net nog had en draait zich om om te kijken of hij ergens ligt.	…	Judith	voelt	opluchting	als ze zich omdraait en	een man haar portemonnee teruggeeft
52	Kirsten hangt wat rond met haar vrienden in het park. Ze drinken biertjes en zijn wat aangeschoten en baldadig. Een van Kirstens vrienden die meedrinkt begint opmerkingen te maken over mensen die langslopen. Eerst zachtjes, maar later steeds harder. Plotseling loopt een donkere jongen langs het groepje vrienden. Kirstens vriend roept een racistische opmerking naar de jongen en daagt hem uit.	Kirsten is direct nuchter en eist verontwaardigd dat haar vriend onmiddellijk zijn excuses aanbiedt aan de jongen.	De volgende dag gaat Kirsten naar de elektronicazaak. Ze heeft al een tijd een Ultra HD-tv op het oog en vandaag wil ze hem kopen. De tv is erg duur en Kirsten heeft er lang voor gespaard. Ze heeft de achterbank van haar auto vast naar beneden geklapt om ruimte te maken. Aangekomen bij de winkel vraagt Kirsten direct een medewerker naar de tv die ze wil.	…	Kirsten	voelt	boosheid	wanneer in de winkel blijkt dat	de tv die ze wil niet meer op voorraad blijkt te zijn
53	Danny is aan het werk in de werkplaats van zijn fietsenzaak. Hij is bezig een achterwiel te vervangen. Het is halverwege de ochtend en nog lekker rustig. Wanneer de bel gaat maakt Danny snel af wat hij aan het doen was. Hij veegt zijn handen schoon en loopt dan snel naar voren. Er staat een vrouw met een hoofddoek te wachten en het blijkt al gauw dat haar Nederlands niet heel goed is.	Danny neemt rustig de tijd en met de paar woorden Marokkaans die hij kent komen ze er uiteindelijk samen uit.	Later die dag, na de lunch, worden er onderdelen geleverd. Danny tekent voor ontvangst en begint de pakketjes te sorteren. Er is één bepaald onderdeel waar hij al een hele tijd op zit te wachten. Het is een heel speciaal onderdeel voor een luxe mountainbike. Het onderdeel is al twee keer verkeerd geleverd en de klant begint ongeduldig te worden.	…	Danny	voelt	opluchting	dat het binnengekomen onderdeel	nu wel het goede blijkt te zijn, eindelijk!
54	Sarah heeft de nachtdienst op de verpleegafdeling. Het is tijd om een ronde te lopen en haar collega en zij doen elk de helft. Sarah controleert alle infusen en kijkt of iedereen goed slaapt. Ze gaat stilletjes van kamer tot kamer. Op één van de laatste kamers ziet ze een portemonnee liggen. Hij is van de oudere man die vanmiddag zijn kleinkinderen met hun rapporten op bezoek had.	Sarah bergt de portemonnee netjes op in het kastje en neemt zich voor de man te waarschuwen voorzichtiger te zijn.	Aan het einde van haar dienst gaat Sarah gauw richting huis. Eenmaal thuis zet ze een kop thee en zet haar laptop aan. Met de laptop in één hand en de thee in de andere loopt ze richting de woonkamer. Ze ziet door het voorraam dat de zon ondertussen op is gekomen. Dan struikelt ze ineens over de drempel en laat de laptop vallen en morst thee.	…	Sarah	constateert	opgelucht	dat de laptop als ze hem weer aanzet	het weer gewoon blijkt te doen, gelukkig!
55	Lieke zit met een vriendin op het terras. Het is donderdagmiddag en ze hebben allebei lekker vakantie. Haar vriendin moet naar het toilet, waar het waarschijnlijk stervensdruk zal zijn. Terwijl Lieke alleen zit komt een vriend van haar langsfietsen. Hij vertelt Lieke over een feestje vanavond en moet gelijk verder. Lieke wil wel heen, maar ze heeft eigenlijk al plannen gemaakt met die andere vriendin.	Lieke liegt dat ze een andere afspraak was vergeten zodat ze toch naar het feestje van die vriend kan.	Later die middag springt Lieke gauw onder de douche thuis. Onder het afdrogen bedenkt ze zich dat ze cash geld nodig zal hebben. Dat herinnert haar eraan dat haar salaris één dezer dagen binnen moet komen. Nadat ze zich heeft aangekleed rommelt ze in de la om de random reader te vinden. Eenmaal gevonden logt ze gauw in om haar saldo te checken.	…	Lieke	constateert	opgetogen	dat de salarisadminstrati e	heeft gezorgd dat haar salaris op tijd is dit keer
56	Sanne zit naar een voorstelling onder regie van een vriend te kijken. Het stuk is ongeveer halverwege en Sanne is al volledig uitgecheckt. De acteurs zijn niet bijster goed en het decor is amateuristisch. Ook het verhaal zelf kan niet echt boeien. Sanne heeft sterk de neiging om halverwege naar huis te gaan. Maar ze blijft toch tot de receptie en komt daar haar vriend, de regisseur, tegen.	Sanne schudt hem de hand en feliciteert hem van harte, toch zoveel mogelijk positief commentaar gevend.	Na de receptie loopt Sanne richting het dichtstbijzijnde grote plein. De trams rijden al niet meer en het begint steeds harder te regenen. Eenmaal bij het plein aangekomen wil ze een taxi aanhouden. Er zijn echter weinig taxi's en veel mensen die een taxi willen vanwege de regen. Eindelijk weet ze een taxi aan te houden en deze stopt vlakbij haar.	…	Sanne	is	opgetogen	wanneer de taxi vlak voor haar stopt	en ze in kan stappen voor ze doorweekt is
57	Stefan zit achter de computer op het werk. Hij is bezig concertkaartjes te bestellen voor hem en drie collega's in zijn team. Ze gaan wel vaker met z'n allen iets doen buiten het werk om. Het is ondertussen meer een vriendengroep dan collega's. Er is een nieuw teamlid dat er nog niet echt bij hoort. Dat nieuwe teamlid komt even later binnen en zegt ook fan te zijn van die band.	Stefan liegt en zegt dat ze toch niet gaan omdat de kaartjes te duur zijn en bestelt later alsnog de kaartjes.	Tegen half vijf besluit Stefan dat het leuk is geweest voor die week. Hij sluit zijn computer af en pakt zijn spullen bij elkaar. Voor vijven is hij op het station en wacht hij op de trein. Het nadeel van op tijd weggaan op vrijdag is dat hij niet de enige is. Het is hartstikke druk op het perron en hij ziet dat de binnenkomende trein ook al goed vol zit.	…	Stefan	is	opgetogen	wanneer in het gedrang voor de inagng	iemand hem vriendelijk voor laat gaan
58	Maria loopt met een ingepakt cadeau over straat. Ze is onderweg naar een kraamvisite bij een collega. Maria heeft niks met baby's en gelukkig heeft iemand anders het cadeau uitgezocht. Maria belt aan en het duurt even voordat de deur opengaat. De kamer zit vol met familie en vrienden die ze niet kent. De baby wordt uiteraard meteen getoond en Maria vindt het maar een lelijke baby.	Maria zegt dat natuurlijk niet en ze complimenteert de moeder met zo'n ontzettend schitterende baby.	Wanneer Maria bij de kraamvisite vandaan komt loopt ze het centrum in. Ze heeft honger en gaat op zoek naar iets warms voor de lunch. Ze schiet een döner tent binnen en bestelt een broodje döner. Ze gaat zitten en valt meteen aan wanneer het broodje klaar is. Bij de eerste hap valt er gelijk een enorme klodder saus uit de zijkant van het broodje.	…	Maria	is	opgelucht	als de klodder gemorste saus	op tafel valt en niet op haar nieuwe blouse
59	Martijn fietst door de stad naar de sportschool. Hij heeft een lange dag achter de rug en heeft zin om intensief te sporten. Martijn is halverwege wanneer hij door een erg smal steegje fietst. Een bejaarde man loopt langzaam in dezelfde richting door het steegje. In het voorbijgaan schampt Martijn met zijn fiets de oude man. De man komt ten val en Martijn hoort hem kermen van de pijn.	Martijn stopt direct, gaat terug naar de man, belt een ambulance en geeft de man zijn jas om hem warm te houden.	De volgende dag staat Martijn vroeg op. Hij heeft een sollicitatiegesprek bij een bedrijf voor een leidinggevende functie. Het bedrijf is gevestigd in een nabijgelegen stad, 20 kilometer verderop. Omdat Martijn geen rijbewijs heeft reist Martijn met openbaar vervoer. Hij wil voor de zekerheid een trein eerder nemen en zorgt dat hij op tijd op het station is.	…	Martijn	is	kwaad	wanneer hij op het perron komt en	hoort omroepen dat er geen treinen rijden
60	Ilse staat te wachten bij de pinautomaat. Er staat één man voor haar en niemand achter haar. Terwijl ze wacht kijkt Ilse wat op haar telefoon en bedenkt hoeveel geld ze op zal nemen. Vanuit haar ooghoek ziet Ilse dat de man voor haar bijna klaar is. Ilse stopt haar telefoon weg en pakt haar portemonnee. Als de man wegloopt, ziet Ilse een briefje van 50 vallen maar de man heeft niks door.	Ilse houdt zich lekker stil en steekt de 50 euro gauw in haar zak, eigen schuld, wat een domme vent!	Later die avond is Ilse onderweg naar huis. Ze bedenkt zich ineens dat ze niks meer voor het ontbijt in huis heeft. Ze kijkt hoe laat het is en gaat meteen wat sneller lopen. Er is gelukkig een Albert Heijn vlakbij, maar die gaat al bijna dicht. Als Ilse de hoek om loopt ziet ze hoe een medewerker een bord binnenhaalt en ze gaat nog sneller lopen.	…	Ilse	is	pissig	wanneer de medewerker haar ziet	haar vuil aankijkt en de deur snel sluit
61	Emma loopt over de Dam richting de Kalverstraat. Ze moet een verjaardagscadeautje halen voor een vriend. Het is gelukkig niet heel druk, maar het loopt al tegen sluitingstijd. Op de hoek van de Kalverstraat zit een blinde straatmuzikant. Hij speelt de sterren van de hemel en zingt met een doorleefde stem. Op de gitaarhoes voor hem ligt al heel wat kleingeld en zelfs een paar briefjes.	Emma buigt zich alsof ze wat geeft maar grist in plaats daarvan snel een handvol munten en briefjes mee.	Later die week is de verjaardag waarvoor ze op pad was. Er zijn al gauw 25 mensen en het feestje is in volle gang. Emma kent echter niet veel van de mensen die er zijn, en bekenden zijn er nog niet. Ze probeert zich in het gesprek van een groepje naast zich te mengen. Emma gaat erbij staan en wanneer een stilte valt doet ze ook een duit in het zakje.	…	Emma	voelt	woede	opkomen wanneer het groepje	haar aanstaart en vervolgens negeert
62	Jeffrey komt na een dag hard werken thuis. Net op het moment dat hij de voordeur achter zich dicht trekt begint het te onweren. Het bliksemt en begint hard te regenen. Dan ziet Jeffrey dat er die dag post is bezorgd bij hem. Tussen de folders en rekeningen die zijn bezorgd ziet Jeffrey een rouwkaart liggen. De kaart blijkt verkeerd bezorgd te zijn en eigenlijk bestemd voor zijn overburen.	Jeffrey trekt zijn jas weer aan en loopt door de regen naar het huis van zijn buren om de kaart netjes af te geven.	Het noodweer blijft die dag aanhouden. Jeffrey hoort dat het KNMI code rood afgeeft en aanraadt binnen te blijven. Het begint hard te hagelen en Jeffrey ziet buiten enorme hagelstenen uit de lucht vallen. Een hagelsteen zo groot als een golfbal landt in Jeffreys tuin en Jeffrey vreest voor zijn nieuwe auto. Zodra het droog is gaat Jeffrey de schade opnemen.	…	Jeffrey	voelt	frustratie	wanneer hij bij de auto aan komt lopen	en die onder de kleine deuken zit van de hagelstenen
63	De afdeling waar Wesley werkt heeft het heel erg druk. De opdrachten blijven maar binnenstromen. Daarom is er kort geleden een nieuwe medewerker aangenomen om het team te versterken. De nieuwe medewerker is een jong en knap meisje dat pas afgestudeerd is. Op een dag komt Wesley haar alleen tegen in het kopieerhok. Ze staat bij het kopieerapparaat en heeft duidelijk ruzie met het apparaat.	Wesley helpt haar en legt uit hoe het apparaat werkt, ze is duidelijk opgelucht en bedankt hem vriendelijk.	Grijnzend werkt Wesley die middag verder en gaat om vijf uur naar huis. Thuis aangekomen parkeert hij zijn auto en loopt naar de voordeur van zijn huis. Wesley hoopt dat bij de post de dvd zit die hij besteld had. Hij had de hele avond vrijgehouden om het nieuwe seizoen van de tv serie te kijken. Wesley doet de deur open en ziet een stapel post op de deurmat.	…	Wesley	is	geërgerd	wanneer hij de post doorbladert	de dvd er niet bijzit, maar wel een boete
64	Sandra heeft al een tijdje een conflict met haar buren. Ze zet haar auto vaak op de parkeerplek voor de deur van haar buren. Haar buurman en buurvrouw beweren echter dat die plek speciaal voor hun auto is. Op een dag hoort Sandra van de andere buren dat haar buurman verongelukt is. Sandra aarzelt niet en loopt direct naar het huis van haar buren. Ze belt aan en haar buurvrouw doet de deur open.	Sandra condoleert haar en biedt aan de komende dagen voor haar te koken en wat ze verder maar nodig heeft.	Sandra loopt weer naar huis en gaat verder met koken. Die avond pakt Sandra haar tablet en leest het financiële nieuws. Sandra heeft kort geleden veel van haar spaargeld in aandelen gestoken. Ze houdt sindsdien de markt goed in de gaten en nu maakt ze zich zorgen. Ze heeft slechte berichten gehoord en checkt de koersen van de aandelen in haar portfolio.	…	Sandra	is	woedend	als ze de gegevens doorleest	en haar aandelen niks meer waard blijken te zijn
