# Supplementary material for: Tracking Affective Language Comprehension: Simulating and Evaluating Character Affect in Morally Loaded Narratives
Source: Front Psychol. 2019 Feb 22;10:318. doi: 10.3389/fpsyg.2019.00318 (PMC6398452; doi:10.3389/fpsyg.2019.00318)
Supplement: DATA SHEET S3 — Observed averages in 100 ms bins zygomaticus major per condition for all three critical segments. [file Data_Sheet_3.pdf]

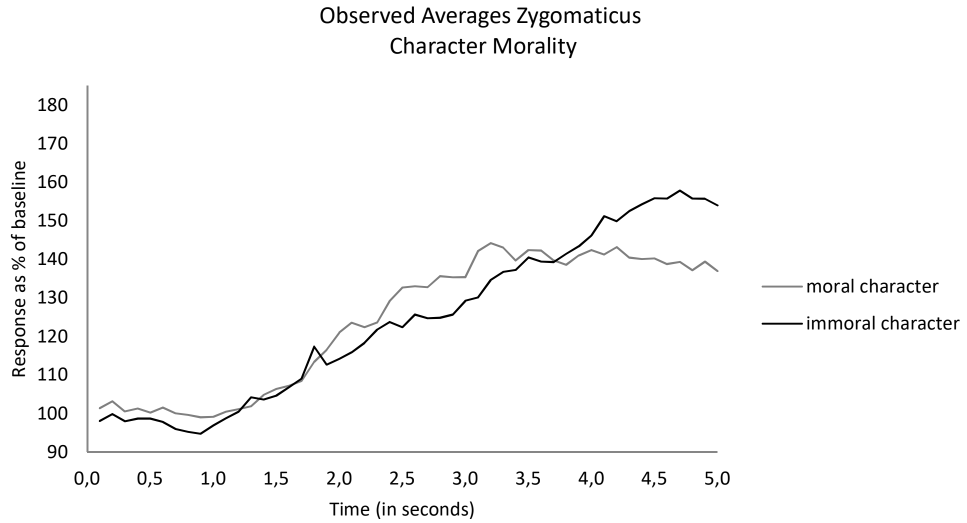

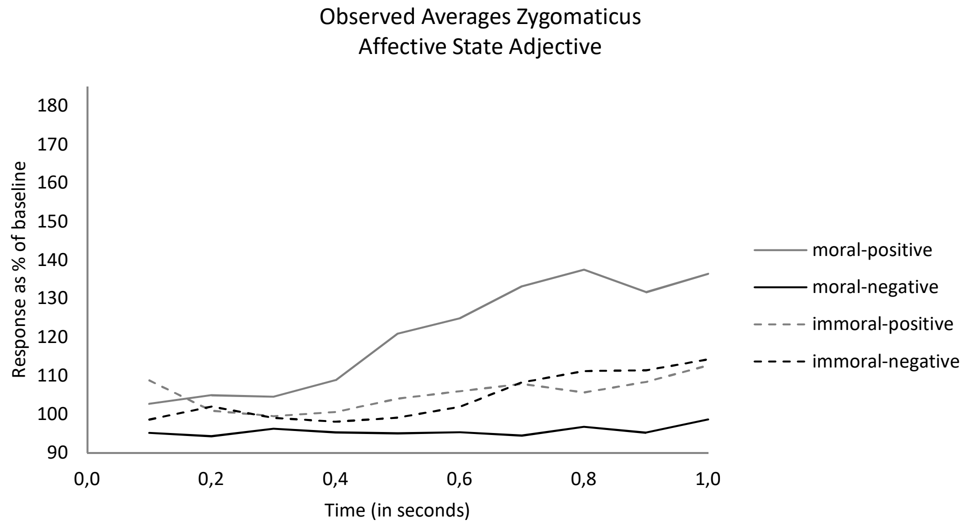

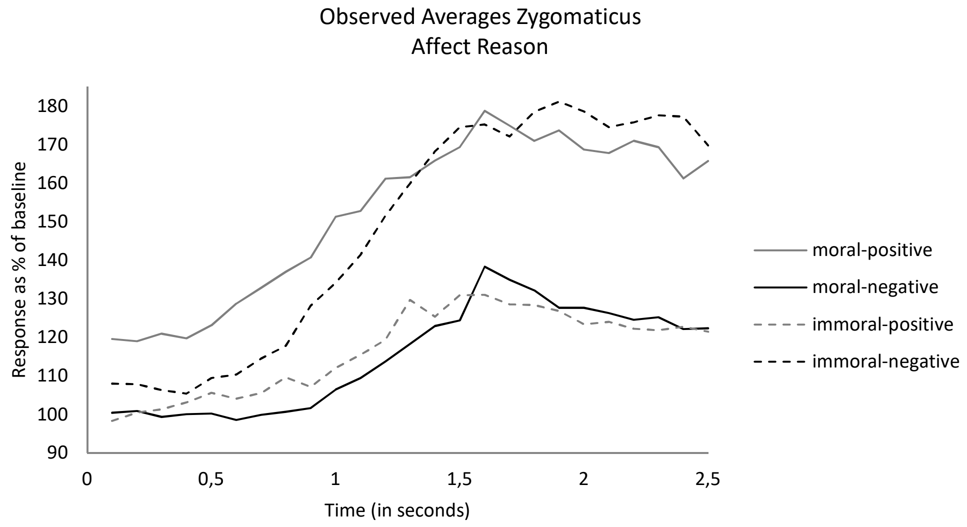

Supplementary Materials 3: Observed averages in 100 ms bins *zygomaticus major* per condition for all three critical segments
