# Supplementary figures and images for: Tracking Affective Language Comprehension: Simulating and Evaluating Character Affect in Morally Loaded Narratives
Source: Front Psychol. 2019 Feb 22;10:318. doi: 10.3389/fpsyg.2019.00318 (PMC6398452; doi:10.3389/fpsyg.2019.00318)

### Observed Average Corrugator Whole Trial

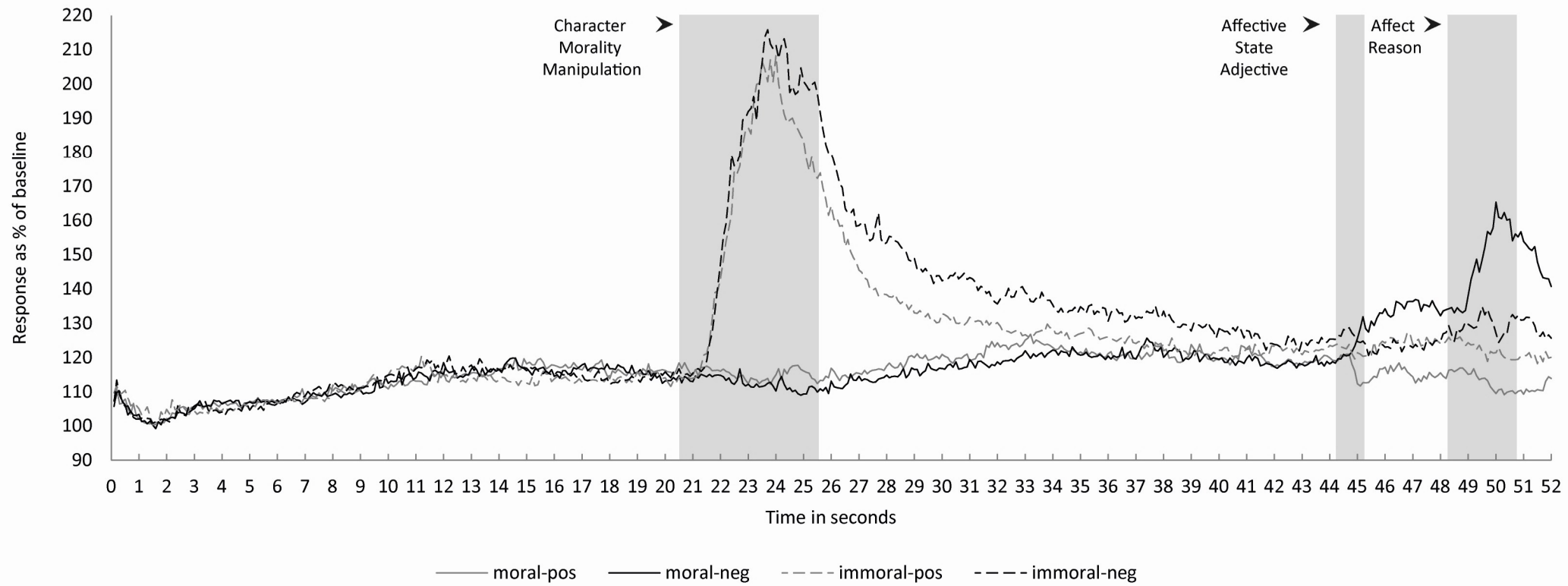

Supplement: DATA SHEET S4 — Whole trial observed average corrugator supercilii activity in 100ms bins over all trials, split into the four conditions (note: the positive/negative distinction is irrelevant at the character manipulation stage). [file Data_Sheet_4.PDF]
