# Supplementary material for: Tracking Affective Language Comprehension: Simulating and Evaluating Character Affect in Morally Loaded Narratives
Source: Front Psychol. 2019 Feb 22;10:318. doi: 10.3389/fpsyg.2019.00318 (PMC6398452; doi:10.3389/fpsyg.2019.00318)
Supplement: DATA SHEET S5 — Individual Differences. [file Data_Sheet_5.pdf]

Supplementary Materials 5 - Individual Differences

Parameter Estimates for Cognitive Empathy

Character Morality Manipulation

Estimates of Fixed Effectsa

Parameter	Estimate	Std. Error	df	t	Sig.	95% Confidence Interval	
						Lower Bound	Upper Bound
Immoral Character	247,724	44,172	60,440	5,608	0,000	159,381	336,067
Moral Character	128,351	44,171	60,439	2,906	0,005	40,008	216,693
Cognitive_Empathy	-4,031	12,265	59,888	-0,329	0,744	-28,565	20,503
Immoral Character * Cognitive_Empathy	-17,676	1,373	189828,890	-12,873	0,000	-20,367	-14,984
Moral Character * Cognitive_Empathy	0b	0,000	.	.	.	.	.

a Dependent Variable: Corrugator Response Character Morality.
b This parameter is set to zero because it is redundant.

Estimates of Fixed Effectsa

Parameter	Estimate	Std. Error	df	t	Sig.	95% Confidence Interval	
						Lower Bound	Upper Bound
Moral Character	128,351	44,171	60,439	2,906	0,005	40,008	216,693
Immoral Character	247,724	44,172	60,440	5,608	0,000	159,381	336,067
Cognitive_Empathy	-21,707	12,265	59,890	-1,770	0,082	-46,241	2,828
Moral Character * Cognitive_Empathy	17,676	1,373	189828,890	12,873	0,000	14,984	20,367
Immoral Character * Cognitive_Empathy	0b	0,000	.	.	.	.	.

a Dependent Variable: Corrugator Response Character Morality.
b This parameter is set to zero because it is redundant.

Affective State Adjective

Estimates of Fixed Effectsa

Parameter	Estimate	Std. Error	df	t	Sig.	95% Confidence Interval	
						Lower Bound	Upper Bound
other	140,975	14,672	59,764	9,609	0,000	111,625	170,325
moral-positive	135,451	15,148	67,924	8,942	0,000	105,223	165,680
Cognitive Empathy	-5,477	4,172	65,106	-1,313	0,194	-13,808	2,853
other * Cognitive Empathy	0,722	1,309	37742,522	0,551	0,582	-1,845	3,288
moral-positive * Cognitive Empathy	0b	0,000	.	.	.	.	.

a Dependent Variable: Corrugator Response Affect State Adjective.
b This parameter is set to zero because it is redundant.

Estimates of Fixed Effectsa

Parameter	Estimate	Std. Error	df	t	Sig.	95% Confidence Interval	
						Lower Bound	Upper Bound
other	130,627	14,668	59,750	8,906	0,000	101,284	159,969
moral-negative	166,540	15,152	68,043	10,991	0,000	136,304	196,775
Cognitive Empathy	-11,729	4,171	65,128	-2,812	0,006	-20,059	-3,399
other* Cognitive Empathy	9,054	1,312	37743,736	6,903	0,000	6,483	11,624
moral-negative * Cognitive Empathy	0b	0,000	.	.	.	.	.

a Dependent Variable: Corrugator Response Affect State Adjective.
b This parameter is set to zero because it is redundant.

Estimates of Fixed Effectsa

Parameter	Estimate	Std. Error	df	t	Sig.	95% Confidence Interval	
						Lower Bound	Upper Bound
other	142,725	14,667	59,759	9,731	0,000	113,384	172,067
immoral-positive	130,226	15,148	67,986	8,597	0,000	99,999	160,453
Cognitive Empathy	-2,473	4,170	65,056	-0,593	0,555	-10,800	5,854
other * Cognitive Empathy	-3,287	1,309	37741,604	-2,511	0,012	-5,853	-0,721
immoral-positive * Cognitive Empathy	0b	0,000	.	.	.	.	.

a Dependent Variable: Corrugator Response Affect State Adjective.
b This parameter is set to zero because it is redundant.

Estimates of Fixed Effectsa

Parameter	Estimate	Std. Error	df	t	Sig.	95% Confidence Interval	
						Lower Bound	Upper Bound
other	144,022	14,669	59,747	9,818	0,000	114,676	173,368
immoral-negative	126,087	15,156	68,081	8,319	0,000	95,844	156,330
Cognitive Empathy	-0,038	4,173	65,212	-0,009	0,993	-8,371	8,295
other * Cognitive Empathy	-6,508	1,315	37746,660	-4,948	0,000	-9,086	-3,930
immoral-negative * Cognitive Empathy	0b	0,000	.	.	.	.	.

a Dependent Variable: Corrugator Response Affect State Adjective.
b This parameter is set to zero because it is redundant.

Affect Reason

Estimates of Fixed Effectsa

Parameter	Estimate	Std. Error	df	t	Sig.	95% Confidence Interval	
						Lower Bound	Upper Bound
other	181,334	21,236	60,637	8,539	0,000	138,866	223,803
moral-positive	109,398	21,744	66,640	5,031	0,000	65,993	152,803
Cognitive Empathy	0,998	5,974	63,295	0,167	0,868	-10,938	12,934
other * Cognitive Empathy	-14,325	1,465	94769,927	-9,781	0,000	-17,195	-11,454
moral-positive * Cognitive Empathy	0b	0,000	.	.	.	.	.

a Dependent Variable: Corrugator Response Affect Reason.
b This parameter is set to zero because it is redundant.

Estimates of Fixed Effectsa

Parameter	Estimate	Std. Error	df	t	Sig.	95% Confidence Interval	
						Lower Bound	Upper Bound
other	136,973	21,234	60,580	6,451	0,000	94,507	179,439
moral-negative	242,545	21,728	66,408	11,163	0,000	199,169	285,921
Cognitive Empathy	-26,033	5,976	63,377	-4,356	0,000	-37,975	-14,092
other * Cognitive Empathy	21,714	1,467	94778,630	14,803	0,000	18,839	24,590
moral-negative * Cognitive Empathy	0b	0,000	.	.	.	.	.

a Dependent Variable: Corrugator Response Affect Reason.
b This parameter is set to zero because it is redundant.

Estimates of Fixed Effectsa

Parameter	Estimate	Std. Error	df	t	Sig.	95% Confidence Interval	
						Lower Bound	Upper Bound
other	175,559	21,240	60,689	8,265	0,000	133,082	218,036
immoral-positive	126,619	21,764	66,891	5,818	0,000	83,176	170,062
Cognitive Empathy	-1,272	5,972	63,243	-0,213	0,832	-13,206	10,662
other * Cognitive Empathy	-11,288	1,464	94766,431	-7,708	0,000	-14,158	-8,418
immoral-positive * Cognitive Empathy	0b	0,000	.	.	.	.	.

a Dependent Variable: Corrugator Response Affect Reason.
b This parameter is set to zero because it is redundant.

Estimates of Fixed Effectsa

Parameter	Estimate	Std. Error	df	t	Sig.	95% Confidence Interval	
						Lower Bound	Upper Bound
other	159,331	21,242	60,692	7,501	0,000	116,850	201,812
immoral-negative	175,171	21,777	67,025	8,044	0,000	131,705	218,638
Cognitive Empathy	-12,765	5,975	63,323	-2,136	0,037	-24,704	-0,827
other * Cognitive Empathy	4,048	1,472	94771,092	2,750	0,006	1,162	6,934
immoral-negative * Cognitive Empathy	0b	0,000	.	.	.	.	.

a Dependent Variable: Corrugator Response Affect Reason.
b This parameter is set to zero because it is redundant.

Parameter Estimates for Affective Empathy

Character Morality Manipulation

Estimates of Fixed Effectsa

Parameter	Estimate	Std. Error	df	t	Sig.	95% Confidence Interval	
						Lower Bou	Upper Bound
Immoral Character	252,879	33,459	61,157	7,558	0,000	185,976	319,782
Moral Character	116,822	33,459	61,155	3,491	0,001	49,920	183,725
Affective_Empathy	-0,895	10,691	60,214	-0,084	0,934	-22,278	20,488
Immoral Character * Affective_Empathy	-26,081	1,218	189990,054	-21,417	0,000	-28,468	-23,694
Moral Character * Affective_Empathy	0b	0,000	.	.	.	.	.

a Dependent Variable: Corrugator Response Character Morality.
b This parameter is set to zero because it is redundant.

Estimates of Fixed Effectsa

Parameter	Estimate	Std. Error	df	t	Sig.	95% Confidence Interval	
						Lower Bou	Upper Bound
Moral Character	116,822	33,459	61,155	3,491	0,001	49,920	183,725
Immoral Character	252,879	33,459	61,157	7,558	0,000	185,976	319,782
Affective_Empathy	-26,976	10,691	60,217	-2,523	0,014	-48,359	-5,593
Moral Character * Affective_Empathy	26,081	1,218	189990,054	21,417	0,000	23,694	28,468
Immoral Character * Affective_Empathy	0b	0,000	.	.	.	.	.

a Dependent Variable: Corrugator Response Character Morality.
b This parameter is set to zero because it is redundant.

Affective State Adjective

Estimates of Fixed Effectsa

Parameter	Estimate	Std. Error	df	t	Sig.	95% Confidence Interval	
						Lower Bou	Upper Bound
other	127,853	11,304	60,959	11,310	0,000	105,248	150,458
moral-positive	127,694	11,726	70,556	10,890	0,000	104,311	151,076
Affective Empathy	-3,830	3,696	65,767	-1,036	0,304	-11,209	3,549
other * Affective Empathy	2,603	1,161	37875,361	2,242	0,025	0,327	4,878
moral-positive * Affective Empathy	0b	0,000	.	.	.	.	.

a Dependent Variable: Corrugator Response Affective State Adjective.
b This parameter is set to zero because it is redundant.

Estimates of Fixed Effectsa

Parameter	Estimate	Std. Error	df	t	Sig.	95% Confidence Interval	
						Lower Bou	Upper Bound
other	128,804	11,304	60,979	11,395	0,000	106,201	151,408
moral-negative	124,768	11,733	70,759	10,634	0,000	101,373	148,164
Affective Empathy	0,061	3,696	65,833	0,016	0,987	-7,319	7,440
other * Affective Empathy	-2,578	1,164	37871,926	-2,214	0,027	-4,860	-0,296
moral-negative * Affective Empathy	0b	0,000	.	.	.	.	.

a Dependent Variable: Corrugator Response Affective State Adjective.
b This parameter is set to zero because it is redundant.

Estimates of Fixed Effectsa

Parameter	Estimate	Std. Error	df	t	Sig.	95% Confidence Interval	
						Lower Bou	Upper Bound
other	128,503	11,304	60,991	11,368	0,000	105,900	151,106
immoral-positive	125,679	11,730	70,723	10,714	0,000	102,288	149,071
Affective Empathy	-1,386	3,695	65,754	-0,375	0,709	-8,763	5,991
other * Affective Empathy	-0,651	1,161	37871,360	-0,560	0,575	-2,927	1,626
immoral-positive * Affective Empathy	0b	0,000	.	.	.	.	.

a Dependent Variable: Corrugator Response Affective State Adjective.
b This parameter is set to zero because it is redundant.

Estimates of Fixed Effectsa

Parameter	Estimate	Std. Error	df	t	Sig.	95% Confidence Interval	
						Lower Bou	Upper Bound
other	126,063	11,303	60,966	11,153	0,000	103,460	148,666
immoral-negative	133,052	11,734	70,783	11,339	0,000	109,655	156,450
Affective Empathy	-2,337	3,697	65,900	-0,632	0,529	-9,719	5,044
other * Affective Empathy	0,612	1,167	37878,357	0,525	0,600	-1,675	2,900
immoral-negative * Affective Empathy	0b	0,000	.	.	.	.	.

a Dependent Variable: Corrugator Response Affective State Adjective.
b This parameter is set to zero because it is redundant.

Affective Reason

Estimates of Fixed Effectsa

Parameter	Estimate	Std. Error	df	t	Sig.	95% Confidence Interval	
						Lower Bou	Upper Bound
other	147,614	16,557	61,925	8,915	0,000	114,515	180,712
moral-positive	96,205	17,039	69,414	5,646	0,000	62,217	130,193
Affective Empathy	5,496	5,352	63,853	1,027	0,308	-5,195	16,188
other * Affective Empathy	-9,943	1,299	94924,043	-7,651	0,000	-12,490	-7,396
moral-positive* Affective Empathy	0b	0,000	.	.	.	.	.

a Dependent Variable: Corrugator Response Affect Reason.
b This parameter is set to zero because it is redundant.

Estimates of Fixed Effectsa

Parameter	Estimate	Std. Error	df	t	Sig.	95% Confidence Interval	
						Lower Bou	Upper Bound
other	118,585	16,541	61,763	7,169	0,000	85,517	151,653
moral-negative	183,547	17,003	68,920	10,795	0,000	149,627	217,467
Affective Empathy	-10,956	5,351	63,921	-2,047	0,045	-21,647	-0,265
other * Affective Empathy	11,967	1,303	94935,838	9,184	0,000	9,413	14,521
moral-negatyive * Affective Empathy	0b	0,000	.	.	.	.	.

a Dependent Variable: Corrugator Response Affect Reason.
b This parameter is set to zero because it is redundant.

Estimates of Fixed Effectsa

Parameter	Estimate	Std. Error	df	t	Sig.	95% Confidence Interval	
						Lower Bou	Upper Bound
other	140,476	16,569	62,070	8,478	0,000	107,356	173,595
immoral-positive	117,245	17,071	69,910	6,868	0,000	83,196	151,293
Affective Empathy	1,601	5,352	63,845	0,299	0,766	-9,091	12,294
other * Affective Empathy	-4,706	1,300	94914,237	-3,619	0,000	-7,254	-2,157
immoral-positive * Affective Empathy	0b	0,000	.	.	.	.	.

a Dependent Variable: Corrugator Response Affect Reason.
b This parameter is set to zero because it is redundant.

Estimates of Fixed Effectsa

Parameter	Estimate	Std. Error	df	t	Sig.	95% Confidence Interval	
						Lower Bou	Upper Bound
other	132,153	16,572	62,096	7,974	0,000	99,027	165,279
immoral-negative	142,145	17,084	70,098	8,320	0,000	108,072	176,218
Affective Empathy	-4,018	5,354	63,920	-0,750	0,456	-14,714	6,679
other * Affective Empathy	2,793	1,307	94917,466	2,138	0,033	0,232	5,354
immorla-negative * Affective Empathy	0b	0,000	.	.	.	.	.

a Dependent Variable: Corrugator Response Affect Reason.
b This parameter is set to zero because it is redundant.

Parameter Estimates for Sympathy

Character Morality Manipulation

Estimates of Fixed Effectsa

Parameter	Estimate	Std. Error	df	t	Sig.	95% Confidence Interval Lower Bound	Upper Bound
Immoral Character	99,419	45,706	60,644	2,175	0,034	8,014	190,824
Moral Character	108,240	45,705	60,641	2,368	0,021	16,836	199,644
Sympathy	1,458	11,308	60,129	0,129	0,898	-21,159	24,076
Immoral Character * Sympathy	16,457	1,281	189990,009	12,852	0,000	13,947	18,967
Moral Character * Sympathy	0b	0,000	.	.	.	.	.

a Dependent Variable: Corrugator Response Character Morality.
b This parameter is set to zero because it is redundant.

Estimates of Fixed Effectsa

Parameter	Estimate	Std. Error	df	t	Sig.	95% Confidence Interval Lower Bound	Upper Bound
Moral Character	108,240	45,705	60,641	2,368	0,021	16,836	199,644
Immoral Character	99,419	45,706	60,644	2,175	0,034	8,014	190,824
Sympathy	17,915	11,308	60,132	1,584	0,118	-4,703	40,533
Moral Character * Sympathy	-16,457	1,281	189990,009	-12,852	0,000	-18,967	-13,947
Immoral Character * Sympathy	0b	0,000	.	.	.	.	.

a Dependent Variable: Corrugator Response Character Morality.
b This parameter is set to zero because it is redundant.

Affective State Adjective

Estimates of Fixed Effectsa

Parameter	Estimate	Std. Error	df	t	Sig.	95% Confidence Interval Lower Bound	Upper Bound
other	114,761	15,275	60,188	7,513	0,000	84,210	145,313
moral-positive	100,991	15,766	68,317	6,406	0,000	69,534	132,449
Sympathy	3,775	3,871	65,717	0,975	0,333	-3,955	11,505
other * Sympathy	-1,428	1,220	37887,369	-1,170	0,242	-3,820	0,964
moral-positive * Sympathy	0b	0,000	.	.	.	.	.

a Dependent Variable: Corrugator Response Affective State Adjective.
b This parameter is set to zero because it is redundant.

Estimates of Fixed Effectsa

Parameter	Estimate	Std. Error	df	t	Sig.	95% Confidence Interval Lower Bound	Upper Bound
other	116,432	15,271	60,187	7,624	0,000	85,887	146,976
moral-negative	95,933	15,768	68,424	6,084	0,000	64,471	127,394
Sympathy	7,288	3,871	65,760	1,883	0,064	-0,441	15,017
other * Sympathy	-6,106	1,223	37882,018	-4,994	0,000	-8,503	-3,710
moral-negative * Sympathy	0b	0,000	.	.	.	.	.

a Dependent Variable: Corrugator Response Affective State Adjective.
b This parameter is set to zero because it is redundant.

Estimates of Fixed Effectsa

Parameter	Estimate	Std. Error	df	t	Sig.	95% Confidence Interval Lower Bound	Upper Bound
other	107,386	15,272	60,193	7,032	0,000	76,840	137,933
immoral-positive	123,006	15,766	68,376	7,802	0,000	91,547	154,464
Sympathy	-0,390	3,870	65,682	-0,101	0,920	-8,118	7,337
other * Sympathy	4,135	1,220	37877,861	3,390	0,001	1,744	6,526
immoral-positive * Sympathy	0b	0,000	.	.	.	.	.

a Dependent Variable: Corrugator Response Affective State Adjective.
b This parameter is set to zero because it is redundant.

Estimates of Fixed Effectsa

Parameter	Estimate	Std. Error	df	t	Sig.	95% Confidence Interval Lower Bound	Upper Bound
other	106,683	15,271	60,177	6,986	0,000	76,139	137,226
immoral-negative	125,347	15,773	68,499	7,947	0,000	93,876	156,818
Sympathy	0,147	3,872	65,839	0,038	0,970	-7,584	7,879
other * Sympathy	3,400	1,226	37876,713	2,774	0,006	0,997	5,802
immoral-negative * Sympathy	0b	0,000	.	.	.	.	.

a Dependent Variable: Corrugator Response Affective State Adjective.
b This parameter is set to zero because it is redundant.

Affective Reason

Estimates of Fixed Effectsa

Parameter	Estimate	Std. Error	df	t	Sig.	95% Confidence Interval Lower Bound	Upper Bound
other	126,862	22,394	61,132	5,665	0,000	82,084	171,640
moral-positive	89,597	22,905	66,896	3,912	0,000	43,877	135,318
Sympathie	5,860	5,614	63,888	1,044	0,300	-5,355	17,075
other * Sympathie	-4,048	1,366	94935,411	-2,962	0,003	-6,726	-1,370
moral-positive * Sympathie	0b	0,000	.	.	.	.	.

a Dependent Variable: Corrugator Response Affect Reason.
b This parameter is set to zero because it is redundant.

Estimates of Fixed Effectsa

Parameter	Estimate	Std. Error	df	t	Sig.	95% Confidence Interval Lower Bound	Upper Bound
other	117,641	22,384	61,057	5,256	0,000	72,882	162,400
moral-negative	116,985	22,882	66,662	5,113	0,000	71,308	162,661
Sympathie	8,349	5,614	63,942	1,487	0,142	-2,867	19,565
other * Sympathie	-7,342	1,369	94943,976	-5,362	0,000	-10,025	-4,658
moral-negative * Sympathie	0b	0,000	.	.	.	.	.

a Dependent Variable: Corrugator Response Affect Reason.
b This parameter is set to zero because it is redundant.

Estimates of Fixed Effectsa

Parameter	Estimate	Std. Error	df	t	Sig.	95% Confidence Interval Lower Bound	Upper Bound
other	113,222	22,407	61,204	5,053	0,000	68,419	158,025
immoral-positive	130,460	22,933	67,138	5,689	0,000	84,689	176,232
Sympathie	-2,099	5,615	63,867	-0,374	0,710	-13,316	9,119
other * Sympathie	6,569	1,366	94920,970	4,809	0,000	3,891	9,246
immoral-positive * Sympathie	0b	0,000	.	.	.	.	.

a Dependent Variable: Corrugator Response Affect Reason.
b This parameter is set to zero because it is redundant.

Estimates of Fixed Effectsa

Parameter	Estimate	Std. Error	df	t	Sig.	95% Confidence Interval Lower Bound	Upper Bound
other	112,443	22,405	61,213	5,019	0,000	67,645	157,241
immoral-negative	133,161	22,942	67,288	5,804	0,000	87,371	178,950
Sympathie	-0,815	5,616	63,950	-0,145	0,885	-12,034	10,405
other * Sympathie	4,827	1,373	94915,957	3,516	0,000	2,136	7,517
immoral-negative * Sympathie	0b	0,000	.	.	.	.	.

a Dependent Variable: Corrugator Response Affect Reason.
b This parameter is set to zero because it is redundant.

Parameter Estimates for Fairness

Character Morality Manipulation

Estimates of Fixed Effectsa

Parameter	Estimate	Std. Error	df	t	Sig.	95% Confidence Interval	
						Lower Bou	Upper Bound
Immoral Character	11,282	45,975	60,986	0,245	0,807	-80,652	103,217
Moral Character	117,927	45,975	60,982	2,565	0,013	25,994	209,860
Fairness	-1,057	12,382	60,495	-0,085	0,932	-25,820	23,707
Immoral Character * Fairness	44,595	1,443	189860,222	30,913	0,000	41,768	47,423
Moral Character * Fairness	0b	0,000	.	.	.	.	.

a Dependent Variable: Corrugator Response Character Morality.
b This parameter is set to zero because it is redundant.

Estimates of Fixed Effectsa **MODEL DID NOT CONVERGE**

Parameter	Estimate	Std. Error	df	t	Sig.	95% Confidence Interval	
						Lower Bou	Upper Bound
Moral Character	119,157	70,736	.	1,685	.	.	.
Immoral Character	12,968	70,737	.	0,183	.	.	.
Fairness	1,042	141955,284	.	0,000	.	.	.
Moral Character * Fairness	-2,434	141955,286	.	0,000	.	.	.
Immoral Character * Fairness	0b	0,000	.	.	.	.	.

a Dependent Variable: Corrugator Response Character Morality.
b This parameter is set to zero because it is redundant.

Affective State Adjective

Estimates of Fixed Effectsa

Parameter	Estimate	Std. Error	df	t	Sig.	95% Confidence Interval	
						Lower Bou	Upper Bound
other	100,346	15,554	60,820	6,452	0,000	69,242	131,449
moral-positive	114,299	16,061	69,160	7,116	0,000	82,258	146,339
Fairness	0,474	4,294	66,641	0,110	0,912	-8,099	9,046
other * Fairness	6,016	1,375	37940,336	4,376	0,000	3,321	8,711
moral-positive * Fairness	0b	0,000	.	.	.	.	.

a Dependent Variable: Corrugator Response Affective State Adjective.
b This parameter is set to zero because it is redundant.

Estimates of Fixed Effectsa

Parameter	Estimate	Std. Error	df	t	Sig.	95% Confidence Interval	
						Lower Bou	Upper Bound
other	105,655	15,553	60,822	6,793	0,000	74,553	136,757
moral-negative	98,613	16,073	69,360	6,136	0,000	66,552	130,674
Fairness	7,192	4,296	66,773	1,674	0,099	-1,384	15,768
other * Fairness	-2,963	1,381	37938,615	-2,145	0,032	-5,670	-0,255
moral-negative * Fairness	0b	0,000	.	.	.	.	.

a Dependent Variable: Corrugator Response Affective State Adjective.
b This parameter is set to zero because it is redundant.

Estimates of Fixed Effectsa

Parameter	Estimate	Std. Error	df	t	Sig.	95% Confidence Interval	
						Lower Bou	Upper Bound
other	107,339	15,552	60,832	6,902	0,000	76,238	138,439
immoral-positive	93,697	16,068	69,303	5,831	0,000	61,646	125,749
Fairness	7,579	4,294	66,669	1,765	0,082	-0,993	16,151
other * Fairness	-3,492	1,377	37937,503	-2,536	0,011	-6,191	-0,794
immoral-positive * Fairness	0b	0,000	.	.	.	.	.

a Dependent Variable: Corrugator Response Affective State Adjective.
b This parameter is set to zero because it is redundant.

Estimates of Fixed Effectsa

Parameter	Estimate	Std. Error	df	t	Sig.	95% Confidence Interval	
						Lower Bou	Upper Bound
other	102,252	15,553	60,813	6,575	0,000	71,151	133,353
immoral-negative	108,867	16,075	69,396	6,773	0,000	76,803	140,932
Fairness	4,662	4,297	66,809	1,085	0,282	-3,916	13,239
other * Fairness	0,406	1,383	37939,813	0,294	0,769	-2,304	3,117
immoral-negative * Fairness	0b	0,000	.	.	.	.	.

a Dependent Variable: Corrugator Response Affective State Adjective.
b This parameter is set to zero because it is redundant.

Affective Reason

Estimates of Fixed Effectsa

Parameter	Estimate	Std. Error	df	t	Sig.	95% Confidence Interval	
						Lower Bou	Upper Bound
other	73,139	22,362	61,914	3,271	0,002	28,437	117,841
moral-positive	127,293	22,895	68,021	5,560	0,000	81,606	172,980
Fairness	-3,915	6,108	64,982	-0,641	0,524	-16,114	8,284
other * Fairness	20,556	1,539	94965,638	13,355	0,000	17,540	23,573
moral-positive * Fairness	0b	0,000	.	.	.	.	.

a Dependent Variable: Corrugator Response Affect Reason.
b This parameter is set to zero because it is redundant.

Estimates of Fixed Effectsa

Parameter	Estimate	Std. Error	df	t	Sig.	95% Confidence Interval	
						Lower Bou	Upper Bound
other	100,112	22,350	61,792	4,479	0,000	55,432	144,791
moral-negative	46,184	22,874	67,779	2,019	0,047	0,538	91,831
Fairness	28,419	6,109	65,043	4,652	0,000	16,218	40,619
other * Fairness	-22,532	1,546	94959,629	-14,578	0,000	-25,561	-19,502
moral-negative * Fairness	0b	0,000	.	.	.	.	.

a Dependent Variable: Corrugator Response Affect Reason.
b This parameter is set to zero because it is redundant.

Estimates of Fixed Effectsa

Parameter	Estimate	Std. Error	df	t	Sig.	95% Confidence Interval	
						Lower Bou	Upper Bound
other	84,519	22,389	62,017	3,775	0,000	39,764	129,274
immoral-positive	94,108	22,941	68,348	4,102	0,000	48,334	139,883
Fairness	7,651	6,114	65,027	1,251	0,215	-4,559	19,862
other * Fairness	5,049	1,543	94965,999	3,273	0,001	2,026	8,073
immoral-positive * Fairness	0b	0,000	.	.	.	.	.

a Dependent Variable: Corrugator Response Affect Reason.
b This parameter is set to zero because it is redundant.

Estimates of Fixed Effectsa

Parameter	Estimate	Std. Error	df	t	Sig.	95% Confidence Interval	
						Lower Bou	Upper Bound
other	89,597	22,392	62,031	4,001	0,000	44,838	134,357
immoral-negative	78,940	22,954	68,489	3,439	0,001	33,141	124,739
Fairness	13,921	6,116	65,111	2,276	0,026	1,706	26,136
other * Fairness	-3,318	1,549	94966,159	-2,141	0,032	-6,355	-0,281
immoral-negative * Fairness	0b	0,000	.	.	.	.	.

a Dependent Variable: Corrugator Response Affect Reason.
b This parameter is set to zero because it is redundant.
