# Supplementary material for: Tracking Affective Language Comprehension: Simulating and Evaluating Character Affect in Morally Loaded Narratives
Source: Front Psychol. 2019 Feb 22;10:318. doi: 10.3389/fpsyg.2019.00318 (PMC6398452; doi:10.3389/fpsyg.2019.00318)
Supplement: DATA SHEET S6 — Iterative model report for Character Morality Manipulation - Corrugator. Each line reports the assessment of model fit after adding a single predictor. [file Data_Sheet_6.pdf]

Supplementary Materials 6

Iterative model report for Character Morality Manipulation - Corrugator. Each line reports the assessment of model fit after adding a single predictor

**Character Morality Manipulation Corrugator**			**total cases**	**cases after baseline**	**data loss**	
			**192000**	**190050**	**1,02%**	
**Nr.**	**-2 LL**	**nr of parameters**	**p model fit (chi-square distribution**	**model comparison**	**predictor added**	**action**
Model 0	2529242,9590	2			empty model	
Model 1	2511977,0940	3	0,0000	better	Subject Random	keep
Model 2	2501228,5240	4	0,0000	better	Item Random	keep
Model 3	2501225,9120	5	0,1061	not better	Moral Linear Fixed	remove
Model 4	2498686,1960	5	0,0000	better	Immoral Linear Fixed	keep
Model 5	2491340,5110	7	0,0000	better	Immoral Linear Random (Subject Unstructured)	keep
Model 6	2491339,8290	8	0,4089	not better	Moral Quad Fixed	remove
Model 7	2490601,3430	8	0,0000	better	Immoral Quad Fixed	keep
Model 8	2488946,3750	11	0,0000	better	Immoral Quad Random (Subject Unstructured)	keep
Model 9	2488944,2780	12	0,1476	not better	Moral Cube Fixed	remove
Model 10	2488818,4930	12	0,0000	better	Immoral Cube Fixed	keep
Model 11	2488122,4730	16	0,0000	better	Immoral Cube Random (Subject Unstructured)	keep
Model 12	2487937,9420	17	0,0000	better	Character Morality	keep

Type III Tests of Fixed Effectsa

Source	Numerator df	Denominator df	F	Sig.
linear immoral	1	60,00362251	17,36376281	0,0001
quadratic immoral	1	61,84300291	38,94851803	0,0000
cubic immoral	1	60,02994377	9,505376991	0,0031
Character Morality	2	111,912766	369,1444592	0,0000

a. Dependent Variable: Corrugator Response Character Morality

Estimates of Fixed Effectsa

Parameter	Estimate	Std. Error	df	t	Sig.	95% Confidence Interval	
						Lower Bound	Upper Bound
Intercept (moral)	114,0609172	7,287592681	86,1383754	15,65138478	0,0000	99,57399443	128,54784
linear immoral	29,54865516	7,091135717	60,00362251	4,166984859	0,0001	15,36428945	43,73302088
quadratic immoral	-8,305789901	1,3308695	61,84300291	-6,24087478	0,0000	-10,96629448	-5,645285325
cubic immoral	-2,627777381	0,852322389	60,02994376	-3,083079141	0,0031	-4,332658508	-0,922896253
immoral	74,02181965	4,513030426	261,8649096	16,40179938	0,0000	65,13537198	82,90826733
moral	0b	0					

a. Dependent Variable: Corrugator Response Character Morality.
b. This parameter is set to zero because it is redundant.
Iterative model report for Affective State Adjective - Corrugator. Each line reports the assessment of model fit after adding a single predictor

**Affective State Adjective Corrugator**			**total cases**<br>**38400**	**cases after baseline**<br>**38010**	**data loss**<br>**1,02%**	
**Nr.**	**-2 LL**	**nr of parameters**	**p model fit (chi-square distribution**	**model comparison**	**predictor added**	**action**
Model 0	429997,7710	2			empty model	
Model 1	427395,6920	3	0,0000	better	Subject Random	keep
Model 2	425245,6870	4	0,0000	better	Item Random	keep
Model 3	425219,4060	5	0,0000	better	Linear moral-positive	keep
Model 4	425217,4670	6	0,1638	not better	Linear immoral-positive	remove
Model 5	425209,6530	6	0,0018	better	Linear immoral-negative	keep
Model 6	425183,6990	7	0,0000	better	Linear moral-negative	keep
Model 7	0,0000	0	0,0000	no convergence	Linear moral-positive random (Subject Unstructured)	remove
Model 8	425167,4670	9	0,0003	better	Linear immoral negative random (Subject Unstructured)	keep
Model 9	424963,5890	12	0,0000	better	Linear moral-negative random (Subject Unstructured)	keep
Model 10	424963,4200	13	0,6810	not better	Quadratic moral-positive	remove
Model 11	424963,4620	13	0,7216	not better	Quadratic immoral-positive	remove
Model 12	424963,5160	13	0,7870	not better	Quadratic immoral-negative	remove
Model 13	424963,5820	13	0,9333	not better	Quadratic moral-negative	remove
Model 14	424960,1240	13	0,0627	not better	Cubic moral-positive	remove
Model 15	424962,3230	13	0,2605	not better	Cubic immoral-positive	remove
Model 16	424963,5670	13	0,8821	not better	Cubic immoral-negative	remove
Model 17	424959,3080	13	0,0385	better	Cubic moral-negative	keep
Model 18	0,0000	0	0,0000	no convergence	Cubic moral-negative random (Subject Unstructured)	remove
Model 19	424957,3960	14	0,1667	not better	Character Morality	keep for interaction
Model 19a	424948,9060	15	0,0036	beter	Affective State Adjective Valence	keep
Model 19b	424947,9570	16	0,3300	niet beter	Character Morality * Affective State Adjective Valence	remove
Model 20	424950,8830	14	0,0037	beter	Valence	keep

Type III Tests of Fixed Effectsa

Source	Numerator df	Denominator df	F	Sig.
linear moral-positive	1	37578,69362	26,54331632	0,0000
linear moral-negative	1	244,524246	9,145819652	0,0028

linear immoral-negative	1	60,64117789	5,082119814	0,0278
cubic moral-negative	1	37578,69362	4,281241966	0,0385
Affective State Adjective Valence	2	125,799867	1080,455669	0,0000

a. Dependent Variable: Corrugator Response Affective State Adjective

Estimates of Fixed Effectsa

Parameter	Estimate	Std. Error	df	t	Sig.	95% Confidence Interval	
						Lower Bound	Upper Bound
Intercept	118,7422752	2,869403084	115,1181082	41,38222194	0,0000	113,058602	124,4259485
linear moral-positive	-11,7461506	2,279911418	37578,69362	-5,152020605	0,0000	-16,21483739	-7,277463821
linear moral-negative	23,24731728	7,687082222	244,524246	3,024205623	0,0028	8,105971931	38,38866264
linear immoral-negative	-7,17599349	3,183168191	60,64117789	-2,254355743	0,0278	-13,54189348	-0,810093497
cubic moral-negative	-77,34014094	37,37834535	37578,69362	-2,069116228	0,0385	-150,6026881	-4,077593757
Affective State Adjective Negative	6,697163925	2,288235171	252,7018633	2,926781306	0,0037	2,190722797	11,20360505
Affective State Adjective Positive	0b	0					

a. Dependent Variable: Corrugator Response Affective State Adjective

b. This parameter is set to zero because it is redundant.

Custom Hypothesis [a,b]

Contrast	Estimate	Std. Error	df	Test Value	t	Sig.	95% Confidence Interval	
							Lower Bound	Upper Bound
L1	-34,99346789	8,018056446	289,254292	0	-4,364332943	0,0000	-50,77459985	-19,21233593

a. linear moral-positive vs. linear moral-neg

b. Dependent Variable: Corrugator Response Affective State Adjective

Custom Hypothesis [a,b]

Contrast	Estimate	Std. Error	df	Test Value	t	Sig.	95% Confidence Interval	
							Lower Bound	Upper Bound
L1	-4,570157114	3,915425367	138,755984	0	-1,167218549	0,2451	-12,31176848	3,171454252

a. linear moral-positive vs. linear immoral-negative

b. Dependent Variable: Corrugator Response Affective State Adjective

Custom Hypothesis [a,b]

Contrast	Estimate	Std. Error	df	Test Value	t	Sig.	95% Confidence Interval	
							Lower Bound	Upper Bound
L1	30,42331077	7,539034406	266,3375988	0	4,035438643	0,0001	15,57962362	45,26699793

a. linear moral-negative vs. linear immoral-negative

b. Dependent Variable: Corrugator Response Affective State Adjective

Iterative model report for Affect Reason - Corrugator. Each line reports the assessment of model fit after adding a single predictor

**Affect Reason Corrugator**			**total cases 96000**	**cases after baseline 95025**	**data loss 1,02%**	
**Nr.**	**-2 LL**	**nr of parameters**	**p model fit (chi-square distribution**	**model comparison**	**predictor added**	**action**
Model 0	1180252,7400	2			empty model	
Model 1	1175596,4030	3	0,0000	better	Subject Random	keep
Model 2	1170435,9750	4	0,0000	better	Item Random	keep
Model 3	1170424,1930	5	0,0006	better	Linear moral-positive	keep
Model 4	1170417,3440	6	0,0089	better	Linear immoral-positive	keep
Model 5	1170416,6250	7	0,3965	not better	Linear immoral-negative	remove
Model 6	1170273,2890	7	0,0000	better	Linear moral-negative	keep
Model 7	1170272,6270	9	0,7182	not better	Linear moral-positive random (Subject Unstructured)	remove
Model 8	0,0000	0	0,0000	no convergence	Linear immoral-positive random (Subject Unstructured)	remove
Model 9	1169292,2290	9	0,0000	better	Linear moral-negative random (Subject Unstructured)	keep
Model 10	1169292,0000	10	0,6323	not better	Quadratic moral-positive	remove
Model 11	1169292,1640	10	0,7988	not better	Quadratic immoral-positive	remove
Model 12	1169292,0850	10	0,7043	not better	Quadratic immoral-negative	remove
Model 13	1169275,0800	10	0,0000	not better	Quadratic moral-negative	keep
Model 14	1168366,2210	13	0,0000	better	Quadratic moral-negative random (Subject Unstructured)	keep
Model 15	1168365,2660	14	0,3284	not better	Cubic moral-positive	remove
Model 16	1168366,2170	14	0,9496	not better	Cubic immoral-positive	remove
Model 17	1168363,8310	14	0,1221	not better	Cubic immoral-negative	remove
Model 18	1168358,2160	14	0,0047	better	Cubic moral-negative	keep
Model 19	1168306,2030	18	0,0000	better	Cubic moral-negative random (Subject Unstructured)	keep
Model 20	1168302,5470	19	0,0559	marginally better	Character Morality	keep for interaction
Model 21	1168257,6240	20	0,0000	better	Affect Reason Valence	keep
Model 22	1168233,0390	21	0,0000	better	Character Morality * Affect Reason Valence	keep

Type III Tests of Fixed Effectsa

Source	Numerator df	Denominator df	F	Sig.
linear moral-positive	1	94530,93478	12,09191101	0,0005
linear immoral-positive	1	94530,93478	7,028492925	0,0080
linear moral-negative	1	60,00128815	8,176338098	0,0058
quadratic moral-negative	1	66,28117166	3,025213019	0,0866
cubic moral-negative	1	60,07326121	2,830795905	0,0977
Character Morality * Affect Reason Valence	4	178,9017015	362,7751189	0,0000

a. Dependent Variable: Corrugator Response Affect Reason

Estimates of Fixed Effectsa

Parameter	Estimate	Std. Error	df	t	Sig.	95% Confidence Interval	
						Lower Bound	Upper Bound
linear moral-positive	-3,505587504	1,008122577	94530,93478	-3,477342521	0,0005	-5,481496121	-1,529678887
linear immoral-positive	-2,672664511	1,008122577	94530,93478	-2,651130499	0,0080	-4,648573128	-0,696755895
linear moral-negative	18,90708089	6,612185994	60,00128815	2,859429681	0,0058	5,680745483	32,13341629
quadratic moral-negative	-8,589610128	4,938504704	66,28117166	-1,739313951	0,0866	-18,44887224	1,269651989
cubic moral-negative	-7,095237618	4,217088014	60,07326121	-1,682496926	0,0977	-15,530458	1,339982768
immoral-negative	129,9054283	4,528289325	205,2627165	28,68752833	0,0000	120,9775051	138,8333515
moral-negative	154,6990289	4,601238504	218,8126486	33,62117151	0,0000	145,6306101	163,7674476
immoral-positive	122,1195034	4,527663594	205,1496025	26,97185885	0,0000	113,1927847	131,0462221
moral-positive	112,9416014	4,527663504	205,1495871	24,94478694	0,0000	104,0148829	121,86832

a. Dependent Variable: Corrugator Response Affect Reason

Pairwise Comparisonsa

(I) Character Morality * Affect Reason Valence		Mean Difference (I-	Std. Error	df	Sig.c	95% Confidence Interval for Diffe	
						Lower Bound	Upper Bound
	moral neg	-24,794*	4,765179	265,816784	0,000002	-37,460156	-12,127046
	immoral pos	7,785925	4,694179	250,325634	0,590652	-4,697722	20,269571
immoral neg	moral pos	16,964*	4,694185	250,327058	0,002188	4,480163	29,447491
	immoral neg	24,794*	4,765179	265,816784	0,000002	12,127046	37,460156
	immoral pos	32,580*	4,764585	265,684968	0,000000	19,914503	45,244548
moral neg	moral pos	41,757*	4,764589	265,685947	0,000000	29,092394	54,422461
	immoral neg	-7,785925	4,694179	250,325634	0,590652	-20,269571	4,697722
	moral neg	-32,580*	4,764585	265,684968	0,000000	-45,244548	-19,914503
immoral pos	moral pos	9,177902	4,693574	250,197493	0,309881	-3,304188	21,659992
	immoral neg	-16,964*	4,694185	250,327058	0,002188	-29,447491	-4,480163
	moral neg	-41,757*	4,764589	265,685947	0,000000	-54,422461	-29,092394
moral pos	immoral pos	-9,177902	4,693574	250,197493	0,309881	-21,659992	3,304188

Based on estimated marginal means

*. The mean difference is significant at the ,05 level.

a. Dependent Variable: Corrugator Response Affect Reason

c. Adjustment for multiple comparisons: Bonferroni.

Custom Hypothesis [a,b]

Contrast	Estimate	Std. Error	df	Test Value	t	Sig.	95% Confidence Interval	
							Lower Bound	Upper Bound
L1	-22,41266839	6,688595873	62,82319415	0	-3,350877944	0,0014	-35,77949691	-9,045839868

a. linear moral-positive vs linear moral-negative-neg

b. Dependent Variable: Corrugator Response Affect Reason

Custom Hypothesis [a,b]

Contrast	Estimate	Std. Error	df	Test Value	t	Sig.	95% Confidence Interval	
							Lower Bound	Upper Bound
L1	-0,832922992	1,425700621	94530,93478	0	-0,584220123	0,5591	-3,627279756	1,961433771

a. linear moral-positive vs linear immoral-positive

b. Dependent Variable: Corrugator Response Affect Reason

Custom Hypothesis [a,b]

Contrast	Estimate	Std. Error	df	Test Value	t	Sig.	95% Confidence Interval	
							Lower Bound	Upper Bound
L1	-21,5797454	6,688595873	62,82319415	0	-3,226349118	0,0020	-34,94657392	-8,212916875

a. linear moral-negative vs linear immoral-positive

b. Dependent Variable: Corrugator Response Affect Reason

Neutral Segment Corrugator

	total cases	cases after baseline	data loss
	**115200**	**114030**	**1,02%**

**Nr.**	**-2 LL**	**nr of parameters**	**p model fit (chi-square distribution**	**model comparison**	**predictor added**	**action**
Model 0	1330985,7590	2,0000			leeg model	
Model 1	1322707,2290	3,0000	0,0000	better	Subject Random	keep
Model 2	1315978,4920	4,0000	0,0000	better	Item Random	keep
Model 3	1315978,4590	5,0000	0,8559	not better	Character Morality	keep
Model 4	1315965,4270	6,0000	0,0003	better	Valence	keep
Model 5	1315950,0140	7,0000	0,0004	better	Character Morality * Valence	keep
Model 6	1310801,5320	8,0000	0,0000	better	Like Conditie * Event Valence Random (Subject VC)	keep

Type III Tests of Fixed Effectsa

Source	Numerator df	Denominator df	F	Sig.
like_by_valence	4	195,0944452	408,6125103	1,31744E-93

a. Dependent Variable: valence_response.

Estimates of Fixed Effectsa

Parameter	Estimate	Std. Error	df	t	Sig.	95% Confidence Interval	
						Lower Bound	Upper Bound
immoral-negative	124,153822	4,378025196	255,5314312	28,35840737	6,75697E-81	115,5322162	132,7754278
moral-negative	134,484349	4,377928806	255,5090063	30,71871538	9,2574E-88	125,8629294	143,1057685
immoral-positive	124,6348954	4,377775243	255,4731806	28,46991646	3,21382E-81	116,0137725	133,2560184
moral-positive	115,3116925	4,377775136	255,4731566	26,34025022	8,89349E-75	106,6905698	123,9328152

a. Dependent Variable: Corrugator Response Neutral Segment.

Custom Hypothesis Test 1 (immoral-pos vs. immoral-neg)

Contrast Estimatesa,b

Contrast	Estimate	Std. Error	df	Test Value	t	Sig.	95% Confidence Interval	
							Lower Bound	Upper Bound
L1	-0,481073443	5,053211251	382,3655523	0	-0,09520153	0,924204626	-10,41663441	9,454487519

a. immoral-pos vs. immoral-neg
b. Dependent Variable: valence_response

Custom Hypothesis Test 2 (moral-pos vs. moral-neg)

Contrast Estimatesa,b

Contrast	Estimate	Std. Error	df	Test Value	t	Sig.	95% Confidence Interval	
							Lower Bound	Upper Bound
L1	19,17265647	5,053127657	382,3404548	0	3,794215736	0,000172127	9,237257801	29,10805514

a. moral-pos vs. moral-neg
b. Dependent Variable: valence_response
